# Hikarchaeia demonstrate an intermediate stage in the methanogen-to-halophile transition

**DOI:** 10.1038/s41467-020-19200-2

**Published:** 2020-10-30

**Authors:** Joran Martijn, Max E. Schön, Anders E. Lind, Julian Vosseberg, Tom A. Williams, Anja Spang, Thijs J. G. Ettema

**Affiliations:** 1 Department of Medical Biochemistry and Microbiology, Uppsala University, Uppsala, Sweden; 2 Department of Biochemistry and Molecular Biology, Dalhousie University, Halifax, Canada; 3 Science for Life Laboratory, Department of Cell and Molecular Biology, Uppsala University, Uppsala, Sweden; 4 Theoretical Biology and Bioinformatics, Department of Biology, Utrecht University, Utrecht, The Netherlands; 5 School of Biological Sciences, University of Bristol, Bristol, UK; 6 NIOZ, Royal Netherlands Institute for Sea Research, Department of Marine Microbiology and Biogeochemistry, Utrecht University, Den Burg, The Netherlands; 7 Laboratory of Microbiology, Wageningen University and Research, Wageningen, The Netherlands

**Keywords:** Archaeal evolution, Archaeal genomics, Metagenomics

## Abstract

Halobacteria (henceforth: Haloarchaea) are predominantly aerobic halophiles that are thought to have evolved from anaerobic methanogens. This remarkable transformation most likely involved an extensive influx of bacterial genes. Whether it entailed a single massive transfer event or a gradual stream of transfers remains a matter of debate. To address this, genomes that descend from methanogen-to-halophile intermediates are necessary. Here, we present five such near-complete genomes of Marine Group IV archaea (Hikarchaeia), the closest known relatives of Haloarchaea. Their inclusion in gene tree-aware ancestral reconstructions reveals an intermediate stage that had already lost a large number of genes, including nearly all of those involved in methanogenesis and the Wood-Ljungdahl pathway. In contrast, the last Haloarchaea common ancestor gained a large number of genes and expanded its aerobic respiration and salt/UV resistance gene repertoire. Our results suggest that complex and gradual patterns of gain and loss shaped the methanogen-to-halophile transition.

## Abstract (web summary)

A study of the first genomes of the marine Hikarchaeia, the closest known relatives of Haloarchaea, is presented. Their inclusion in ancestral reconstructions unveils an intermediate stage in the evolutionary transition from ancestral anaerobic methanogens to modern day aerobic halophiles.

## Introduction

The Haloarchaea are obligate halophiles that thrive in moderate-to-extreme saline environments (e.g., brines, salt rocks, hypersaline soda lakes, and marine solar salterns)^1–3^. They often exhibit aerobic heterotrophic lifestyles^4^, although many facultative anaerobic members have been identified as well^5–7^. Most Haloarchaea have adapted to these high salt concentrations by employing a “salt-in” strategy, in which potassium and chloride ions are actively imported from the environment to equilibrate their osmotic strength and prevent water efflux^8–10^. To increase protein solubility and prevent protein aggregation in these hypersaline conditions, their genomes generally encode highly acidic proteomes^11,12^. Haloarchaea thriving in less extreme saline environments may alternatively employ a “salt-out” strategy, in which small organic compounds called compatible solutes are synthesized and accumulated^13–15^. While compatible solutes strengthen cellular osmolarity they do not interfere with the cellular machinery^16^. In salt lakes, ponds and deposits, high salinity is often accompanied with exposure to intense ultraviolet (UV) radiation^17^. Such radiation can damage DNA directly through DNA lesions at bipyrimidine dimer sites^18^ or indirectly via reactive oxygen species (ROS)^19,20^. To cope with UV stress, Haloarchaea encode a multitude of DNA damage prevention systems (e.g., carotenoid pigment biosynthesis and ROS scavenging) and DNA repair systems (photoreactivation, nucleotide excision repair, base excision repair, and homologous recombination)^17^.

Haloarchaea are most closely related to methanogens^21–24^ and are thought to have evolved from a methanogenic ancestor^21^. Their evolution must therefore have involved the wholescale remodeling of organismal ecology and metabolism from a strictly anaerobic methanogen to an aerobic halophilic heterotroph. Several studies have suggested that this transition was accompanied by extensive gene transfer from bacteria^25–29^. In two studies, over 1000 gene families (1089 and 1047, respectively) were observed in which haloarchaeal representatives formed monophyletic groups with bacterial homologs^30,31^. It was suggested that these gene families were acquired by the last Haloarchaea common ancestor (LHaCA) through a single massive gene transfer event from an ancestral bacterial lineage. However, subsequent analyses suggested that the inferred number of acquired genes by the LHaCA was highly sensitive to the taxon sampling and analytical method used: Becker et al.^32^ inferred 178 bacterial acquisitions using the same method as refs. ^30,31^ but with 75 instead of 10 haloarchaeal genomes^30^, while Groussin et al.^33^ inferred 215 gains using the same taxon sampling as a previous study^31^ but using an explicit phylogenetic birth-death model. It thus remains unclear whether the methanogen-to-halophile transition is characterized by a single, massive gene influx event or by a more gradual influx and efflux of gene families. Because the transition is represented by a single branch^22–24^ it is challenging to infer the nature of intermediate ancestors and the relative order of events along that transition. To test either hypothesis, genomic data of novel lineages that diverged from this single branch are necessary.

The Methanonatronarchaeia, first identified in the brine–seawater interface of the Shaban Deep in the Red Sea, represented one such candidate lineage^34^. Two strains were recently enriched from hypersaline (soda) lake sediments and were subsequently characterized and sequenced^35^. Initial phylogenetic and comparative analyses suggested that they represented a sister lineage of the Haloarchaea and exhibit features of both methanogens and extreme halophiles. With the methanogen-to-halophile branch broken in two, the authors inferred that certain key methanogenesis genes were lost prior to the Haloarchaea–Methanonatronarchaeia divergence, and that other methanogenesis genes were lost and genes for aerobic and heterotrophic pathways were acquired on the branch leading to the LHaCA^35^. However, the observed phylogenetic placement was recently challenged by Aouad et al.^36^, who suggested that Methanonatronarchaeia are placed as sister to all Methanotecta (i.e., the Haloarchaea, Marine Group IV, the methanogen class II as well as Methanophagales -ANME-1-, Synthrophoarchaeles and Archaeoglobi^24^; “Halobacterota” in the Genome Taxonomy Database) instead, questioning the inferences of Sorokin et al.^35,37^.

Here, we exploit the sequence data generated by the *Tara Oceans* initiative^38,39^ to reconstruct five metagenome assembled genomes (MAGs) of another candidate lineage, the Marine Group IV (MG-IV) archaea^40^. We perform in-depth phylogenomics to evaluate the relationships of Haloarchaea, Methanonatronarchaeia and MG-IV, and subsequently apply gene tree aware ancestral reconstruction to illuminate the methanogen-to-halophile transition. By estimating (i) the number of gene family acquisitions along the methanogen-to-halophile transition and Haloarchaea diversification, (ii) the phylogenetic origin of these acquisitions, and (iii) the gene family complement of ancestors along the transition, we identify major gene fluxes during the methanogen-to-halophile transition. We propose an updated scenario for haloarchaeal evolution in which the aerobic halophilic lifestyle gradually evolved from an methanogenic ancestor through step-wise gene gain and loss events.

## Results and discussion

### Exploration and genome reconstruction of MG IV archaea

**Fig. 1:**
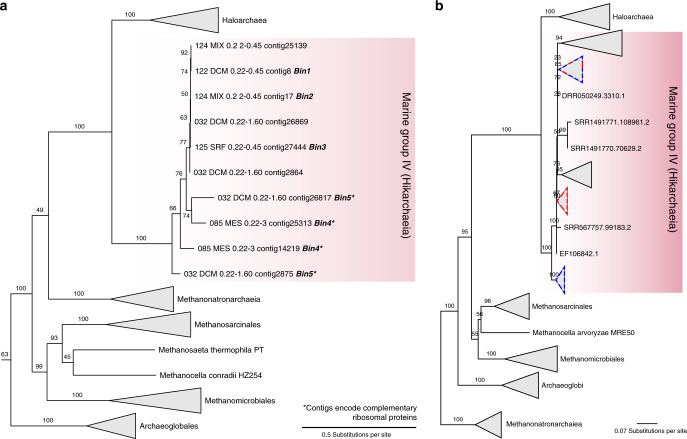
Metagenomic exploration of Hikarchaeia. **a** Phylogenetic diversity of Hikarchaeia compared to a reference set of Haloarchaea, Methanonatronarchaeia, and other Euryarchaeota based on an RP15 analysis (see “Methods”). Subtree of an RP15 tree representative of all archaea (Supplementary Fig. 1). **b** Unrooted phylogenetic tree of environmental 16S rRNA sequences of Hikarchaeia (see Supplementary Fig. 4 for full uncollapsed tree). Red and blue dash lines highlight Hikarchaeia clades that include the Hikarchaeia MAGs and the first reported Hikarchaeia 16S rRNA gene sequences^40^, respectively.

Marine Group IV archaea are a marine lineage defined by 16S rRNA genes that was first detected in the Antarctic Polar Front and was suggested to have a close phylogenetic affiliation with Haloarchaea^40^. We used this relatedness to screen all publicly available *Tara Oceans* metagenomic datasets for MG-IV representatives. We identified five datasets (Supplementary Table 1) and assembled them with a dedicated metagenome assembler^41^. A phylogenetic analysis of all contigs that encoded at least 5 out of 15 ribosomal proteins revealed that all contigs stemming from putative MG-IV lineages across the datasets formed a monophyletic sister group to Haloarchaea (Fig. 1a; Supplementary Fig. 1).

We reconstructed their genomes by binning contigs into five MAGs based on their differential coverage across samples, tetranucleotide frequency profiles and read-pair linkage information. The MAGs were highly complete and minimally redundant, relatively small (~1.2 Mb), moderately GC-rich (40–42%), had low nitrogen content in amino acid side chains (N-ARSC: ~3.1) and high coding density (93–94%) (Supplementary Table 2). Our analyses indicate that MG-IV comprise aerobic members, which use organic substrates potentially including aromatic compounds for organoheterotrophic growth (Supplementary Fig. 2, Supplementary Discussion). Streamlined genomes such as the ones exhibited by members of MG-IV are common in marine microbes and may represent an adaptation to the nutrient-poor marine environment^42–44^.

We explored the phylogenetic diversity of this clade in the sampled biosphere using the integrated microbial NGS platform (IMNGS) platform^45^ and found that this clade was exclusively found in marine environments (Supplementary Fig. 3). Phylogenetic analysis of the 16S rRNA gene that included the MAGs, IMNGS hits and reference MG-IV sequences^40^ confirmed the MG-IV identity of the MAGs and revealed a large MG-IV diversity (Fig. 1b; Supplementary Fig. 4). We propose the name Hikarchaeia for Marine Group IV (see species description below). The MAGs only covered a subset of the estimated Hikarchaeia diversity.

### Methanonatronarchaeia are not sister to the Haloarchaea

**Fig. 2:**
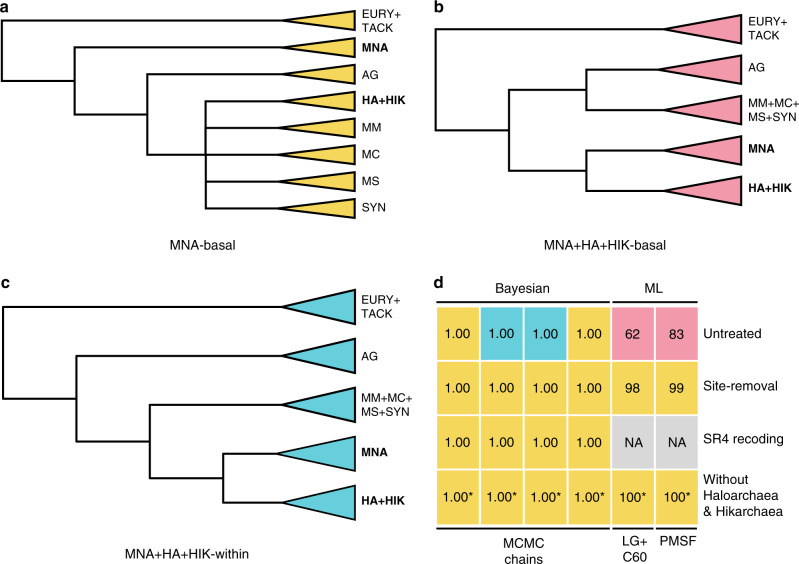
Resolving the branching order within Methanotecta. **a**–**c** Different species tree topologies for placement of the Methanonatronarchaeia. These trees represent the topological constraints used for the AU tests and branch support enumeration (Supplementary Table 4). Note that the polytomy in (**a**) does not necessarily indicate unresolved branch support in the inferred species trees, but rather freedom in the topological constraint during AU tests and branch support enumeration. EURY euryarchaeota, TACK TACK archaea, MNA methanonatronarchaeia, AG archaeoglobi, HA haloarchaea, HIK hikarchaeia, MM methanomicrobiales, MC methanocellales, MS methanosarcinales, SYN *Ca*. Syntrophoarchaea. **d** Overview of support values for the different topologies in (**a**–**c**) using several alignment treatments and phylogenetic models/reconstruction methods. Asterisk (*): due to omission of Hikarchaeia and Haloarchaea, MNA + HA + HIK-basal and MNA-basal topologies are equally well supported.

The availability of genomic data for the Hikarchaeia and Methanonatronarchaeia^35^, both thought to be close relatives of Haloarchaea, breaks the methanogen-to-halophile branch and allows us to evaluate the methanogen-to-halophile transition with unprecedented resolution. We first inferred the species tree by assembling a phylogenomics dataset of 56 ribosomal proteins conserved across Euryarchaeota^46^ and inferring Bayesian (CAT + GTR + Γ4) and maximum likelihood (ML; LG + C60 + F + Γ4 and its posterior mean site frequency (PMSF) approximation) trees. In all of our analyses, Hikarchaeia formed a clade with Haloarchaea to the exclusion of all other lineages, indicating that Hikarchaeia are the closest relatives of Haloarchaea sequenced to date (Supplementary Figs. 1 and 4–13). Three different, yet highly supported topologies were observed with respect to the placement of Methanonatronarchaeia (MNA) relative to the Hikarchaeia (HIK) and Haloarchaea (HA) clade. They were either placed as sister to all other Methanotecta (MNA-basal), as a sister to Hikarchaeia and Haloarchaea nested within the Methanotecta (MNA + HA + HIK-within) or with Hikarchaeia and Haloarchaea in a clade that is sister to all other Methanotecta (MNA + HA + HIK-basal; Fig. 2; Supplementary Figs. 5 and 6). These conflicting topologies may stem from phylogenetic artifacts such as long branch attraction and compositional bias. The Haloarchaea are situated on a long branch and the Methanonatronarchaeia may be attracted to the Haloarchaea due to similarly acidified amino acid compositions (Supplementary Figs. 14–16). Proteome acidification is a key adaptation of salt-in strategists to deal with extremely high salt concentrations^32,35^. Indeed, the exact phylogenetic placement of Methanonatronarchaeia is debated^35–37^.

We sought to alleviate phylogenetic artifacts by (i) removing the 50% sites that contribute most to overall alignment heterogeneity^47^ (Supplementary Fig. 17), (ii) recoding the data from a 20 to 4 character states^48^ or (iii) removing the long-branch Haloarchaea and Hikarchaeia. All phylogenetic analyses on treated alignments consistently separated the Methanonatronarchaeia from the Haloarchaea and Hikarchaeia and placed the Methanonatronarchaeia as a sister to all other Methanotecta with high support (Fig. 2d; Supplementary Figs. 7–9, 18, and 19). The Methanomicrobiales replaced the Methanonatronarchaeia as sister group of Haloarchaea and Hikarchaeia in all Bayesian trees with near maximum support (Supplementary Figs. 7 and 8) and an unsupported clade consisting of Methanomicrobiales, Methanocellales, Methanosarcinales, and Syntrophoarchaea replaced the Methanonatronarchaeia in both ML trees (Supplementary Fig. 9). Posterior predictive tests of the Bayesian analyses improved (as judged by comparison of *Z*-scores) upon site-removal and recoding treatments, yet worsened upon removal of Haloarchaea and Hikarchaeia (Supplementary Table 3). Approximately unbiased (AU) tests rejected the “MNA + HA + HIK-within” topology in most cases upon site removal or removal of Haloarchaea and Hikarchaeia. However, they were unable to reject the “MNA + HA + HIK-basal” topology (Supplementary Table 4).

These results are in line with Aouad et al.^36^, who observed that support for “MNA + HA” was replaced by support for “MNA-basal” upon progressive removal of fast evolving sites. In response to Aouad et al.^36^, Sorokin et al.^37^ argue that observed support for “MNA-basal” may have been an artifact induced by the removal of sites that contained putatively valuable phylogenetic signal. However, in our Bayesian inference of a similar phylogenomics dataset, two MCMC chains yielded a maximally supported “MNA-basal” topology (Fig. 2d, Supplementary Fig. 6), suggesting that even such fast-evolving sites contain “MNA-basal” signal. Sorokin et al.^37^ further argue that “MNA + HA” is well supported in the 16S rRNA gene phylogeny. However, our ML and Bayesian inference of the 16S and combined 16S and 23S rRNA genes consistently yielded trees in which MNA were separated from the Haloarchaea and Hikarchaeia (Supplementary Figs. 10–13). Here, the Methanonatronarchaeia branched as sister to all Methanotecta except the Archaeoglobi. These results collectively suggest that (i) Hikarchaeia are the closest sequenced relatives of Haloarchaea, and that (ii) while the placement of Methanonatronarchaeia is sensitive to the data and method used, they are not a sister lineage of Hikarchaeia and Haloarchaea but branch deeper within or as a sister to all other Methanotecta. This in turn implies that the Methanonatronarchaeia have adapted to halophily independently from Haloarchaea and are therefore not directly linked to the methanogen-to-halophile transition at the basis of the Haloarchaea.

### Gene tree aware ancestral reconstruction of the methanogen-to-halophile transition

Next we sought to reconstruct gene family histories along the Methanotecta species tree to gain insights into the methanogen-to-halophile transition. We opted to use the gene tree aware approach as implemented by ALE^49,50^. Briefly, amalgamated likelihood estimation (ALE) reconciles a sample of trees inferred from a gene family (e.g., a set of bootstrap trees) with a given species tree to infer ancestral events (originations, duplications, transfers and losses) and copy numbers on all nodes of that species tree. ALE improves on earlier gene tree-species tree reconciliation methods by estimating rates of gene duplication, transfer and loss directly from the data, and by accounting for the uncertainty in individual gene trees (which are often poorly resolved). The accuracy of the ancestral reconstruction therefore depends on the accuracy of the gene trees, the accuracy of the species tree and how well the taxon sampling of the species tree represents the extant diversity of the considered clades. We therefore opted to infer a new species tree that specifically focuses on the Methanotecta and includes 12 additional Haloarchaea genomes (Supplementary Data 1). Focussing specifically on Methanotecta in theory leads to a more accurate species tree because it effectively eliminates any potential artificial attraction from distant outgroups and leaves more phylogenetically informative sites after alignment trimming and heterogeneous site removal. Indeed, fewer heterogeneous sites needed to be removed to generate a minimally compositionally heterogeneous supermatrix alignment (30% vs. 50%, see below). We further omitted the Methanonatronarchaeia from the ancestral reconstruction analysis because we found that their placement in gene trees was often sensitive to phylogenetic artifacts: 14 out of 56 single gene phylogenies (25%) of ribosomal proteins (which are generally resistant to horizontal gene transfer) displayed a close grouping of Methanonatronarchaeia with Haloarchaea and Hikarchaeia with moderate-to-high support (UFB ≥ 70%; Supplementary Data 2).

We constructed the Methanotecta supermatrix alignment from the updated phylogenomics dataset, removed the top 30% sites that contribute most to overall alignment heterogeneity to alleviate potential artifacts (Supplementary Fig. 20) and inferred Bayesian (CAT + LG + Γ4) and ML (LG + C60 + F + Γ4 and its PMSF approximation) phylogenies. Compared to the Euryarchaeota species trees, the Methanotecta specific species trees differed most with respect to the nature of the Hik- and Haloarchaea-sister lineage. Whereas the original ML phylogenies suggested an unsupported clade of Methanomicrobiales, Methanocellales, Methanosarcinales, and Syntrophoarchaea (Supplementary Fig. 9), the new ML phylogenies nominated the Methanomicrobiales and Methanocellales as sister lineages (though the relationship between them was unclear; Supplementary Fig. 21). Similarly, whereas the original Bayesian phylogenies had near-maximum support for Methanomicrobiales as the sole sister clade (Supplementary Figs. 7 and 8), now they have near-maximum support for a clade consisting of Methanomicrobiales and Methanocellales (Supplementary Fig. 22). Curiously, this sister grouping was never seen before in archaeal phylogenies with relatively thorough Methanotecta sampling^22–24,51,52^. This topology may thus only be retrieved when combining a Methanotecta-only taxon sample with strategies to alleviate compositional bias. Indeed, when no attempt was made to alleviate bias, all phylogenetic inferences yielded a Methanomicrobiales-sister topology (Supplementary Figs. 23 and 24). But when attempting to alleviate bias via SR4 recoding, the Bayesian phylogenetic inference yielded again a Methanomicrobiales + Methanocellales-sister topology (Supplementary Fig. 25). We selected the Bayesian consensus tree based on the heterogeneous site removal supermatrix alignment as the species tree in the gene tree aware ancestral reconstruction.

**Fig. 3:**
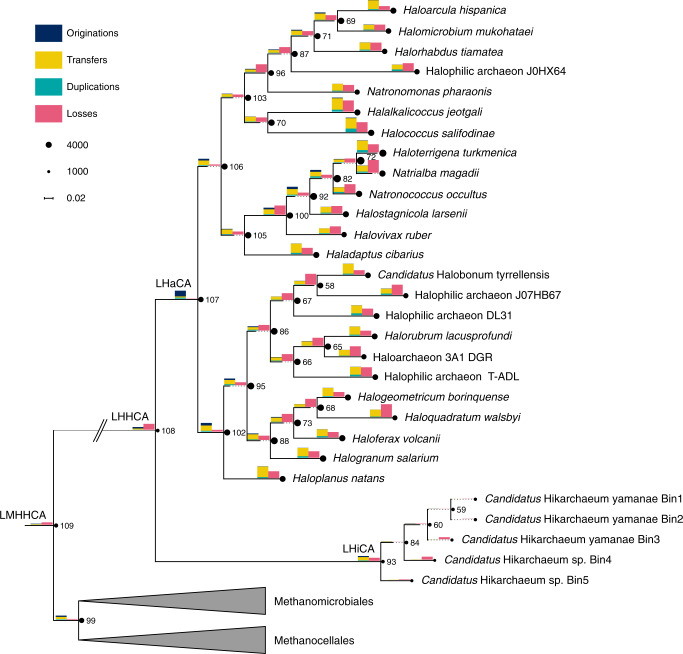
Ancestral reconstruction tree. Phylogenetic tree corresponds to the Bayesian consensus tree reconstructed from the Methanotecta supermatrix alignment (30% most heterogeneous sites removed; Supplementary Fig. 22). Nodes are annotated with their (inferred) genome size represented by the area of the black circles, branches are annotated with bars representing the number of duplications, transfers and origination as well as the number of losses (bar heights in legend correspond to 2000 such events each). For internal nodes, the number code identifying the branch in the ALE analysis is given. Some branches were extended with dotted lines to fit the width of the bars, and the branches leading to Methanomicrobiales and the ancestor of Haloarchaea and Hikarchaeia were collapsed for increased readability.

We clustered 158,269 proteins from 57 Methanotecta genomes (Supplementary Data 1) into 37,674 gene families based on the “eurNOGs” of the eggNOG database^53^. We took particular care to split fusion proteins into their hypothetical pre-fusion units such that their gene trees would reflect their independent phylogenetic histories (see “Methods”). Gene tree samples (1000 ultra-fast bootstraps) were inferred for all gene families and subsequently reconciled with the species tree to reconstruct their gene family histories. Note that ALE reports relative frequencies rather than integers for ancestral events and gene family copy numbers. These frequencies reflect their level of statistical support, analogous to how bootstraps quantify statistical support for clades in phylogenetic trees. Here we chose a relaxed minimum frequency threshold of 0.3 in order to capture the weakened signal that stems from the cumulative uncertainty introduced by alignment and tree reconstruction as well as the reconciliation. The gene tree aware ancestral reconstruction of the Methanotecta is summarized in Fig. 3.

In the presented species tree, the methanogen-to-halophile transition is characterized by two branches. The first embodies the evolution of the last common ancestor of Methanomicrobiales, Haloarchaea and Hikarchaeia (LMHHCA, i.e., the ancestral methanogen) to the last common ancestor of the Haloarchaea and Hikarchaeia (LHHCA) and featured 158 origination events (de novo genes or incoming gene transfers from outside the species tree), 127 transfers (from within the species tree) and 670 losses. This suggests that the methanogen-to-halophile transition involved a moderate amount of gene gain and a large amount of gene loss prior to the divergence of Haloarchaea and Hikarchaeia. The second branch leads to the LHaCA and is associated with 661 originations, as well as 131 transfers and 25 losses. This relatively large number of gains towards the LHaCA is reminiscent of the roughly thousand gains observed at this branch by previous studies^30,31^. However, these numbers cannot be directly compared. Originations encompass gene births and incoming gene transfers from anywhere outside the Methanotecta, while the thousand gains previously observed encompass only incoming transfers from bacteria. In addition, the taxonomic level of the gene families is inherently different. Still, we estimate that only a small fraction of the thousand families are represented in our set of LHaCA originations (see Supplementary Discussion). The comparison of both transition branches suggests that the bulk of gene loss occurred at the LMHHCA–LHHCA branch while the bulk of gene gain occurred at the LHHCA–LHaCA branch. However, it should be noted that the latter gene gain may be inflated: the Hikarchaeia genomes bear some hallmarks of genome streamlining (i.e., small, compact genomes, moderate AT-richness and low nitrogen content in amino acid side chains) and may exhibit accelerated gene loss rates. If so, any gene family that was acquired by the LHHCA and subsequently lost by the last Hikarchaeia common ancestor (LHiCA) during streamlining may falsely appear as an origination at the LHaCA. To which degree this inflates the number of gains at the LHaCA however is unclear. Finally, Haloarchaea diversification is characterized by continued origination events (albeit at lower rates), and generally increased rates of transfers and loss. A large fraction of these transfers occurred between Haloarchaea (Supplementary Data 3, Supplementary Fig. 26), which agrees with the observations of DeMaere et al.^54^.

In summary, these results suggest that the evolutionary history of Haloarchaea since the ancestral methanogen is characterized by a large loss of gene families at the LHHCA, a large gain at the LHaCA and complex, ongoing patterns of gene flow along the entire methanogen-to-halophile transition and subsequent Haloarchaea radiation.

### Inferred gene content of key ancestors along the methanogen-to-halophile transition

We screened the inferred gene complements of the LMHHCA, LHHCA, LHaCA, and LHiCA for gene families associated with protein complexes and metabolic pathways key to the methanogen-to-halophile transition. More specifically, we looked at gene families functionally related to methanogenesis, the Wood–Ljungdahl pathway (WLP), aerobic respiration, salt adaptation and UV resistance.

**Fig. 4:**
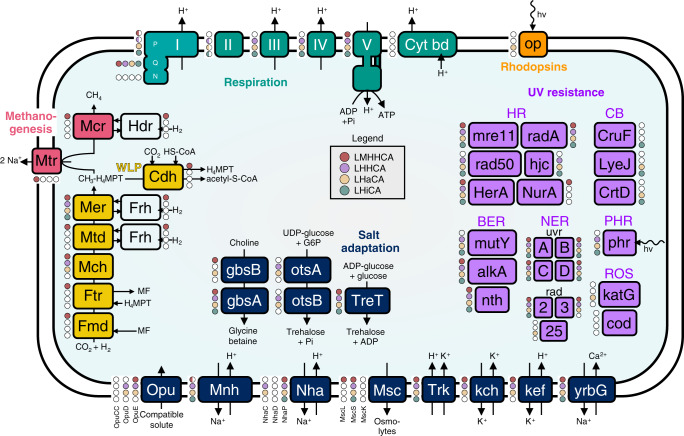
Ancestral gene content reconstruction. Inferred presence and absence of gene families functionally related to methanogenesis, aerobic respiration, salt adaptation, UV resistance, and rhodopsins in the LMHHCA, LHHCA, LHaCA, and LHiCA ancestors. A gene (or protein complex) was considered present if it (or at least half of the subunits) was inferred to be present by ALE with copy number ≥ 0.3. Note that genes homologous to components of the NADH dehydrogenase in LMHHCA may alternatively encode components of alternative [Ni–Fe] hydrogenases (Supplementary Data 4). Gene families encoding independently acting proteins but with similar names (e.g., NhaC and NhaD) are enumerated separately. See Supplementary Data 4 for exact copy number estimates. “I”: NADH dehydrogenase, “II”: Succinate dehydrogenase, “III”: Cytochrome bc1 complex, “IV”: Heme/copper-type terminal oxidase, “V”: ATP synthase, “Cyt bd”: Cytochrome bd-type oxidase. HR homologous recombination, BER base excision repair, NER nucleotide excision repair, CB carotene biosynthesis, PHR photoreactivation, ROS reactive oxygen species detoxification, WLP Wood–Ljungdahl Pathway.

Our analysis suggested that key genes related to methanogenesis and the WLP, as well as the Frh and Hdr hydrogenases, were represented in the LMHHCA by at least one gene family with moderate (“+”; copy number 0.65 < *x* < 1.00) or strong (“++”; copy number ≥ 1.00) ALE support (Fig. 4, Supplementary Datas 4–7, Supplementary Fig. 27). Only *fmdD* (formylmethanofuran dehydrogenase subunit D; 0KT6G), *ftr* (formylmethanofuran:tetrahydromethanopterin formyltransferase; 0KRCW) and *mtrA, -B, -G* and *E* (tetrahydromethanopterin S-methyl transferase subunits A, B, G, and E; 0KSJM, 0KTXF, 0KRG4, and 0KUPF) were inferred with weak (“+/−”; copy number 0.3 < *x* < 0.65) support. All of these genes were lost in the branch leading to the LHHCA, with the exception of *fmdE* (0KSBC), *mch* (0KRT9) and *mer* (0KRKG). While all three gene families were also inferred to be present in the LHaCA, only *mer* (0KRKG) was inferred present in the LHiCA. It is unclear how the respective encoded proteins function in Haloarchaea and Hikarchaeia, which do not encode a complete WLP. Together, these results indicate that the LHHCA had already foregone its ancestral methanogenic lifestyle prior to the large gain of genes at the LHaCA. This is in contrast with results by Nelson-Sathi et al.^30^, whom put forward the hypothesis that the recipient of the mass influx of genes at the LHaCA was a methanogen.

Among the considered genes related to aerobic respiration, only those affiliated with the Nuo-type dehydrogenase (Complex I) and the A_1_A_0_-type ATP synthase (Complex V) were inferred to be present in the LMHHCA with sufficient support (Supplementary Fig. 27, Supplementary Data 5). More specifically, Complex I was represented by *nuoH*, *-L*, *-B*, *-C* and *D* (++) and by *nuoK* (+/−) and while there was no support for *nuoE*, *-F*, and *G*, there was moderate support for the *frhB/fpoF* gene family (0KRJG_COG1035). Though it may seem odd that complex I was only partially represented (lacking *nuoA, -I, -J, -M, -N*), it is important to realize that we can only reconstruct histories of gene families that are present in the sampled species. These apparent missing genes may have thus been represented by other gene families that are either not sampled or have gone extinct. Complex V was represented by nearly all subunits with weak-to-strong support, lacking only *atpH*. The LHHCA was inferred to encode mostly the same Complex I and V genes as its parent but additionally displayed weak-to-moderate support for the presence of several Complex II (*sdhA* 0KRBI+, *sdhB* 0KTQ6_COG0479+/−), III (*petB* 0M0DJ+/− 0KS3S+/−) and IV (*coxA* 0KS8F_COG0843+/−, *coxB* 0KUT6+, *coxC* 0KS8F_COG1845+) genes. The LHaCA encoded the same set of respiration genes as its parent and appeared to have gained *nuoN* (0KRW8++), *atpH* (0M02F+), *nuoM* (0KRVF+/−) and *nuoF* (0KSCY_COG1894+/−). It further acquired additional *coxA* (0M1BG+/−), *coxB* (0M11Q+, 0KXWQ+/−) and *coxC* (0M0D4++) families. Most respiration families had their copy number support either maintained or increased since the LHHCA. The LHiCA also encoded much of the same respiration genes as the LHHCA (only losing support for *nuoK/mnhC*—0KUEK) but additionally acquired many more such genes through incoming transfers.

Many considered gene families related to salt adaptation were inferred to be already present in the LMHHCA (Supplementary Fig. 28, Supplementary Data 5). In particular, salt-in systems such as K^+^ channels (*kch*), K^+^ transporters (*trk*, *kef*), mechanosensitive ion channels (*mscS*, *mscL*) and Na^+^/Ca^2+^ exchangers (*yrbG*) were represented by at least one gene family with strong support (Supplementary Data 4). Presence of a complete multi-subunit type Na^+^/H^+^ antiporter (Mnh) was less well supported: only subunits *mnhA* (0KS6Y_COG1009++, 0KS3D++), *mnhB* (0KS6Y_COG2111+/−), *mnhD* (0KS97++), and *mnhG* (0KU2W+/−) were inferred, and it is unclear whether the *mnhA* families functionally encode a MnhA or NuoL subunit (*mnhA* and *nuoL* share a deep homology^55^). Salt-out systems such as compatible solute import (OpuE) and synthesis (GbsA, GbsB, and TreT) were inferred with weak-to-moderate support. The transition to the LHHCA observed the loss of *mscL* (0KU64) and two *kefB/kefC* families (0KRTC and 0KS73) as well as the gain of a putative *mscS* (0M0XP) family. Interestingly, it also experienced a weakly supported gain of its missing subunits (*mnhC, mnhE*, and *mnhF*) and an overall copy number increase of the retained Mnh antiporter families. Compatible solute synthesis genes *gbsA* (glycine-betaine synthesis; 0KRCS) and *treT* (trehalose synthesis; 0KSHX) saw their copy numbers increased as well, though *gbsB* (0KRJV) lost nearly all of its copy number support. The LHaCA encoded the same set of salt adaptation genes (except *yrbG* family 0KSUJ) as its LHHCA parent, typically with maintained or increased copy number support. Most notably, all subunits of the Mnh antiporter, and most considered salt-out genes were now represented with at least one family with moderate support. As the LHHCA putatively had a similar salt adaptation gene set as the LHaCA, it may already have had a halophilic or halo-tolerant lifestyle. The LHiCA experienced considerable loss in salt adaptation genes, losing support for 18 out 36 gene families present with at least weak support in the LHHCA. However, the LHiCA was the only investigated ancestor that represented all Mnh antiporter subunits with strong support.

Several gene families putatively involved in UV resistance were inferred to be present in the LMHHCA with at least moderate support (Supplementary Data 5). These included *uvrA* (0KRIU++), *uvrB* (0KS67+), *uvrC* (0KRFH++) and *uvrD* (0KS06_COG0210++) of the bacterial type nucleotide excision repair (NER) system, *rad2* (0KRD9++) and *rad3* (0KRHU++) of the eukaryotic type NER system, *alkA* (0KUYX+) and *nth* (0KS7U+) of the base excision repair system, *herA* (0KRAJ++), *mre11* (0KTCS++), *nurA* (0KV1H++), and *radA* (0KSB4++) of the homologous recombination (HR) machinery (Supplementary Fig. 28). The transition to the LHHCA saw the gain of photoreactivation gene *phr* (0KRHQ++), HR gene *hjc* (0KTCF++), and NER gene *rad50* (0KRAS++) and loss of HR gene *nurA* (0KV1H). In addition, a number of gene families that lost support in the transition (*uvrD:* 0KS06_COG0210, *herA:* 0KRAJ, *mre11:* 0KTCS) seemed to be replaced by other gene families potentially encoding functionally similar genes (*uvrD:* 0KVIT+, *herA*: 0KRUB+ and 0KZQC++, *mre11*: 0KS7S++). The LHaCA retained all UV resistance gene families, which had their inferred copy numbers either maintained or increased. It further expanded its gene repertoire with the eukaryotic NER gene *rad25* (0KWFG++) and additional homologous families of *rad3* (0KZZK++, 0KRUX+). The LHiCA on the other hand lost support for several redundant gene families (*uvrD*—0KYTF*, herA*—0KSFN) and its only *rad3* family (0KRHU) but appeared to gain carotene biosynthesis related families *lyeJ* (0KSPB+), *crtD* (0KRIE+), and *cruF* (0KUXK++) by means of transfer.

In summary, our analysis indicates that the LMHHCA featured a complete Wood–Ljungdahl and methanogenesis pathway. It further already encoded a number of genes associated with salt adaptation and UV resistance, though strong support for salt-out strategy genes was lacking. The LHHCA in contrast showed no support for nearly all genes associated with the above-mentioned anaerobic pathways and associated hydrogenases and experienced a net expansion of salt adaptation and UV resistance genes, though some gene families were lost as well. The exact timing of the emergence of the complex II–IV genes remains unclear. Though it is clear that the LMHHCA lacked them and the LHaCA encoded most of them, their appearance in the species tree was neither strongly tied to the LHHCA nor to the LHaCA. The LHaCA retained all of its parent’s genes under investigation (except one *yrbG* family) and appears to have experienced a general expansion and proliferation of aerobic respiration, salt adaptation, and UV resistance genes.

### Gene families acquired by Haloarchaea and Hikarchaeia cannot be traced to a single donor lineage

**Fig. 5:**
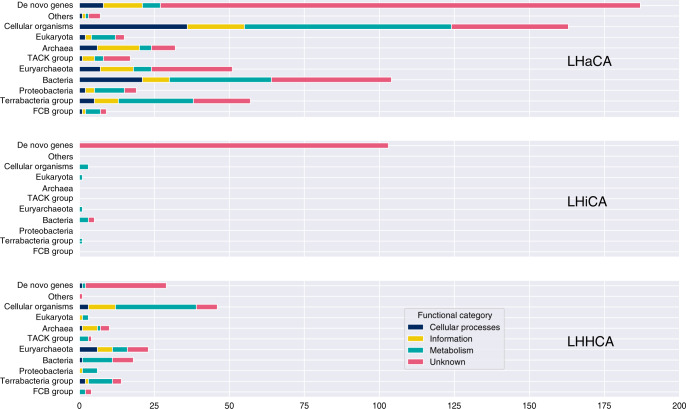
Tracing the origins of haloarchaeal gene content. Stacked bar charts showing the inferred taxonomic affiliation of originations at the LHaCA, LHiCA, and LHHCA. Each bar corresponds to an inferred taxonomic group, where the length of the bar indicates the number of clusters with that taxonomic annotation and the colors indicate the broad functional annotation of the clusters. For visualization purposes, we also summarized more specific placements into broader taxonomic groups.

We further investigated 3457 gene families that were inferred originations on the branch leading to the LHHCA or on any branch after it. These genes can be seen as new in the species trees, which in turn can either correspond to incoming transfers from outside the species tree, or to *bona fide* gene births. For each such gene family, we attempted to identify the phylogenetic source of the transfer by placing the sequences onto the corresponding eggNOG reference gene trees containing homologs across the tree of life (but with Methanotecta sequences removed to avoid self-placement). The taxonomic label of the branch with the best placement score (see “Methods”) was assumed as the phylogenetic affiliation (Fig. 5, Supplementary Fig. 29, Supplementary Data 8). These branches represent the best candidates for the immediate donor lineage of incoming transfers or targets of outgoing transfers after either gene birth or after another, previous, incoming transfer. Gene families for which no homologs outside our sampled species were present in the database were considered de novo genes. Note that some families may have been transferred in from lineages that were not sampled in the eggNOG database and would thus be falsely inferred as de novo genes. To evaluate the accuracy of our placement analysis, we compared the placement of each gene family sequence that is also present in the eggNOGs with the phylogenetic position of its corresponding Methanotecta sequence in the reference trees. In 84% of gene families, all such sequences were placed in the same position (same sister sequence), in 13% at least some sequences were placed identically, and in 3% none of the sequences were placed in the same position. This is not unexpected considering uncertainties in single protein tree phylogenies and suggests that the inferred placements discussed below are accurate.

At the LHaCA node, we inferred a total of 661 originations of which 187 were de novo genes, 194 were transfers from Bacteria, 102 from other Archaea and 163 transfers could only be assigned to “cellular organisms” (Fig. 5, Supplementary Data 8). Broad taxonomic labels such as “cellular organisms” or “Bacteria” point toward an unclear phylogenetic affiliation and possibly a convoluted gene history with several successive transfers. The many transfers from Bacteria were associated with diverse groups as Terrabacteria, the FCB group or Proteobacteria. The two child nodes of the LHaCA both show high amounts of gene gain as well, with 188 and 286 inferred originations (Fig. 5). Both in these two and in more recent internal Haloarchaea nodes, the originations seem to originate from various source lineages (Supplementary Fig. 29). This suggests that the expansion of the Haloarchaea gene repertoire has been an ongoing process that also continued after the divergence of Haloarchaea, rather than the result of a single transfer event from one particular source at the Haloarchaea stem. Originations at the LHiCA node were in their great majority (103 out of 114) identified as de novo genes without any detectable homologs in the reference data. However, it has to be noted that originations could be overestimated if donor lineages were missing from our reference set. The LHHCA showed a pattern more similar to the LHaCA, with 29 de novo genes, 43 transfers from Bacteria, 37 from Archaea and 46 transfers assigned to cellular organisms.

Unexpectedly, we identified Eukaryota as the closest taxon in 60 cases (Supplementary Data 8). Further investigation into these cases revealed two possible reasons. In a single case (0Z4PB) homologs from those genes were only present in Haloarchaea and eukaryotes. Provided this protein cluster represents a true gene family it would infer the exciting scenario of a gene birth in one lineage followed by an interdomain transfer to the other. However, eukaryote placements were observed in gene families that were generally larger (large in terms of number of member sequences, *p* = 2.4e−7, Supplementary Fig. 30; and alignment length, *p* = 8.07e−6) than families associated with other taxonomic groups. These eukaryote placements may thus possibly be the result of placement artifacts stemming from the more error-prone alignments and tree reconstructions of large gene families^56^.

In summary, our analyses did not identify any signal pointing towards a single bacterial donor lineage for all identified transfers for neither the LHaCA nor the LHHCA, in agreement with previous studies^30,31^. We interpret this as evidence for the occurrence of numerous transfer events from various distinct lineages that are spread out over evolutionary time along the stem leading to LHaCA and LHiCA and, to some degree, throughout haloarchaeal diversification.

## Conclusions

**Fig. 6:**
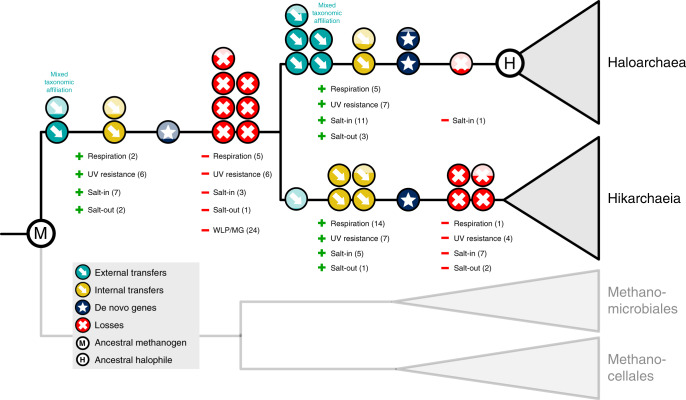
Model of methanogen-to-halophile transition. Cartoon depiction of gene flow associated with the methanogen-to-halophile transition. Arrows, stars and crosses on the branches indicate an approximate quantification of number of transfers (external: from outside the species tree, internal: from inside the species tree), de novo genes and gene losses, respectively. Number of gene gains (transfer or origination ≥ 0.3; +) and losses (loss ≥ 0.3; −) for gene families related to respiration, methanogenesis and salt and UV adaptation are indicated below the branches. WLP Wood–Ljungdahl pathway, MG methanogenesis. Their detailed gene flows are visualized in Supplementary Figs. 24 and 25.

We present five high quality draft genomes of Hikarchaeia (previously MG-IV), which unambiguously branch as a close sister group to the Haloarchaea in all our phylogenomic analyses. These analyses lend further support to the notion that the recently discovered Methanonatronarchaeia most likely do not form a sister lineage to Haloarchaea and Hikarchaeia but instead form an early diverging or sister lineage of the Methanotecta^35,36^. The inclusion of Hikarchaeia in gene tree aware ancestral genome reconstructions revealed an intermediate stage of the methanogen-to-halophile transition (the LHHCA) which was subject to heavy gene loss, including the loss of the methanogenesis and Wood–Ljungdahl pathways. The subsequent branch towards the LHaCA experienced a large gain in genes and included a net expansion of aerobic respiration, salt adaptation, and UV resistance genes (Fig. 6, Supplementary Figs. 27 and 28). Finally, the subsequent Haloarchaea diversification is characterized by continued gene gain and loss. Our observations do not fit with a single massive gene transfer scenario as was previously suggested^30,31^ but are best explained by a more gradual scenario in which a continuous process of gene gain (gene transfers of heterogeneous origin and gene births) and loss shaped the methanogen-to-halophile transition.

### Candidatus “Hikarchaeum yamanae” (sp. nov, gen. nov.)

“Hika” translates to “sea” in the Yahgan (Yámana) language of the Tierra del Fuego natives. Tierra del Fuego is relatively close to sample TARA_085_MES_0.22-3 (Supplementary Table 1) and to the Drake Passage, one of the sites where MG-IV archaea were first discovered. This organism lives in aerobic marine waters, can be found in both deep and shallow samples and has a global distribution. It encodes genes for aerobic respiration, likely uses organic substrates potentially including aromatic compounds for organoheterotrophic growth and exhibits streamlined genomes (around 1.2 Mb) with a low GC content (around 41%). It is currently not in culture and only known from environmental sequencing, with five MAGs presented here. Bin1 is the designated type MAG.

### Description of Hikarchaeaceae (fam. nov.)

Description is the same as for the genus Hikarchaeum. Suff. -aceae, ending to denote a family. Type genus: Hikarchaeum gen. nov.

### Description of Hikarchaeales (ord. nov.)

Description is the same as for the genus Hikarchaeum. Suff. -ales, ending to denote an order. Type genus: Hikarchaeum gen. nov.

### Description of Hikarchaeia (class nov.)

Description is the same as for the genus Hikarchaeum. Suff. -ia, ending to denote a class. Type order: Hikarchaeales ord. nov.

## Methods

### Sample selection and sequence data

All publicly available Tara Oceans assemblies^38^ were screened with the RP15 pipeline^57^ for presence of candidate MG-IV lineages (here defined as a well-supported sister lineage to reference Haloarchaea)^58^. Briefly, the RP15 pipeline approximates the phylogenetic position of all taxa present in a metagenome assembly for which at least 5 out of 15 well conserved ribosomal proteins are encoded on a single contig. In the end, five assemblies (highlighted in Supplementary Table 1) were selected. The raw sequence data associated with these and other samples (Supplementary Table 1) were downloaded from the Tara Oceans project ERP001736 on the EBI Metagenomics portal.

### Metagenome assembly and binning

All reads were preprocessed as described by Martijn et al.^58^ (“Metagenome assembly and binning of Pacific Ocean samples”). SeqPrep^59^ was used to merge overlapping read pairs into single reads and remove read-through Illumina adapters for all selected samples but 125_SRF_0.22-0.45, and Trimmomatic v0.35^60^ was used to remove residual Illumina adapters, trim low quality base-calls at starts and ends of reads, remove short reads and finally remove reads that had a low average phred score. The overall quality and presence of adapter sequences of processed and unprocessed reads was assessed with FASTQC v0.11.4^61^.

The preprocessed metagenomes of the five selected samples were re-assembled with metaSPAdes^41^, a mode of SPAdes with k-mers 21,33,55,77. 122_DCM_0.22-0.45 and 125_SRF_0.22-0.45 were assembled with version 3.7.0, 085_MES_0.22-3 and 124_MIX_0.22-0.45 with 3.8.0, and 032_DCM_0.22-1.60 with 3.8.1. In case a sample was associated with multiple sequencing runs, all pre-processed reads from the different sequencing runs were pooled prior to assembly. The RP15 pipeline as described by Castelle et al.^57^ was used to evaluate and confirm the phylogenetic diversity of MG-IV lineages present in the metagenome re-assemblies. The backbone of Zaremba–Niedzwiedzka et al.^46^ was updated to include Methanonatronarchaeia (SA1 archaea), Hadesarchaea, ArcI archaea, and MSBL1 archaea.

Per metagenome assembly, contigs larger than 2 kb were grouped into MAGs based on differential coverage across samples, tetranucleotide frequency profiles, GC-composition, and read-pair linkage. The contigs were first cut every 10 kb, unless the remaining fragment was shorter than 20 kb. Then the preprocessed reads of a set of sequencing runs (125_SRF_0.22-0.45 and 122_DCM_0.22-0.45: all sequencing runs listed in Supplementary Table 1; other samples: all sequencing runs highlighted in orange and blue in Supplementary Table 1) were mapped onto the fragmented contigs with kallisto v0.42.5^62^, yielding differential coverage profiles per fragmented contig. This was then used together with tetranucleotide frequency information by CONCOCT^63^ to group the fragmented contigs into bins. Bins corresponding to candidate MG-IV archaea were identified based on contigs phylogenetically classified as candidate MG-IV archaea by the RP15 pipeline and subsequently assessed and cleaned with mmgenome^64^ using differential coverage, GC-composition, read-pair linkage and presence of 162 genes well-conserved across Archaea. Finally, the fragmented contigs of the cleaned bins were replaced by their corresponding full length contigs to yield the final MAGs. In case not all fragmented contigs from a corresponding full length contig were present in a cleaned bin, the full length contig would only be included in the final bin if over half of the fragmented contigs was present.

### Completeness and redundancy estimation

Completeness and redundancy estimates of the MAGs were estimated with the miComplete tool^65^ (https://bitbucket.org/evolegiolab/micomplete). It assesses the presence and absence of 162 well-conserved marker genes among Archaea. The tool attempts to correct for co-localization bias of certain sets of genes (e.g., ribosomal protein genes) by assigning less weight to markers that typically co-localize with other markers and more weight to those that do not. Here, 8 markers not present in genomes of the closely related Haloarchaea were not included in the analysis to increase its accuracy.

### Annotation

The MAGs were annotated with prokka v1.12^66^, which was altered to allow for partial gene predictions on contig-edges (GitHub pull request #219), with the options --compliant, --partialgenes, --cdsrnaolap and --evalue 1e−10, and with barrnap as the rRNA predictor. Further information on gene annotations and functional predictions can be found in the Supplementary Text and in Supplementary Datas 6 and 7.

### Geographic distribution and phylogenetic diversity

All 16S rRNA gene amplicon surveys available in the sequence read archive were screened for reads of ≥400 bp that exhibited ≥93% sequence similarity relative to Hikarchaeia MAG 16S rRNA genes using the IMNGS^45^. GPS coordinates and sampling depths of all hits were extracted and used to plot the geographic distribution of Hikarchaeia (Supplementary Fig. 2). Hits were pooled with the 16S rRNA gene sequences from the Hikarchaeia MAGs and previously published Hikarchaeia 16S rRNA gene sequences^40^. Genetic redundancy of the dataset was reduced with VSEARCH (--cluster_fast --id 99)^67^. The dataset was supplemented with 36 representatives of Archaeoglobi, Methanonatronarchaeia, Methanomicrobiales, Methanocellales, Methanosarcinales and Haloarchaea and aligned with MAFFT L-INS-i v7.050b^68^. A ML phylogeny was inferred under the GTR + R6 model of evolution (selected by ModelFinder) with 100 nonparametric bootstraps^69^.

### Euryarchaeota species tree

A phylogenomics dataset was constructed consisting of 56 ribosomal proteins^46^ highly conserved among a representative set of 132 Euryarchaeota (including the five Hikarchaeia MAGs) and 10 TACK archaea (selected using phyloSkeleton^70^ v1.1.1 --best-match-only --completeness-threshold 0.6) (Supplementary Data 1). We manually excluded one taxon (*Halanaerobium sulfurireducens* M27-SA2), as we found that its inclusion destabilized the relative branching pattern of Haloarchaea in subsequent phylogenetic analyses (see “Data availability”). Each orthologous group was aligned with MAFFT E-INS-i^68^, and each alignment was trimmed with trimal -gappyout^71^ before concatenating all trimmed alignments into a supermatrix alignment. This “untreated” alignment, an SR4-recoded alignment^48^, a *χ*^2^-trimmed^47^ alignment where the 50% most heterogeneous sites were removed and an untreated alignment prepared without Haloarchaea and Hikarchaeia were then used for phylogenetic reconstruction under the CAT + GTR + Γ4 model as implemented in PhyloBayes-MPI v1.7a^72^ (Supplementary Figs. 5, 11, 13, and 15). Four independent MCMC chains were run until convergence was reached (maxdiff < 0.3) or a sufficient effective sample size was reached (effsize > 300), while using a burnin of 5000 cycles. Posterior predictive checks were performed with PhyloBayes-MPI to control whether the inferred phylogenetic models adequately captured the across-taxa compositional heterogeneity and site-specific pattern diversity present in the alignments. Parameter configurations were sampled every 50 generations after the burn-in. ML phylogenetic reconstructions were additionally done under the LG + C60 + F + Γ4 model (with 1000 ultra-fast bootstraps) and its PMSF approximation (with a 100 nonparametric bootstraps) for all non-recoded supermatrix alignments as implemented in IQTREE v1.5.3 or higher^69^ (Supplementary Figs. 4, 10, and 14). We used AU tests^73^ to evaluate three hypotheses describing the phylogenetic placements of Methanonatronarchaeia (MNA), Haloarchaea (HA), and Hikarchaeia (HIK) relative to other Methanotecta: MNA-basal, MNA + HA + HIK-basal and MNA + HA + HIK-within (Fig. 2, Supplementary Table 4). First, maximum likelihood trees were obtained with IQTREE under the constraint defined by each hypothesis (-g). We then added bootstrap trees (1000 ultra-fast bootstraps in case of LG + C60 + F + Γ4, 100 non-parametric bootstraps in case of its PMSF approximation) from the unconstrained maximum likelihood search to improve the accuracy of the AU test^74^. IQTREE was used to calculate site likelihoods (-wsl) for all (103 or 1003) considered trees under the LG + C60 + F + Γ4 or its PMSF approximation. The site likelihoods were fed to CONSEL v1.20^75^ to carry out the AU tests.

### rRNA gene phylogeny

16S and 23S rRNA gene sequences for all genomes used in the species tree (see above) were retrieved through rRNA gene prediction with barrnap 0.6 (github.com/tseemann/barrnap) (--kingdom arc, --evalue 1e-06). When one genome encoded multiple rRNA gene copies, the longest was chosen as a representative. 16S and 23S rRNA gene sets were aligned separately (alignment: MAFFT E-INS-i --adjustdirection, alignment trimmer: trimal -gappyout). Maximum likelihood (IQTREE v1.6.5 -m TESTNEW -mset GTR -b 100) and Bayesian inference (4 Phylobayes CAT + GTR + Γ4 MCMC chains) phylogenetic analyses were performed of the 16S rRNA gene alignment alone (selected model by ModelFinder: GTR + F + R6) and the concatenation of the 16S and 23S rRNA gene alignments (ModelFinder: GTR + F + R7).

### Amino acid composition analysis

Per-taxon amino acid frequencies were calculated with AMAS (summary -d aa -s)^76^ for the untreated supermatrix alignment and separately for the supermatrix alignment containing 50% least (the *χ*^2^-trimmed alignment) and 50% most heterogeneous sites (Supplementary Figs. 7 and 8). Principal component analysis biplots (Supplementary Fig. 6) were prepared for the untreated alignment. All plots were prepared with R using the ggplot2, ggfortify, reshape2, and ggbiplot packages (see drawCompositionPCA.sh and drawCompositionPCA.R in “Code availability”).

### Methanotecta species tree

The Euryarchaeota phylogenomics dataset was relieved of all non-Methanotecta and Methanonatronarchaeia sequences and subsequently supplemented with orthologs of twelve additional Haloarchaea with (near) complete genomes (Supplementary Data 1). A concatenated supermatrix alignment was prepared as described above and the *χ*^2^-trimmer was applied to remove the top 30% sites that contributed most to overall alignment heterogeneity. Bayesian (CAT + LG + Γ4) and ML (LG + C60 + F + Γ4 or its PMSF approximation) tree inferences were carried out as described above.

### Gene clusters and gene trees

For the 57 taxa included in the Methanotecta species tree, complete proteomes were functionally annotated with eggNOG-mapper v1.0.3^77^ using the eurNOG (Euryarchaeota specific orthologous groups) database (eggNOG v.4.5.1^53^). Based on this annotation, 129,270 out of the total 158,269 proteins were grouped into 10,423 clusters corresponding to eurNOGs. Using the eggNOG database to infer clusters has several advantages that are exploited in this work: (i) straightforward gene family annotation, (ii) hierarchical links to COGs (see “Origins of gene families acquired by Haloarchaea” below), (iii) availability of precomputed alignments, HMM profiles and gene trees, and (iv) readily accessible domain architectures. All proteins that could not be assigned to any eurNOG were subjected to an all-vs.-all BLAST search and SiLiX v1.2.11^78^ (--ident 0.6 --overlap 0.9) de novo clustering strategy, resulting in an additional 28,999 clusters. Unlike the eggNOG database, which aims to provide clusters of orthologous genes at different taxonomic levels, the SiLiX algorithm aims to reconstruct homologous gene families (without a taxonomic scope). We aimed to reconcile these two approaches by choosing rather conserved thresholds for the SiLiX clustering in order to avoid clustering out-paralogs into the same group. This approach resulted, as expected with these parameters, in small gene families, with only three containing more than ten sequences and most representing singleton clusters.

An assumption of ALE is that all residues of a protein share the same phylogenetic history. However, it has been suggested that Haloarchaea encode relatively large numbers of composite/chimeric proteins where different regions (or components) of the protein are the result of (partly) independent phylogenetic histories^79^. We identified 307 clusters with 7776 putative fusion proteins that based on the eggNOG mapper annotations were associated with two or more COG/NOG clusters. Whereas the eggNOG database assigns full fusion proteins to two or more COGs/NOGs (i.e., the full fusion protein sequence is member of all COGs/NOGs it is associated with), the NCBI COG database assigns the components of a protein to its corresponding, separate COGs (i.e., the fusion protein sequence is split into its constituent COG components and each component is member only of its corresponding COG). We exploited this property and screened all proteins of the putative fusion families by querying them with HMM profiles (HMMER 3.2.1^80^) generated from the aligned sequences (MAFFT FFT-NS-i) of the NCBI COGs^81^. Protein components homologous to specific COGs identified through this HMM search were then separated from the rest of the protein and placed in new gene family. In effect, any gene family that harbored at least one fusion protein would be split into two or more (depending on the number of unique COGs found) new gene families. For example, the gene family 0KS6Y was split into 0KS6Y_COG1009 and 0KS6Y_COG2111. When a fusion protein contained multiple regions associated with the same COG, each region would constitute a separate sequence in the new gene family (this in principle allows one to study the domain duplication history in the gene family). When two regions associated with different COGs overlapped in the same protein, the better scoring region (score based on HMM alignment) was preferred, and the lower scoring region was trimmed accordingly. If after trimming the remaining length of the region was less than 30% of the original length of the region, the region was discarded from the analysis. The splitting procedure resulted in 626 clusters with 12,857 proteins or protein domains.

From the total of 37,674 clusters with 163,350 proteins or distinct domains, those consisting of at least four sequences were subjected to phylogenetic reconstruction. A prefiltering approach was applied to these 7375 clusters in order to remove non-homologous residues (PREQUAL v.1.02^82^) prior to reconstructing multiple sequence alignments with MAFFT E-INS-i v7.050b^68^. Gene trees were reconstructed for each alignment using IQTREE v1.6.9 with 1000 ultra-fast bootstraps and the -bnni option for further bootstrap refinement^83^. The substitution model was chosen between LG + Γ with C10 to C60 profile mixture models^84^ based on the BIC criterion in IQTREE’s ModelFinder^85^. For clusters with two or three members, “bootstrap” samples were generated rather than reconstructed, as there is only a single unrooted topology in these cases.

### Gene tree aware ancestral reconstruction

Using ALE v0.4^49^, conditional clade probabilities^86^ were computed from the bootstrap samples (ALEobserve) and 100 reconciliations with the species tree were sampled (ALEml_undated)^50^. We adjusted the reconstructed genome copy number with the extinction probability per cluster within ALE (see github.com/maxemil/ALE/commit/136b78e). Singleton clusters were counted as originations at the corresponding species node.

We applied a threshold of 0.3 to the raw reconciliation frequencies that are the output of ALE, meaning that events (loss, transfer, origination or duplication) and presence/absence (copies) were counted as such if they had a frequency of at least 0.3. This rather relaxed threshold means that the results are quite sensitive, i.e., even low frequency events are detected. This is necessary as the amount of “noise” from the alignment and the tree reconstruction is rather high and we expect that many true events would be missed otherwise. A summary of the copy number (genome size) and the events were then visualized along the branches of the species tree.

### Origins of gene families acquired by Haloarchaea

We first identified all gene families that had an inferred origination event (origination frequency ≥ 0.3) at the LHHCA or any of its descendants. We inferred the possible taxonomic source of the origination event (in case the origination event corresponded to an incoming transfer from outside the species tree) or taxonomic target (in case the event corresponded to a gene birth followed by an outgoing transfer) by placing all family sequences onto reference NOG level eggNOG phylogenies using a phylogenetic placement algorithm (see below). Briefly, a phylogenetic placement algorithm attaches a novel sequence to all possible branches in a reference tree (while keeping the tree and its corresponding ML parameters fixed) and picks the placement that yields the highest likelihood. We chose to use the eggNOG 4.5.1 database because the pre-defined gene families as well as accompanying pre-calculated gene family alignments and trees save a vast amount of computational resources. Though the eggNOG database is not as exhaustive as e.g., the NCBI-NR database, with 2031 representative genomes across the tree of life its approximation of total sequenced phylogenetic diversity is adequate for this analysis. To avoid self-placement, any Methanotecta representative in the NOG was removed from the reference tree and reference alignment prior to placement. In case the remaining NOG tree/alignment had less than four taxa, the placement algorithm could not be run (it requires at least four taxa) and we considered the sequence “placed” at the last common ancestor of the remaining NOG taxa. In rare cases where no reference NOG trees were available from the eggNOG database, no placement analysis was performed. Gene family sequences were first aligned with the reference NOG alignment with MAFFT –addfragment --keeplength v7.407^68^ and subsequently placed onto the reference tree using the Evolutionary Placement Algorithm (EPA-ng v0.3.4^87^), with substitution models evaluated under LG + F + I using raxml-ng^88^. All placements of a gene family were summarized with gappa v0.4.0^89^, and the taxonomic label with the highest relative likelihood weight was picked as the phylogenetic affiliation of the origination event. To test the robustness of the placement we compared the placement labels to the labels of the sister branches of the Methanotecta sequences present in the raw reference trees.

### Reporting summary

Further information on research design is available in the Nature Research Reporting Summary linked to this article.

## Supplementary information

Supplementary Information

Reporting Summary

Description of Additional Supplementary Files

Supplementary Data 1

Supplementary Data 2

Supplementary Data 3

Supplementary Data 4

Supplementary Data 5

Supplementary Data 6

Supplementary Data 7

Supplementary Data 8

## Data Availability

The genome bins described in this study have been deposited at DDBJ/EMBL/GenBank under the BioProject ID PRJNA588249. The metagenome assemblies (10.6084/m9.figshare.11117894.v1), phylogenomics dataset and supermatrix alignments (10.6084/m9.figshare.11118164.v1), species trees that include *Halanaeroarchaeum sulfurireducens* M27-SA2 (10.6084/m9.figshare.12964832.v3), and unaligned sequences for gene families with less than four members, ultra-fast bootstrap trees for gene families with at least four members and ALE inferred gene family histories (10.6084/m9.figshare.11352140) are available on FigShare.

## Code availability

All relevant scripts and command-line parameters are available on github.com/maxemil/haloarchaea-evolution.
