# Supplementary material for: Hikarchaeia demonstrate an intermediate stage in the methanogen-to-halophile transition
Source: Nat Commun. 2020 Oct 30;11:5490. doi: 10.1038/s41467-020-19200-2 (PMC7599335; doi:10.1038/s41467-020-19200-2)
Supplement: Supplementary file 1 — Supplementary Information [file 41467_2020_19200_MOESM1_ESM.pdf]

Supplementary Information
for
Hikarchaeia demonstrate an intermediate stage in the methanogen-to-halophile transition

Joran Martijn*, Max E. Schön*, Anders E. Lind, Julian Vosseberg,
Tom A. Williams, Anja Spang, Thijs J. G. Ettema

Contents

1 Supplementary Discussion **4**
1.1 Inferred metabolism of the Hikarchaeia 4
1.2 How many gene families of bacterial origin were acquired via horizontal gene transfer during the methanogen-to-halophile transition? 6

2 Supplementary Methods **10**

References **12**

List of Tables

1 Supplementary Table 1 16
2 Supplementary Table 2 17
3 Supplementary Table 3 18
4 Supplementary Table 4 19
5 Supplementary Table 5 20

List of Figures

1 Archaeal phylogenetic diversity in selected samples. 21
2 Metabolic map of Hikarchaeia. 22
3 Geographic distribution of Hikarchaea 16S rRNA gene sequences. 23
4 16S rRNA gene maximum likelihood phylogeny depicting Hikarchaea sampled diversity. 24

5 Maximum likelihood phylogeny of Euryarchaeota based on the untreated concatenated alignment of 56 ribosomal proteins. 26

6 Bayesian inference of Euryarchaeota phylogeny based on the untreated concatenated alignment of 56 ribosomal proteins. 30

7 Bayesian inference of Euryarchaeota phylogeny based on the χ^2-trimmed (50% most heterogeneous sites removed) concatenated alignment of 56 ribosomal proteins. 34

8 Bayesian inference of Euryarchaeota phylogeny based on the SR4-recoded concatenated alignment of 56 ribosomal proteins. 38

9 Maximum likelihood phylogeny of Euryarchaeota based on the χ^2-trimmed (50% most heterogeneous sites removed) concatenated alignment of 56 ribosomal proteins. 40

10 16S rRNA gene maximum likelihood phylogeny. 41

11 16S rRNA gene Bayesian phylogeny. 42

12 16S+23S rRNA gene maximum likelihood phylogeny. 43

13 16S+23S rRNA gene Bayesian phylogeny. 44

14 Principal component analysis of amino acid compositions of the untreated 56 ribosomal protein supermatrix alignment. 47

15 Amino acid compositions of the untreated 56 ribosomal protein supermatrix alignment. 48

16 Comparison of amino acid compositions of the 50% most heterogeneous sites versus the 50% least heterogeneous sites (χ^2-trimmed) of the 56 ribosomal protein supermatrix alignment. 49

17 Removal of most compositionally heterogeneous sites. 50

18 Maximum likelihood phylogeny of Euryarchaeota based on the concatenated alignment of 56 ribosomal proteins excluding Haloarchaea and Hikarchaea. 52

19 Bayesian inference of Euryarchaeota phylogeny based on the concatenated alignment of 56 ribosomal proteins excluding Haloarchaea and Hikarcheae. 56

20 Removal of most compositionally heterogeneous sites for the Methanotecta dataset with extended Haloarchaea sampling. 57

21 Maximum likelihood phylogeny of Methanotecta based on the χ^2-trimmed (30% most heterogeneous sites removed) concatenated alignment of 56 ribosomal proteins with extended Haloarchaea sampling. 59

22 Bayesian inference of Methanotecta phylogeny based on the χ^2-trimmed (30% most heterogeneous sites removed) concatenated alignment of 56 ribosomal proteins with extended Haloarchaea sampling. 60

23 Maximum likelihood phylogeny of Methanotecta based on the untreated concatenated alignment of 56 ribosomal proteins with extended Haloarchaea sampling. . 62

24 Bayesian inference of Methanotecta phylogeny based on the untreated concatenated alignment of 56 ribosomal proteins with extended Haloarchaea sampling. . 63

25 Bayesian inference of Methanotecta phylogeny based on the SR4-recoded concatenated alignment of 56 ribosomal proteins with extended Haloarchaea sampling. 64

26 Frequency of incoming transfers for main groups of the species tree. 65

27 Detailed gene flow diagrams for gene families related to methanogenesis and respiration. 66

28 Detailed gene flow diagrams for gene families related to salt adaptation and UV resistance. 67

29 Tracing the origins of gene acquisitions. 68

30 Reference family size distribution for different taxonomic affiliations. 69

1 Supplementary Discussion

1.1 Inferred metabolism of the Hikarchaeia

Electron transport chain. In spite of having small genomes, Hikarchaeia share various features of their energy metabolism with Haloarchaea. First of all, members of this group encode an aerobic electron transport chain composed of an NADH dehydrogenase (Complex I), a cytochrome bc1 complex (Complex III), a heme/copper-type cytochrome/quinol oxidase (Complex IV) and an ATP synthase (Complex V). Similar to other aerobic Archaea including Haloarchaea, Thaumarchaeota and Crenarchaeota (She et al 2001; Gonzalez et al 2009; Spang et al 2013), Complex I of Hikarchaeia lacks the nuoEFG module suggesting that electrons are accepted from a donor molecule other than NADH, while the latter may instead be oxidized by an unrelated type II NADH dehydrogenase (InterPro domains IPR023753, IPR036188) (Melo et al 2004). In contrast to Haloarchaea, Hikarchaeia do not seem to possess a membrane-bound Complex II as they only encode a putative soluble succinate dehydrogenase/fumarate reductase, which can operate in the TCA but cannot contribute to the generation of membrane potential (see below). Finally, the genomes of Hikarchaeia do not show any clear indication for alternative terminal acceptors, which is consistent with the observation that members of this group seem to be restricted to oxygenated marine waters (Supplementary Fig. 3).

Carbon metabolism. Hikarchaeia encode the Embden-Meyerhoff pathway (glycolysis) but, similar to Haloarchaea, seem to lack key gluconeogenic enzyme bifunctional fructose-1,6-bisphosphate aldolase/phosphatase (Fbp; arCOG family arCOG04180) present in many Archaea (Say and Fuchs, 2010). Furthermore, they harbor a complete tricarboxylic acid cycle (TCA), with a soluble two-subunit succinate dehydrogenase/fumarate reductase, which seems to be most closely related to bacterial homologs rather than to the corresponding subunits of Complex II of Haloarchaea (Supplementary Data 6). Furthermore, while members of this group appear to lack key enzymes of the ribulose-5-phosphate pathway (i.e., lack of D-arabinose 5-phosphate isomerase and 3-keto-L-gulonate-6-phosphate decarboxylase) and nonoxidative pentose phosphate pathway (lack of transaldolase and transketolase), they may use the oxidative pentose phosphate pathway for the synthesis of the key metabolic intermediate ribose-5-phosphate (Supplementary Data 6 and 7) (Bräsen, 2014). Finally, the Hikarchaeia seem to comprise heterotrophic organisms as they do not encode key enzymes for any of the characterized carbon fixation pathways (Fuchs, 2010).

Potential growth substrates. Notably, all Hikarchaeia have several paralogs (Supplementary Data 6) of the three subunits (CoxS, M and L) of putative xanthine/CO dehydrogenases (CODH-like enzymes), which is interesting considering that several Haloarchaea have been shown to be able to use carbon monoxide (CO) as growth substrate (e.g. King 2018). To determine whether the Hikarchaeia homologs could indeed comprise bona fide CODH (King and Weber 2007, Cordero et al 2019), we investigated the presence of accessory genes (Santiago et al 1999), performed phylogenetic analyses of the largest subunit of the CODH-like enzymes, i.e. CoxL (`https://doi.org/10.6084/m9.figshare.12629882`) and investigated se-

quence motifs characteristic for Form I and II CODH. While the Hikarchaeia encode candidate genes for the accessory proteins CoxD, E, F, G, I and K (similar to Silicobacter pomeroyi (Moran 2014)) (Supplementary Data 6, 7 and Supplementary Table 5), their cox genes do not occur in gene clusters or operons as commonly observed in CO oxidizers (King 2018). Furthermore, phylogenetic analyses (https://doi.org/10.6084/m9.figshare.12629882) reveal that the hikarchaeial CoxL homologs, while comprising CODH-like enzymes, form several separate clades, none of which clusters with bona fide archaeal and bacterial CODH (https://doi.org/10.6084/m9.figshare.12629882). Yet, one of the hikarchaeial CoxL clades comprises two subclades each with sequences that have conserved active site residues similar to Form II CODH (Supplementary Data 6, 7 and Supplementary Table 5) (King and Weber 2007). On the other hand, none of the three described structural motifs of Form I and II CODH seem to be completely conserved. In turn, experimental approaches will be needed to test whether the CoxL homologs with active site residues of Form II CODH could belong to enzymes acting on CO. Taken together, this suggests that most (to all) of the hikarchaeial CODH-like enzymes do not oxidize CO and instead mediate other metabolic processes (see below).

In proximity to *cox*SML genes, Hikarchaeia encode a putative exported esterase (presence of a signal peptide as assessed using PRED-SIGNAL) between 37 and 41% similar to cocaine esterases of Rhodococcus sp., in which these proteins cleave the benzoylester cocaine to ergonine methyl ester and benzoate (Bagos 2009, Bresler 2000). It therefore seems possible that homologs of this protein family in Hikarchaeia could act on certain benzoyl esters present in marine waters and produce the aromatic acid benzoate (Supplementary Fig. 2). In this regard, it is worth mentioning that Haloarchaea are known to use aromatic acids such as benzoate and hydroxybenzoate as sole carbon and energy source during aerobic growth (Fairley 2002; Cuadros-Orellana 2012; Dalvi 2016). While the catabolic pathways for the degradation of the various aromatic acids in Haloarchaea are not completely elucidated, gentisate and protocatechuic acid have been suggested to represent potential intermediates (Fariley 2002; Dalvi 2016). To further determine whether Hikarchaeia may use aromatic compounds (e.g. benzoate) as substrates, we investigated the presence of protein homologs (based on Pfam domains), that are involved in aromatic acid degradation in Bacteria and Haloarchaea (Figure 4, Supplementary Data 3). Interestingly, we identified candidate proteins for a variety of reactions that may enable the degradation of benzoate, phenol, mandelate or similar compounds. Notably, some of the CoxSML paralogs (such as 4-hydroxybenzoyl-CoA reductase) encoded by Hikarchaeia may in fact be involved in aromatic acid degradation by catalyzing reactions such as 4-hydroxybenzoyl-CoA to benzoyl-CoA and/or benzaldehyde to benzoate (Supplementary Fig. 2), which are mediated by CODH-like enzymes (i.e. molybdenum cofactor-containing enzymes).

Thus, it is tempting to speculate that Hikarchaeia can use aromatics and benzoyl-esters as growth substrates. Furthermore, the presence of genes for candidate enzymes for beta-oxidation as well as Etf, may indicate that Hikarchaeia could also use fatty acids as carbon and energy source. While prospective experimental work will be necessary to test these predictions and better characterize metabolic pathways for aromatic acid degradation in archaea, our work may help designing media selectively enriching for members of this novel archaeal lineage and determine their ecological role in the degradation of potentially toxic compounds in marine waters.

Note: CoxL homologs with partial active site are located next to MIP family channel proteins which comprise aquaporins and glycerol facilitators and could for instance export CO_2 (https://www.ebi.ac.uk/interpro/entry/InterPro/IPR023271/).

Anabolism. Similar to aerobic marine Thaumarchaeota (MG-I) but in contrast to marine Poseidoniia (MG- II/III) (Villanueva et al 2016. Rinke et al 2018), Hikarchaeia encode all archaeal lipid biosynthesis proteins including the hallmark protein glycerol-1P-dehydrogenase. Furthermore, the Hikarchaeia seem to encode complete pathways for the de novo synthesis of all amino acids (as are tRNA synthetases) except for proline and cysteine; however, the central enzymes ProC, CysK and CysE are present for the latter. Furthermore, the pyrimidine synthesis pathway is complete, whereas the initial part of the purine synthesis pathway is incomplete (i.e. from PRPP to IMP). However, the Hikarchaeia may use a salvage pathway for producing IMP (enzymes Apt, PurH, PurO), The pathway from IMP onwards is complete.

Conclusion. Altogether, our analyses indicate that Hikarchaeia comprise aerobic members, which likely use different organic substrates for organoheterotrophic growth. It is currently unclear whether members of this group could also grow lithoheterotrophically by using inorganic substrates such as carbon monoxide as electron donors. Thus, while Hikarchaeia share a similar mode of metabolism with Haloarchaea and differ from their anaerobic methanogenic relatives, not all aspects of their electron chains are conserved with Haloarchaea. For example, Hikarchaeia lack certain components such as Complex II, suggesting that the respective genes were either acquired later by Haloarchaea or were lost in the Hikarchaeia as a result of genome streamlining processes common in organisms adapting to oligotrophic marine environments (Giovannoni, 2014).

1.2 How many gene families of bacterial origin were acquired via horizontal gene transfer during the methanogen-to-halophile transition?

The gene content and metabolism of Haloarchaea are quite distinct from their methanogen relatives, and several published analyses (Nelson-Sathi et al 2012; Nelson-Sathi et al 2015; Becker et al 2014; Groussin et al 2016; Méheust et al 2018) have investigated when and from where the genes underpinning this distinct metabolism were acquired. While most analyses agree that haloarchaeal genomes encode many genes of bacterial origin, both the dataset and the gene mapping approach used have had a major impact on inferences of the total number of gene acquisitions and the time(s) in haloarchaeal history at which they were acquired. Our work differs from previous analyses in two main respects: the inclusion of the newly sampled Hikarchaeia, which break the long branch leading to Haloarchaea, and the use of the gene tree-species tree reconciliation algorithm ALE (Szollosi 2013) for estimating the branch on which each gene family originated. A previous study used ALE to map archaeal gene family evolution (Williams et al. 2017) but the analysis did not focus on Haloarchaea and taxon sampling for addressing this question was very limited.

The question of haloarchaeal gene origins has been of interest since Nelson-Sathi et al (Nelson-Sathi et al 2012) suggested that the evolutionary transformation of an anaerobic methanogen

into an aerobic, salt-loving heterotroph was the result of a single large-scale transfer of 1089 genes from an ancestral bacterium into the last Haloarchaea common ancestor (LHaCA). Briefly explained, they clustered all proteins encoded by 75 complete archaeal genomes (including 10 Haloarchaea) into 16061 gene families (not counting singletons). A similarity search for their homologs in 1078 complete bacterial genomes subsequently identified 1479 families that had bacterial homologs as well as a minimum of two haloarchaeal homologs. After constructing maximum likelihood trees, 1089 gene families were identified in which all haloarchaeal homologs formed a monophyletic group that was sister to a bacterial monophyletic group. It was concluded that such topologies were best explained by bacteria-to-haloarchaea transfers. To evaluate whether these 1089 transfers occurred at the LCA of the Haloarchaea or during their divergence, the authors compared the distribution of phylogenetic splits of the 1089 transferred genes with those of 364 genes unique to archaea and that are highly conserved in Haloarchaea. These 'core' genes were thought to be present in the LHaCA. The rationale for this comparison was that if the phylogenetic split distribution of the core and transferred genes was statistically indistinguishable, the null hypothesis that the horizontally acquired genes were also present in the LHaCA could not be rejected. In a follow up study, a similar approach was used to study the role of bacterial transfer in the evolutionary emergence of thirteen major archaeal clades, including the Haloarchaea (Nelson Sathi et al-2015). Now with a dataset that included 22 complete Haloarchaea genomes, the authors inferred 1047 bacterial gene transfers at the LHaCA, an estimate that corresponded well with their initial study.

The findings of Nelson-Sathi et al. have subsequently been critiqued in the literature. Becker et al (Becker et al 2014) applied the same alignment- and tree inference methods as Nelson Sathi et al 2012 to the same dataset of 1479 gene families but found only 656 instead of 1089 trees in which all haloarchaeal homologs formed a monophyletic group with a bacterial sister group. In particular, when the authors expanded the 656 gene families with their homologs from 65 additional Haloarchaea genomes, the haloarchaeal sequences were monophyletic in only 178 families (note that Becker et al. only re-analyzed 443 of the original families due to issues with dataset size). Becker et al thus inferred that the origin of Haloarchaea was associated with a minimum of 178 and a maximum of 291 incoming transfers from bacteria. The large majority of the 1479 families with haloarchaeal and bacterial homologs were linked with non-monophyletic Haloarchaea. The authors interpreted such topologies as support for multiple independent bacteria-to-Haloarchaea transfers in particular haloarchaeal lineages, rather than in the LHaCA. Their analysis highlights the importance of having a taxon sample that adequately represents haloarchaeal diversity and suggest that lineage specific acquisitions might appear to map to the origin of Haloarchaea when taxon sampling is limited.

Groussin et al (Groussin et al 2016) criticised the gene mapping approach used by Nelson-Sathi et al. (2012). They argued that by inferring a transfer from an ancestral bacterium into the LHaCA, one must not only count the gene gain, but also count the gene loss in all the Haloarchaea- and bacterial descendant lineages that lack the gene. When most Haloarchaea and bacterial descendants are represented in the gene family, the number of inferred losses is limited, and the scenario is plausible. However, when only very few Haloarchaea and/or bacteria are represented, the number of inferred losses would become unrealistically high. In such cases it would be more parsimonious to infer an origin in a more recent lineage, followed

by one or more transfers to other recent represented lineages (see Groussin et al 2016 Figure 1). An additional critique (Groussin et al. 2016) is that both the placement of the origin and the direction of the transfer are unclear. Thus, under the method of Nelson-Sathi et al, small gene families with few haloarchaea and bacteria may be erroneously associated with LHaCA acquisitions. This effect might have had a substantial impact on the analysis, as half of the gene families investigated by Nelson-Sathi et al (2015) had fewer than three archaea or fewer than 21 bacteria (out of 1847 bacteria in the dataset). The authors further argue that the result of the statistical test used by Nelson-Sathi et al to compare phylogenetic splits of 'transferred' and 'core' genes implies that both sets have similar presence-absence profiles and transfer rates but is not informative on the question of whether each set was predominantly acquired at the base of major archaeal clades. To address some of these issues, Groussin et al opted to re-analyze the NS-2015 data with COUNT (Csuros 2010), an ancestral gene repertoire reconstruction method that allows for lineage specific gene gains and losses given a probabilistic birth-death model and a reference species tree. With this method, only an expected total number of 215 gene acquisitions was inferred at the base of the Haloarchaea. However, COUNT models presence-absence profiles rather than gene tree topologies, ignoring a useful source of information about gene family history, and does not explicitly model gene transfers. Published analyses also suggest that COUNT fails to recover the major gene gain associated with the plastid endosymbiosis in eukaryotes, a case in which there is strong biological evidence in support of a major ancestral gain followed by differential gene loss (Kapust et al 2018 GBE).

In the present work we revisit the question with an independently constructed dataset of gene families and a gene tree aware ancestral reconstruction method. To compare our results with that of the studies described above, one must understand the key differences in dataset construction and methods used.

Considering dataset construction, Nelson-Sathi et al built their gene families by clustering proteins from a phylogenetically diverse set of archaeal genomes and subsequently expanded these with homologs of a large phylogenetically diverse set of bacterial genomes. Here, we focused specifically on Methanotecta and built gene families by assigning all their proteins to Euryarchaeota-level eggNOGs. This has an important consequence: one 'archaeal' gene family could correspond to two or more 'euryarchaeotal' gene families. Thus, our dataset contains a larger number of gene families with Haloarchaea representatives compared to the dataset of the above discussed studies. This in turn means that we cannot directly compare the inferred number of bacterial acquisitions along the methanogen-to-halophile transition between our study and that of the above studies.

Considering the ancestral reconstruction method used, Nelson-Sathi et al and Becker et al recognized transfers of bacterial origin as gene trees in which all Haloarchaea representatives formed a monophyletic group relative to a bacterial sister lineage. However, this strategy places bacterial acquisitions at the LHaCA even when only few Haloarchaea are represented in the gene family. Groussin et al attempted to overcome this issue by using COUNT to account for post-acquisition, lineage-specific gene losses probabilistically. Here, bacterial transfers appear as gains at the LHaCA. In this work, we attempt to overcome this issue by employing ALE, an ancestral reconstruction method that uses the information from gene tree topologies. An important detail of our chosen approach is that the scope of the ancestral reconstruction analysis is limited

to the Methanotecta (including the entire tree of life is computationally and phylogenetically infeasible). As a result, we cannot observe bacterial transfers along the methanogen-to-halophile transition directly as Nelson-Sathi et al and Becker et al did. Instead, such transfers appear as 'originations' at the LHHCA and LHaCA. An origination refers to the appearance of a new gene in some ancestral node in the species tree and can correspond biologically to a bona fide gene birth or an incoming transfer from outside the species tree. To determine the nature (gene birth or transfer) and phylogenetic affiliation of an LHHCA/LHaCA origination, we placed the sequences of the originated gene family onto eggNOG reference trees that included homologs across the tree of life. This approach enabled us to establish the bacterial affinity of horizontally acquired genes.

A key difference in our analysis compared to those of previous works is the inclusion of genomes from the Hikarchaeia (previously 'Marine Group IV'). Including this lineage breaks the branch that separates the Haloarchaea from the methanogenic ancestor, enabling us to distinguish the order in which some of the key bacterial-origin genes were acquired during the methanogen-to-halophile transition.

We identified a total of 661 at least weakly supported originations at the LHHCA-LHaCA branch and 158 at the LMHHCA-LHHCA branch (Figure 3). From these, 194 and 43 were phylogenetically affiliated with bacteria as inferred by phylogenetic placement analysis, respectively. However, these 237 could still include a number of false positives (i.e., genes that appear as bacterial transfers along the methanogen-to-halophile transition but are not) and exclude a number of false negatives (i.e., genes that do correspond to bacterial] transfers along the transition but were missed). The root cause for these false inferences is carrying out the gene tree aware ancestral reconstruction without including all complete genomes across the tree of life. For example, because our gene families did not include all (or at least a highly representative set of) bacterial genomes, we cannot infer in which of the bacteria-affiliated families the bacterial lineage was the donor, and in which it was the recipient. In addition, our gene families did not include all currently available (near) complete Haloarchaea genomes. While we did strive to represent as much as known genomic diversity as possible, we purposefully kept the number of Haloarchaea genomes to a minimum to prevent phylogenetic artefacts in the species- and gene trees induced by a combination of unbalanced taxon sampling (the trees would include a very large number of Haloarchaea relative to a small number of methanogens) and compositional biases (Haloarchaea have uniquely acidic amino acid compositions to survive in extreme salt concentrations). If some of the 194 happen to be well represented in our set of 24 Haloarchaea but are poorly represented in all other sequenced Haloarchaea genomes, and their phylogenetic signal may by chance be congruent with an LHaCA origin, they will falsely appear as acquisitions at the LHHCA-LHaCA branch. Finally, if a gene truly was acquired from bacteria along the methanogen-to-halophile transition, but also had a deeper ancestry within Methanotecta and was represented in at least some of the sampled methanogen genomes, it would not appear as an origination at the LHHCA or LHaCA, but rather as an origination in an older Methanotecta ancestor followed by vertical inheritance to the LHHCA/LHaCA or a transfer from a methanogenic lineage to the LHHCA/LHaCA.

In summary, the number of bacterial genes that was acquired during the methanogen-to-halophile transition was initially inferred to be over a thousand. As this set included most of the

genes functionally necessary for a heterotrophic and aerobic lifestyle, it gave rise to the exciting hypothesis that all genes were acquired from an ancestral bacterial donor in a single large-scale transfer. However, as follow up studies have incorporated more representative sets of Haloarchaea in their datasets and employed more sophisticated ancestral reconstruction methods that account for increasingly more facets of gene family evolution, this number has reduced. Our work added Hikarchaeia to the analysis and inferred a total of 237 bacterial gene families that were acquired over at least two stages along the methanogen-to-halophile transition. In addition, our results support the scenario that genes of bacterial origin have shaped haloarchaeal genomes both at their origin and during their diversification. Finally, it is important to realize that none of the currently available methods are consummate (account for all facets of gene family evolution) and this estimate is expected to change as genome sampling and ancestral reconstructions improve.

2 Supplementary Methods

CoxL phylogeny including Hikarchaeia paralogs with the Pfam domain 'PF01315'. Hikarchaeia sequences with the PF01315 domain were extracted from the proteomes and added to a reference set of highly curated CoxL homologs (Cordero et al, 2019) encoded by bona fide carbon monoxide oxidizing archaea and bacteria. The combined sequences (n=746) were aligned using MAFFT L-INS-i v7.407 (default options; Katoh and Standley 2013) and trimmed using BMGE v1.12 (-m BLOSUM30 -b 2 -h 0.55; Criscuolo and Gribaldo 2010) yielding 775 aligned positions. Subsequently, a maximum likelihood phylogeny was inferred using IQ-TREE v1.6.7 (Nguyen et al 2015) with the mixture model LG+C20+R4+F. Branch support was assessed using ultrafast bootstrap estimates (Hoang et al 2018) and SH-like approximate likelihood ratio test (each 1000 replicates).

Extended PF01315 domain phylogeny. Archaeal and bacterial sequences with the PF01315 domain were downloaded from UniProt and downsampled by keeping only those sequences with a length between 400 and 1200 amino acids. This set was further reduced using CD-HIT v4.7 with a sequence identity threshold of 0.70. Subsequently, this set was combined with the bona fide CoxL homologs (Cordero et al., 2019) and the hikarchaeial PF01315 domain proteins (see above) yielding 8551 sequences. These were aligned using MAFFT (–globalpair) and trimmed using BMGE v1.12 (-m BLOSUM30 -b 2 -h 0.55) yielding 288 aligned positions. An IQ-TREE was inferred using the LG+C10 model.

Protein annotation. All proteins from the 5 Hikarchaeia MAGs were annotated using several databases and programs. We used the existing annotations provided by prokka (Seemann 2014) and the eggNOG-mapper v1.0.3 (from which we also gathered the KEGG KO numbers with corresponding annotations and pathways as well as the arCOGs; Huerta-Cepas et al 2017, Kanehisa and Goto 2000, Makarova et al 2015). Additionally, we annotated the proteins using the Carbohydrate-Active enZYmes Database (CAZY, using HMMER; Cantarel et al 2009, Eddy 2011), the Transporter Classification Database (TCDB, using BlastP; Saier et al 2006) and used

InterProScan to annotate the proteins with PFAM, TIGRFAM and IPR domains (Jones et al 2014, El-Gebali et al 2019, Haft et al 2001). Finally, we used DIAMOND (Buchfink et al 2015) to perform similarity searches of the proteins against NR and recorded the taxonomic annotation of the last common ancestor of all hits within 2 percent of the best score. All annotations are presented in Supplementary Data 7.

References

P. G. Bagos, K. D. Tsirigos, S. K. Plessas, T. D. Liakopoulos, and S. J. Hamodrakas. Prediction of signal peptides in archaea. *Protein Engineering, Design and Selection*, 2009.

Erin A. Becker, Phillip M. Seitzer, Andrew Tritt, David Larsen, Megan Krusor, Andrew I. Yao, Dongying Wu, Dominique Madern, Jonathan A. Eisen, Aaron E. Darling, and Marc T. Facciotti. Phylogenetically Driven Sequencing of Extremely Halophilic Archaea Reveals Strategies for Static and Dynamic Osmo-response. *PLoS Genetics*, 10, 2014.

Christopher Bräsen, Dominik Esser, Bernadette Rauch, and Bettina Siebers. Carbohydrate metabolism in archaea: Current insights into unusual enzymes and pathways and their regulation. *JAMA Ophthalmology*, 132, 2014.

Matthew M. Bresler, Susan J. Rosser, Amrik Basran, and Neil C. Bruce. Gene cloning and nucleotide sequencing and properties of a cocaine esterase from Rhodococcus sp. strain MB1. *Applied and Environmental Microbiology*, 66, 2000.

Benjamin Buchfink, Chao Xie, and Daniel H Huson. Fast and sensitive protein alignment using DIAMOND. *Nature methods*, 12, 2015.

Brandi I. Cantarel, Pedro M. Coutinho, Corinne Rancurel, Thomas Bernard, Vincent Lombard, and Bernard Henrissat. The Carbohydrate-Active EnZymes database (CAZy): An expert resource for glycogenomics. *Nucleic Acids Research*, 2009.

Paul R.F. Cordero, Katherine Bayly, Pok Man Leung, Cheng Huang, Zahra F. Islam, Ralf B. Schittenhelm, Gary M. King, and Chris Greening. Atmospheric carbon monoxide oxidation is a widespread mechanism supporting microbial survival. *ISME Journal*, 13, 2019.

Alexis Criscuolo and Simonetta Gribaldo. BMGE (Block Mapping and Gathering with Entropy): A new software for selection of phylogenetic informative regions from multiple sequence alignments. *BMC Evolutionary Biology*, 10, 2010.

Miklós Csurös. Count: Evolutionary analysis of phylogenetic profiles with parsimony and likelihood. *Bioinformatics*, 26, 2010.

Sara Cuadros-Orellana, Metchild Pohlschroder, Matthew J. Grossman, and Lucia R. Durrant. Biodegradation of aromatic compounds by a halophilic archaeon isolated from the Dead Sea. *Chemical Engineering Transactions*, 27, 2012.

Sonal Dalvi, Noha H. Youssef, and Babu Z. Fathepure. Microbial community structure analysis of a benzoate-degrading halophilic archaeal enrichment. *Extremophiles*, 20, 2016.

Sean R. Eddy. Accelerated profile HMM searches. *PLoS Computational Biology*, 2011.

Sara El-Gebali, Jaina Mistry, Alex Bateman, Sean R. Eddy, Aurélien Luciani, Simon C. Potter, Matloob Qureshi, Lorna J. Richardson, Gustavo A. Salazar, Alfredo Smart, Erik L.L. Sonnhammer, Layla Hirsh, Lisanna Paladin, Damiano Piovesan, Silvio C.E. Tosatto, and Robert D. Finn. The Pfam protein families database in 2019. *Nucleic Acids Research*, 2019.

Dirk Eulberg, Silvia Lakner, Ludmila A. Golovleva, and Michael Schlömann. Characterization of a protocatechuate catabolic gene cluster from Rhodococcus opacus 1CP: Evidence for a merged enzyme with 4- carboxymuconolactone-decarboxylating and 3-oxoadipate enol-lactone- hydrolyzing activity. *Journal of Bacteriology*, 180, 1998.

D. J. Fairley, D. R. Boyd, N. D. Sharma, C. C.R. Allen, P. Morgan, and M. J. Larkin. Aerobic metabolism of 4-hydroxybenzoic acid in Archaea via an unusual pathway involving an intramolecular migration (NIH shift). *Applied and Environmental Microbiology*, 68, 2002.

Stephen J Giovannoni, J Cameron Thrash, and Ben Temperton. Implications of streamlining theory for microbial ecology. *The ISME journal*, 8, 2014.

Rainer Glöckler, Andreas Tschech, and Georg Fuchs. Reductive dehydroxylation of 4-hydroxybenzoyl-CoA to benzoyl-CoA in a denitrifying, phenol-degrading Pseudomonas species. *FEBS Letters*, 251, 1989.

Orland Gonzalez, Susanne Gronau, Friedhelm Pfeiffer, Eduardo Mendoza, Ralf Zimmer, and Dieter Oesterhelt. Systems Analysis of Bioenergetics and Growth of the Extreme Halophile Halobacterium salinarum. *PLoS Computational Biology*, 5, 2009.

Mathieu Groussin, Bastien Boussau, Gergely Szöllõsi, Laura Eme, Manolo Gouy, Céline Brochier-Armanet, and Vincent Daubin. Gene acquisitions from bacteria at the origins of major archaeal clades are vastly overestimated. *Molecular Biology and Evolution*, 33, 2016.

D. H. Haft, B.J. Loftus, D.L. Richardson, F. Yang, J A Eisen, Ian T. Paulsen, and Owen White. TIGRFAMs: a protein family resource for the functional identification of proteins. *Nucleic Acids Research*, 29, 2001.

Diep Thi Hoang, Olga Chernomor, Arndt von Haeseler, Bui Quang Minh, and Le Sy Vinh. UFBoot2: Improving the Ultrafast Bootstrap Approximation. *Molecular Biology and Evolution*, 35, 2018.

Jaime Huerta-Cepas, Kristoffer Forslund, Luis Pedro Coelho, Damian Szklarczyk, Lars Juhl Jensen, Christian Von Mering, and Peer Bork. Fast genome-wide functional annotation through orthology assignment by eggNOG-mapper. *Molecular Biology and Evolution*, 2017.

Philip Jones, David Binns, Hsin Yu Chang, Matthew Fraser, Weizhong Li, Craig McAnulla, Hamish McWilliam, John Maslen, Alex Mitchell, Gift Nuka, Sebastien Pesseat, Antony F. Quinn, Amaia Sangrador-Vegas, Maxim Scheremetjew, Siew Yit Yong, Rodrigo Lopez, and Sarah Hunter. InterProScan 5: Genome-scale protein function classification. *Bioinformatics*, 2014.

M. Kanehisa and S Goto. KEGG: Kyoto Encyclopedia of Genes and Genomes. *Nucleic Acids Research*, 28, 2000.

Nils Kapust, Shijulal Nelson-Sathi, Barbara Schönfeld, Einat Hazkani-Covo, David Bryant, Peter J Lockhart, Mayo Röttger, Joana C Xavier, and William F Martin. Failure to recover major events of gene flux in real biological data due to method misapplication. *Genome Biology and Evolution*, 2018.

Gary M. King. Carbon monoxide as a metabolic energy source for extremely halophilic microbes: Implications for microbial activity in Mars regolith. *Proceedings of the National Academy of Sciences of the United States of America*, 112, 2015.

Gary M. King and Carolyn F. Weber. Distribution, diversity and ecology of aerobic CO-oxidizing bacteria. *Nature Reviews Microbiology*, 5, 2007.

Kira S. Makarova, Yuri I. Wolf, and Eugene V. Koonin. Archaeal clusters of orthologous genes (arCOGs): An update and application for analysis of shared features between thermococcales, methanococcales, and methanobacteriales. *Life*, 5, 2015.

Ana M P Melo, Tiago M Bandeiras, and Miguel Teixeira. New Insights into Type II NAD (P) H : Quinone Oxidoreductases New Insights into Type II NAD (P) H : Quinone Oxidoreductases. *Microbiology and Molecular Biology Reviews*, 68, 2004.

Mary Ann Moran, Alison Buchan, José M. González, John F. Heidelberg, William B. Whitman, Ronald P. Klene, James R. Henriksen, Gary M. King, Robert Belas, Clay Fuqua, Lauren Brinkac, Matt Lewis, Shivani Johri, Bruce Weaver, Grace Pai, Jonathan A. Elsen, Elisha Rahe, Wade M. Sheldon, Wenying Ye, Todd R. Miller, Jane Carlton, David A. Rasko, Ian T. Paulsen, Qinghu Ren, Sean C. Daugherty, Ribert T. Deboy, Robert J. Dodson, A. Scott Durkin, Ramana Madupu, William C. Nelson, Steven A. Sullivan, M. J. Rosovitz, Daniel H. Haft, Jeremy Selengut, and Naomi Ward. Genome sequence of Silicibacter pomeroyi reveals adaptations to the marine environment. *Nature*, 432, 2004.

Shijulal Nelson-Sathi, Tal Dagan, Giddy Landan, Arnold Janssen, Mike Steel, James O. McInerney, Uwe Deppenmeier, and William F Martin. Acquisition of 1,000 eubacterial genes physiologically transformed a methanogen at the origin of Haloarchaea. *Proceedings of the National Academy of Sciences of the United States of America*, 109, 2012.

Shijulal Nelson-Sathi, Filipa L. Sousa, Mayo Roettger, Nabor Lozada-Chávez, Thorsten Thiergart, Arnold Janssen, David Bryant, Giddy Landan, Peter Schönheit, Bettina Siebers, James O. McInerney, and William F. Martin. Origins of major archaeal clades correspond to gene acquisitions from bacteria. *Nature*, 517, 2015.

Christian Rinke, Francesco Rubino, Lauren F. Messer, Noha Youssef, Donovan H. Parks, Maria Chuvochina, Mark Brown, Thomas Jeffries, Gene W. Tyson, Justin R. Seymour, and Philip Hugenholtz. A phylogenomic and ecological analysis of the globally abundant Marine Group II archaea (Ca. Poseidoniales ord. nov.). *ISME Journal*, 13, 2019.

M. H. Saier, C. V. Tran, and R.D. Barabote. TCDB: the Transporter Classification Database for membrane transport protein analyses and information. *Nucleic Acids Research*, 34, 2006.

Beatrix Santiago, Ulrich Schübel, Christine Egelseer, and Ortwin Meyer. Sequence analysis, characterization and CO-specific transcription of the cox gene cluster on the megaplasmid pHCG3 of Oligotropha carboxidovorans. *Gene*, 236, 1999.

Rafael F. Say and Georg Fuchs. Fructose 1,6-bisphosphate aldolase/phosphatase may be an ancestral gluconeogenic enzyme. *Nature*, 464, 2010.

Torsten Seemann. Prokka: Rapid prokaryotic genome annotation. *Bioinformatics*, 2014.

Qunxin She, Rama K. Singh, Fabrice Confalonieri, Yvan Zivanovic, Ghislaine Allard, Mariana J. Awayez, Christina C.Y. Chan-Weiher, Ib Groth Clausen, Bruce A. Curtis, Anick De Moors, Gael Erauso, Cynthia Fletcher, Paul M.K. Gordon, Ineke Heikamp-de Jong, Alex C. Jeffries, Catherine J. Kozera, Nadine Medina, Xu Peng, Hoa Phan Thi-Ngoc, Peter Redder, Margaret E. Schenk, Cynthia Theriault, Niels Tolstrup, Robert L. Charlebois, W. Ford Doolittle, Michel Duguet, Terry Gaasterland, Roger A. Garrett, Mark A. Ragan, Christoph W. Sensen, and John Van Der Oostg. The complete genome of the crenarchaeon Sulfolobus solfataricus P2. *Proceedings of the National Academy of Sciences of the United States of America*, 98, 2001.

Anja Spang, Anja Poehlein, Pierre Offre, Sabine Zumbrägel, Susanne Haider, Nicolas Rychlik, Boris Nowka, Christel Schmeisser, Elena V. Lebedeva, Thomas Rattei, Christoph Böhm, Markus Schmid, Alexander Galushko, Roland Hatzenpichler, Thomas Weinmaier, Rolf Daniel, Christa Schleper, Eva Spieck, Wolfgang Streit, and Michael Wagner. The genome of the ammonia-oxidizing candidatus nitrososphaera gargensis: Insights into metabolic versatility and environmental adaptations. *Environmental Microbiology*, 14, 2012.

Laura Villanueva, Stefan Schouten, and Jaap S.Sinninghe Damsté. Phylogenomic analysis of lipid biosynthetic genes of Archaea shed light on the ‘lipid divide’. *Environmental Microbiology*, 19, 2017.

Supplementary Table 1

All Tara Oceans samples and associated sequencing runs used to reconstruct Hikarchaea MAGs.

Samples that were assembled and of which the contigs were binned are highlighted in blue. Sequencing runs that were used to determine differential coverage for assembled contigs of samples 032_DCM_0.22-1.60, 085_MES_0.22-3 and 124_MIX_0.22-0.45 are highlighted in blue and orange. This table is a subset of the companion Table W1 published in Sunagawa et al, 2015.

Sequencing run(s)	Sample name	Sampling Date/Time [yyyy-mm-ddThh:mm]	Approximate location	Latitude [degrees North]	Longitude [degrees East]	Sampling depth [m]	Environmental Feature	BioSample
ERR599073|ERR599092	TARA_018_DCM_0.22-1.6	2009-11-02T14:07	Mediterranean Sea	35.7528	14.2765	60	(DCM) deep chlorophyll maximum layer (ENVO:01000326)	
ERR594352	TARA_018_DCM_<-0.22	2009-11-02T14:07	Mediterranean Sea	35.7528	14.2765	60	(DCM) deep chlorophyll maximum layer (ENVO:01000326)	
ERR598993|ERR599140	TARA_018_SRF_0.22-1.6	2009-11-02T08:13	Mediterranean Sea	35.759	14.2574	5	(SRF) surface water layer (ENVO:00002042)	
ERR594358	TARA_018_SRF_<-0.22	2009-11-02T08:13	Mediterranean Sea	35.759	14.2574	5	(SRF) surface water layer (ENVO:00002042)	
**ERR599061|ERR599097**	**TARA_032_DCM_0.22-1.6**	**2010-01-11T14:17**	**Red Sea**	**23.4183**	**37.245**	**80**	**(DCM) deep chlorophyll maximum layer (ENVO:01000326)**	**SAMEA2619840**
ERR598994|ERR599144	TARA_067_SRF_0.22-3	2010-09-07T06:19	South Atlantic Ocean	-32.2401	17.7103	5	(SRF) surface water layer (ENVO:00002042)	
ERR594313	TARA_067_SRF_0.22-0.45	2010-09-07T06:19	South Atlantic Ocean	-32.2401	17.7103	5	(SRF) surface water layer (ENVO:00002042)	
ERR594325	TARA_067_SRF_0.45-0.8	2010-09-07T06:19	South Atlantic Ocean	-32.2401	17.7103	5	(SRF) surface water layer (ENVO:00002042)	
ERR594395|ERR594404	TARA_067_SRF_<-0.22	2010-09-07T06:19	South Atlantic Ocean	-32.2401	17.7103	5	(SRF) surface water layer (ENVO:00002042)	
**ERR599008|ERR599125**	**TARA_085_MES_0.22-3**	**2011-01-07T10:39:47**	**Southern Ocean**	**-61.9689**	**-49.5017**	**790**	**(MES) mesopelagic zone (ENVO:00000213)**	**SAMEA2621551**
ERR594284	TARA_122_DCM_0.1-0.22	2011-07-28T17:15:56	South Pacific Ocean	-9.0063	-139.1394	115	(DCM) deep chlorophyll maximum layer (ENVO:01000326)	
ERR598948	TARA_122_DCM_0.22-3	2011-07-28T17:15:56	South Pacific Ocean	-9.0063	-139.1394	115	(DCM) deep chlorophyll maximum layer (ENVO:01000326)	
**ERR594304**	**TARA_122_DCM_0.22-0.45**	**2011-07-28T17:15:56**	**South Pacific Ocean**	**-9.0063**	**-139.1394**	**115**	**(DCM) deep chlorophyll maximum layer (ENVO:01000326)**	**SAMEA2622695**
ERR594301	TARA_122_DCM_0.45-0.8	2011-07-28T17:15:56	South Pacific Ocean	-9.0063	-139.1394	115	(DCM) deep chlorophyll maximum layer (ENVO:01000326)	
ERR594309	TARA_122_MES_0.1-0.22	2011-07-27T19:28:43	South Pacific Ocean	-8.9729	-139.2393	600	(MES) mesopelagic zone (ENVO:00000213)	
ERR598999|ERR599033|ERR599083|ERR599096	TARA_122_MES_0.22-3	2011-07-27T19:28:43	South Pacific Ocean	-8.9729	-139.2393	600	(MES) mesopelagic zone (ENVO:00000213)	
ERR594305	TARA_122_MES_0.22-0.45	2011-07-27T19:28:43	South Pacific Ocean	-8.9729	-139.2393	600	(MES) mesopelagic zone (ENVO:00000213)	
ERR594322	TARA_122_MES_0.45-0.8	2011-07-27T19:28:43	South Pacific Ocean	-8.9729	-139.2393	600	(MES) mesopelagic zone (ENVO:00000213)	
ERR594292	TARA_122_SRF_0.1-0.22	2011-07-26T17:10	South Pacific Ocean	-8.9971	-139.1963	5	(SRF) surface water layer (ENVO:00002042)	
ERR598992	TARA_122_SRF_0.22-3	2011-07-26T17:10	South Pacific Ocean	-8.9971	-139.1963	5	(SRF) surface water layer (ENVO:00002042)	
ERR594307	TARA_122_SRF_0.22-0.45	2011-07-26T17:10	South Pacific Ocean	-8.9971	-139.1963	5	(SRF) surface water layer (ENVO:00002042)	
ERR594306	TARA_122_SRF_0.45-0.8	2011-07-26T17:10	South Pacific Ocean	-8.9971	-139.1963	5	(SRF) surface water layer (ENVO:00002042)	
ERR594293	TARA_123_MIX_0.1-0.22	2011-08-01T17:52:45	South Pacific Ocean	-8.9109	-140.2845	150	(MIX) marine epipelagic mixed layer (ENVO:01000061)	
ERR598956|ERR598998|ERR599117|ERR599157	TARA_123_MIX_0.22-3	2011-08-01T17:52:45	South Pacific Ocean	-8.9109	-140.2845	150	(MIX) marine epipelagic mixed layer (ENVO:01000061)	
ERR594337	TARA_123_MIX_0.22-0.45	2011-08-01T17:52:45	South Pacific Ocean	-8.9109	-140.2845	150	(MIX) marine epipelagic mixed layer (ENVO:01000061)	
ERR594319	TARA_123_MIX_0.45-0.8	2011-08-01T17:52:45	South Pacific Ocean	-8.9109	-140.2845	150	(MIX) marine epipelagic mixed layer (ENVO:01000061)	
ERR599160	TARA_123_SRF_0.22-3	2011-07-31T17:20	South Pacific Ocean	-8.9068	-140.283	5	(SRF) surface water layer (ENVO:00002042)	
ERR594326	TARA_123_SRF_0.22-0.45	2011-07-31T17:20	South Pacific Ocean	-8.9068	-140.283	5	(SRF) surface water layer (ENVO:00002042)	
ERR594347	TARA_123_SRF_0.45-0.8	2011-07-31T17:20	South Pacific Ocean	-8.9068	-140.283	5	(SRF) surface water layer (ENVO:00002042)	
ERR594285	TARA_124_MIX_0.1-0.22	2011-08-05T17:31:19	South Pacific Ocean	-9.0714	-140.5973	120	(MIX) marine epipelagic mixed layer (ENVO:01000061)	
ERR598988|ERR599084|ERR599089|ERR599161	TARA_124_MIX_0.22-3	2011-08-05T17:31:19	South Pacific Ocean	-9.0714	-140.5973	120	(MIX) marine epipelagic mixed layer (ENVO:01000061)	
**ERR594343**	**TARA_124_MIX_0.22-0.45**	**2011-08-05T17:31:19**	**South Pacific Ocean**	**-9.0714**	**-140.5973**	**120**	**(MIX) marine epipelagic mixed layer (ENVO:01000061)**	**SAMEA2622800**
ERR594338	TARA_124_MIX_0.45-0.8	2011-08-05T17:31:19	South Pacific Ocean	-9.0714	-140.5973	120	(MIX) marine epipelagic mixed layer (ENVO:01000061)	
ERR594287	TARA_124_SRF_0.1-0.22	2011-08-04T18:33	South Pacific Ocean	-9.1504	-140.5216	5	(SRF) surface water layer (ENVO:00002042)	
ERR588857|ERR599036|ERR599069|ERR599080|ERR599151	TARA_124_SRF_0.22-3	2011-08-04T18:33	South Pacific Ocean	-9.1504	-140.5216	5	(SRF) surface water layer (ENVO:00002042)	
ERR594311	TARA_124_SRF_0.22-0.45	2011-08-04T18:33	South Pacific Ocean	-9.1504	-140.5216	5	(SRF) surface water layer (ENVO:00002042)	
ERR594296	TARA_124_SRF_0.45-0.8	2011-08-04T18:33	South Pacific Ocean	-9.1504	-140.5216	5	(SRF) surface water layer (ENVO:00002042)	
ERR594341	TARA_125_MIX_0.1-0.22	2011-08-09T17:05:33	South Pacific Ocean	-8.8999	-142.5461	140	(MIX) marine epipelagic mixed layer (ENVO:01000061)	
ERR599156	TARA_125_MIX_0.22-3	2011-08-09T17:05:33	South Pacific Ocean	-8.8999	-142.5461	140	(MIX) marine epipelagic mixed layer (ENVO:01000061)	
ERR594342	TARA_125_MIX_0.22-0.45	2011-08-09T17:05:33	South Pacific Ocean	-8.8999	-142.5461	140	(MIX) marine epipelagic mixed layer (ENVO:01000061)	
ERR594300	TARA_125_MIX_0.45-0.8	2011-08-09T17:05:33	South Pacific Ocean	-8.8999	-142.5461	140	(MIX) marine epipelagic mixed layer (ENVO:01000061)	
ERR594344	TARA_125_SRF_0.1-0.22	2011-08-08T17:33	South Pacific Ocean	-8.9111	-142.5571	5	(SRF) surface water layer (ENVO:00002042)	
ERR599066|ERR599091|ERR599114|ERR599119	TARA_125_SRF_0.22-3	2011-08-08T17:33	South Pacific Ocean	-8.9111	-142.5571	5	(SRF) surface water layer (ENVO:00002042)	
**ERR594339**	**TARA_125_SRF_0.22-0.45**	**2011-08-08T17:33**	**South Pacific Ocean**	**-8.9111**	**-142.5571**	**5**	**(SRF) surface water layer (ENVO:00002042)**	**SAMEA2622822**
ERR594323	TARA_125_SRF_0.45-0.8	2011-08-08T17:33	South Pacific Ocean	-8.9111	-142.5571	5	(SRF) surface water layer (ENVO:00002042)	

Supplementary Table 2
Bin characteristics.

Overview of genome and assembly characteristics for the 5 MAGs of Hikarchaea.

**Bin**	**Candidatus Hikarchaeum yamanae Bin1**	**Candidatus Hikarchaeum yamanae Bin2**	**Candidatus Hikarchaeum yamanae Bin3**	**Candidatus Hikarchaeum sp. Bin4**	**Candidatus Hikarchaeum sp. Bin5**
preliminary name	bin_91	bin_172	bin_117	bin_125	bin_160
Sample	122 DCM 0.22-0.45	124 MIX 0.22-0.45	125 SRF 0.22-0.45	085 MES 0.22-3	032 DCM 0.22-1.60
Contigs	9	20	186	183	36
Largest contig	367408	192843	16980	33637	143609
N50	267266	110043	6001	5520	55489
Assembly size	1131970	1122501	1007431	920269	1067804
Completeness	0.98	0.97	0.77	0.75	0.93
Redundacy	1.00	1.00	1.01	1.00	1.00
Estimated genome size (Mb)	1.2	1.2	1.2	1.2	1.2
CDS	1176	1166	1156	1096	1130
rRNA	5S,23S,16S	5S,23S,16S	5S,23S,16S	5S(partial),23S,16S	-
tRNA	40	40	33	34	35
%GC	41.6	41.5	41.5	40.7	40.2
N-ARSC*	0.311	0.311	0.309	0.312	0.314
Coding density (%)	94.0	93.9	94.0	93.1	94.0
Median intergenic space (bp)	40	40	39	42	40
MIMAG classification	High-quality draft	High-quality draft	Medium-quality draft	Medium-quality draft	Medium-quality draft

* N-ARSC (Nitrogen Atoms per Residue Side Chain): the mean number of Nitrogen atoms per amino acid side chain in the predicted proteome

Supplementary Table 3
Overview of Bayesian phylogenetic analyses
Summary statistics of alignments, MCMC sampling and posterior predictive tests of all Bayesian phylogenetic analyses

**Dataset: RP56 Euryarchaeota**	**Untreated**	**SR4 recoded**	**50% least heterogeneous sites (χ2trimmed)**	**Haloarchaea and Hikarchaea removed**	**Methanotecta-only (untreated)**	**Methanotecta-only (SR4 recoded)**	**Methanotecta-only, 30% least heterogeneous sites**
General alignment statistics							
χ^2-score	19706	2371	2390	10081	10430	1583	1111
Sites	7353	7353	3677	6820	7811	7811	5468
Informative sites	6772	5704	3107	5977	6164	4528	3830
Missing data (%)	9.3	9.3	9.2	9.4	5.9	5.9	5.6
Model of evolution	CAT+GTR+Γ4	CAT+GTR+Γ4	CAT+GTR+Γ4	CAT+GTR+Γ4	CAT+LG+Γ4	CAT+GTR+Γ4	CAT+LG+Γ4
MCMC sampling statistics							
Cycles	20800	46300	45200	30400	20800	26100	19000
Burnin	5000	5000	5000	5000	5000	5000	5000
Effective sample size*	>105	>572	>395	>207	>1131	>4300	>820
Maxdiff	1	1	0.56	1	0.15	0.08	0.21
*Posterior predictive tests***							
Maximum squared heterogeneity (p-value)	0	0	0 - 0.005	0	0	0	0 - 0.004
Maximum squared heterogeneity (z-score)	7.9 - 8.9	8.8 - 9.5	3.5 - 4.0	8.9 - 10.5	66.7 - 75.1	25.6 - 27.8	4.6 - 5.3
Mean squared heterogeneity (p-value)	0	0	0	0	0	0	0
Mean squared heterogeneity (z-score)	23.8 - 28.0	21.2 - 23.7	13.1 - 13.4	52.8 - 54.1	253.9 - 269.0	116.4 - 127.2	22.1 - 25.6
Mean diversity per site (p-value)	0	0.75 - 0.77	0	0	0	0.65 - 0.73	0 - 0.004
Mean diversity per site (z-score)	7.7 - 8.5	-0.75 - -0.69	5.7 - 6.1	6.3-6.7	3.4 - 3.7	-0.48 - -0.61	3.2 - 3.5

* Considering only log-likelihood, total tree length, alpha parameter and number of categories
** Range across 4 chains

Supplementary Table 4

AU test and bootstrap support for alternative placements of Methanonatro

Support expressed in bootstrap and AU test P-values for three hypotheses regarding the placement of Methanonatronarchaeia, Haloarchaea and Hikarchaea. Rejected topologies (P<0.05).

		MNA-basal		*MNA+HA+HIK-basal*		*MNA+HA+HIK-within*	
**Dataset**	**Model**	**Bootstrap%****	**AU-test P-value**	**Bootstrap%****	**AU-test P-value**	**Bootstrap%****	**AU-test P-value**
untreated	LG+C60+F+Γ4	27	0.697	62	0.793	11	0.497
	PMSF*	12	0.308	83	0.938	5	0.275
50% least heterogenous sites	LG+C60+F+Γ4	98	0.773	22	0.205	0	0.097
	PMSF*	99	0.983	1	0.335	0	0.023
Haloarchaea and MG-IV removed	LG+C60+F+Γ4	100§	0.907§	100§	0.903§	0	0.000008
	PMSF*	100§	0.963§	100§	0.962§	0	0.018

* PMSF approximation of the LG+C60+F+Γ4 model

**LG+C60+F+Γ4: Ultra-fast bootstrap, PMSF approximation: Non-parametric bootstrap

§ Because Haloarchaea and Hikarchaea are removed, MNA+HA+HIK-basal and MNA-basal are identical topological constraints

Topologies

MNA-basal	Defined by the node ((MNA),(HA+HIK+AG+MTT),(EURY+TACK));
MNA+HA+HIK-basal	Defined by the node ((MNA+HA+HIK),(MTT+AG),(EURY+TACK));
MNA+HA+HIK-within	Defined by the node ((MNA+HA+HIK),(MTT),(AG+EURY+TACK));

Taxon abbreviations

MNA	Methanonatronarchaeia
HA	Haloarchaea
HIK	Hikarchaea
AG	Archaeoglobi
MTT	other Methanotecta
EURY	all non-Methanotecta Euryarchaeota
TACK	TACK archaea outgroup

Supplementary Table 5
From Santiago et al., 1999

Gene	PFAM	Uniprot link to gene in Oligotropha carboxidovorans	candidate genes in Hikarchaeia
CoxB	PF13557	https://www.uniprot.org/uniprot/Q9KX28	no
CoxC	PF04397, PF03707	https://www.uniprot.org/uniprot/Q9KX27	no
**CoxD**	**PF07728**	**https://www.uniprot.org/uniprot/Q51326**	**yes**
**CoxE**	**PF05762**	**https://www.uniprot.org/uniprot/Q9KX26**	**yes**
**CoxF**	**PF13478, PF02625**	**https://www.uniprot.org/uniprot/Q9KX25**	**yes**
**CoxG**	**PF06240**	**https://www.uniprot.org/uniprot/Q9KX24**	**yes**
CoxH	PF03707, PF04397	https://www.uniprot.org/uniprot/F8C101	no
**CoxI**	**PF13478, PF02625**	**https://www.uniprot.org/uniprot/F8C102**	**yes**
**CoxK**	**PF00892**	**https://www.uniprot.org/uniprot/Q9KX21**	**yes**

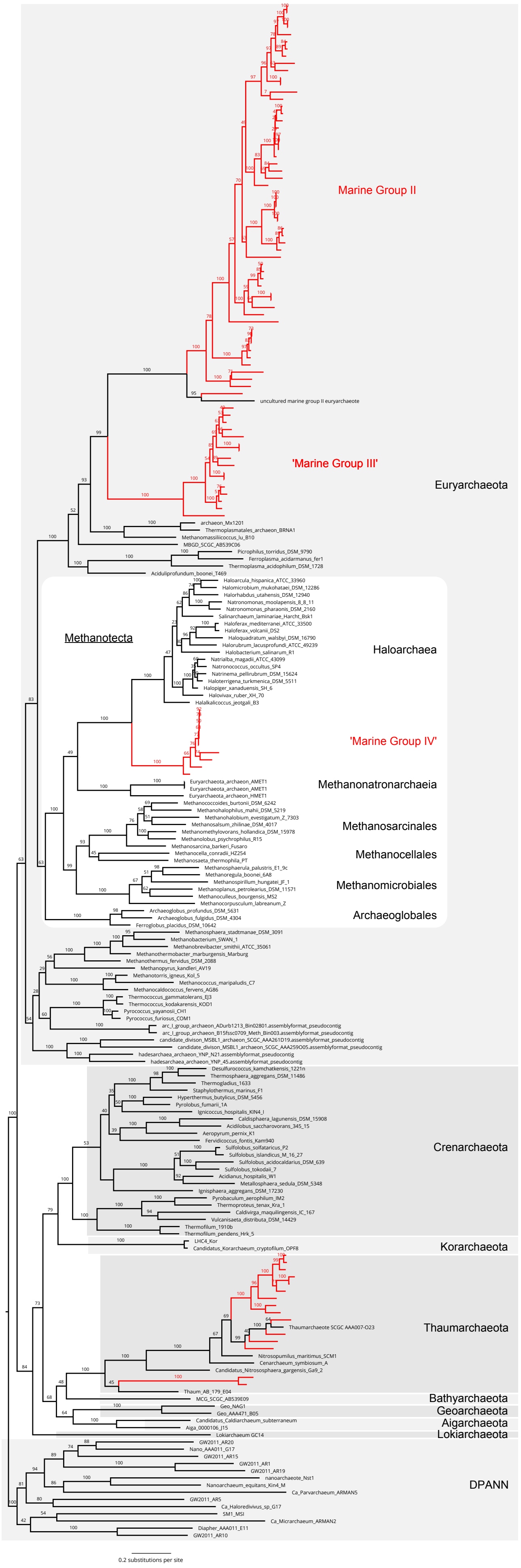

Supplementary Figure 1: Archaeal phylogenetic diversity in selected samples. Archaeal phylogenetic diversity present in the metagenome assemblies of the five Tara Ocean's environmental samples '032 DCM 0.22-1.60', '122 DCM 0.22-0.45', '125 SRF 0.22-0.45', '124 MIX 0.22-0.45' and '085 MES 0.22-3'. Phylogenetic tree was constructed with the 'RP15 Pipeline' (see Methods). Reference taxa (black), sample taxa (red).

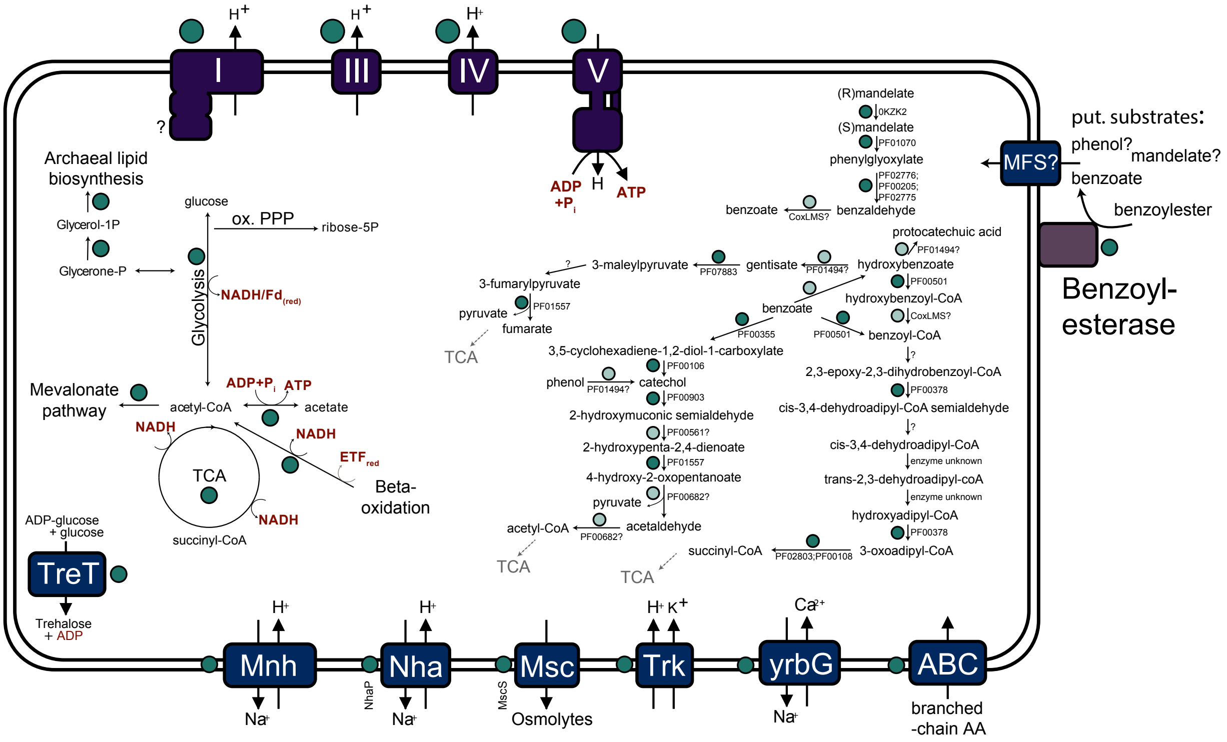

Supplementary Figure 2: Metabolic map of Hikarchaeia. Hikarchaeia were inferred to represent aerobic organoheterotrophs, that may have the potential to among others use aromatics such as benzoate as growth substrates. Dark green circles indicate the presence of a pathway or a clear homologue for a certain reaction. Light green circles indicate the putative presence of a protein family or candidate protein for a certain step, which however could also function in another context. Please refer to Supplementary Table 10 (and 11) for further details.

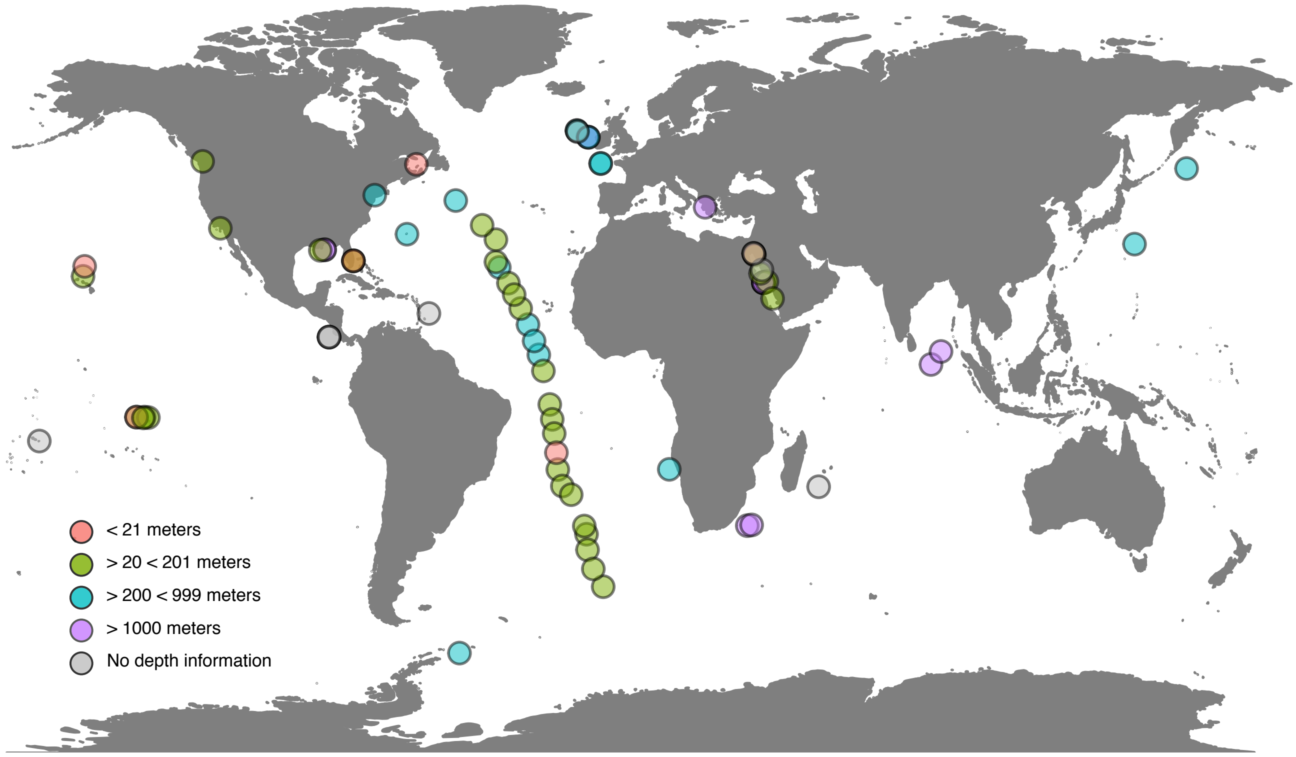

Supplementary Figure 3: Geographic distribution of Hikarchaea 16S rRNA gene sequences. Locations and depths of all samples associated with reads that exhibit ≥93% sequence similarity over ≥400 bp to 16S rRNA gene sequences of the Hikarchaea (Marine Group IV) MAGs.

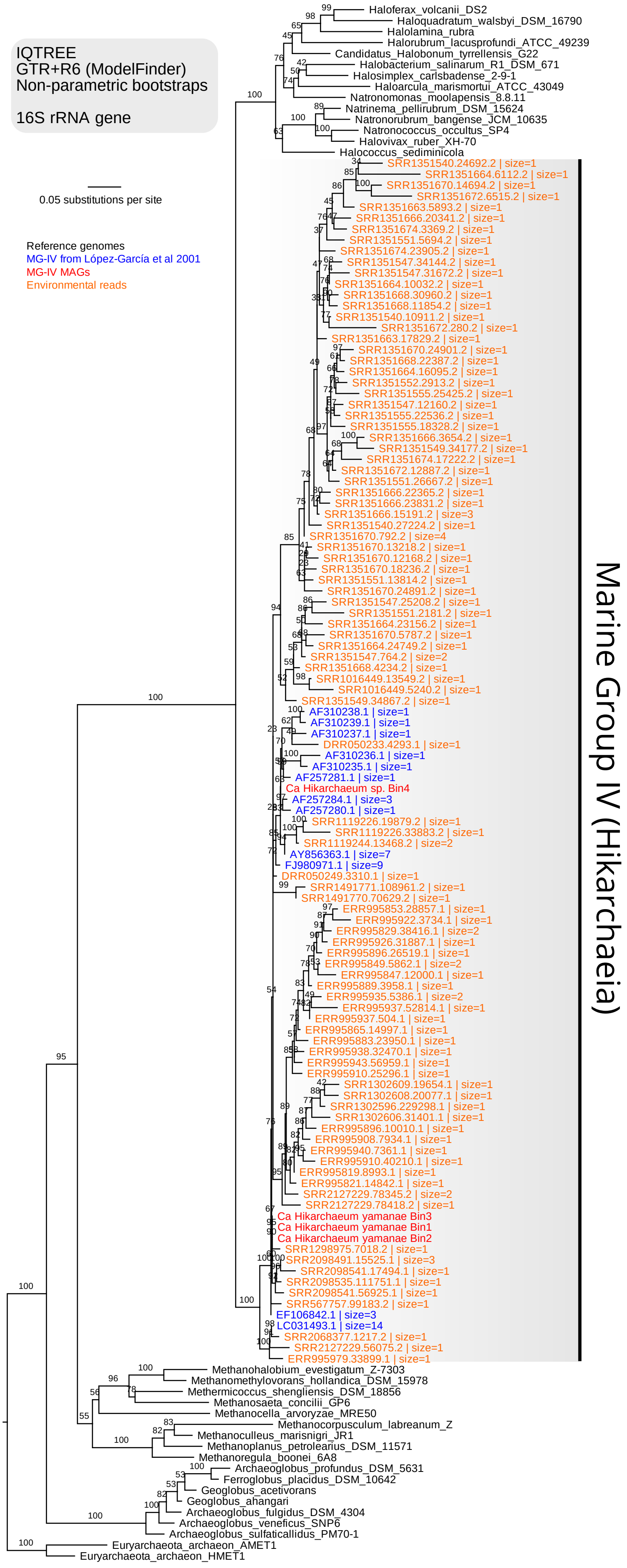

Supplementary Figure 4: 16S rRNA gene maximum likelihood phylogeny depicting Hikarchaea sampled diversity. ML trees inferred under GTR+R6 with 100 nonparametric bootstraps as implemented by IQTREE on the 16S rRNA gene alignment from reference taxa of the Methanotecta (black), the first reported 16S sequences of Hikarchaea (as Marine Group IV) (Lopez-Garcia et al, 2001; blue), the Hikarchaea MAGs (red) as well as environmental sequences (orange). Major groupings within Hikarchaea are labelled (α-ϵ).

rp56.eury.noHalanaero.cct.untr.aln.treefile.nms

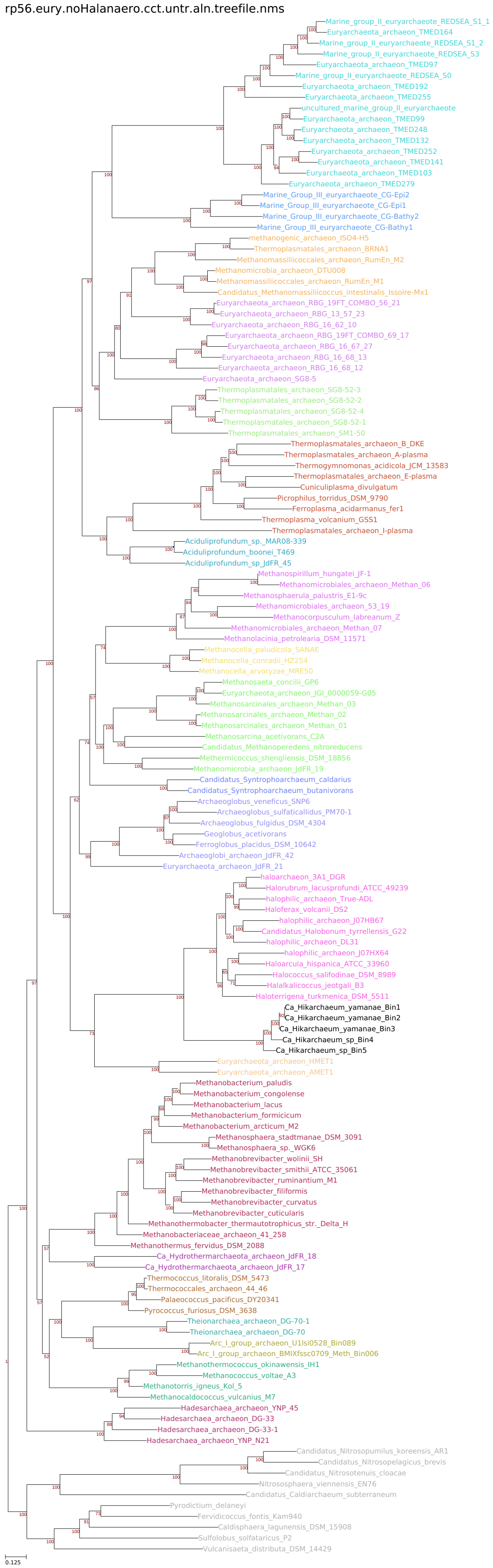

(Supplementary Figure 5a)

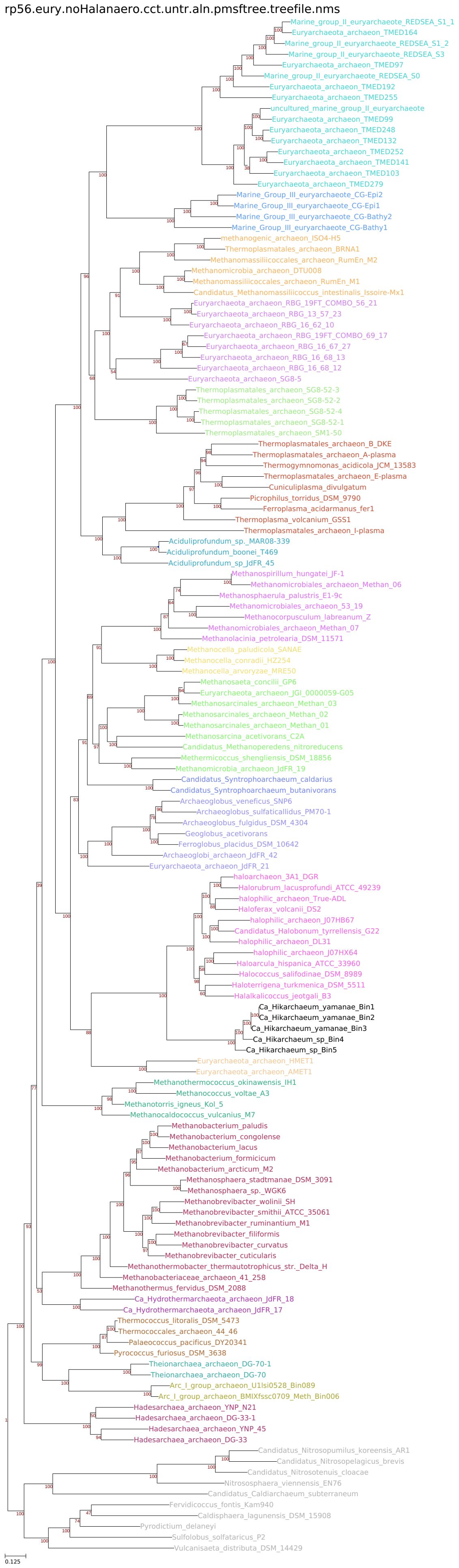

(Supplementary Figure 5b)

Supplementary Figure 5: Maximum likelihood phylogeny of Euryarchaeota based on the untreated concatenated alignment of 56 ribosomal proteins. ML trees inferred under LG+C60+F+Γ4 with ultra-fast-bootstraps (a) and its PMSF approximation with non-parametric bootstraps (b) as implemented by IQTREE on the untreated alignment derived from the concatenation of 56 ribosomal proteins across Euryarchaeota (Zaremba-Niedźwiedzka et al. 2017).

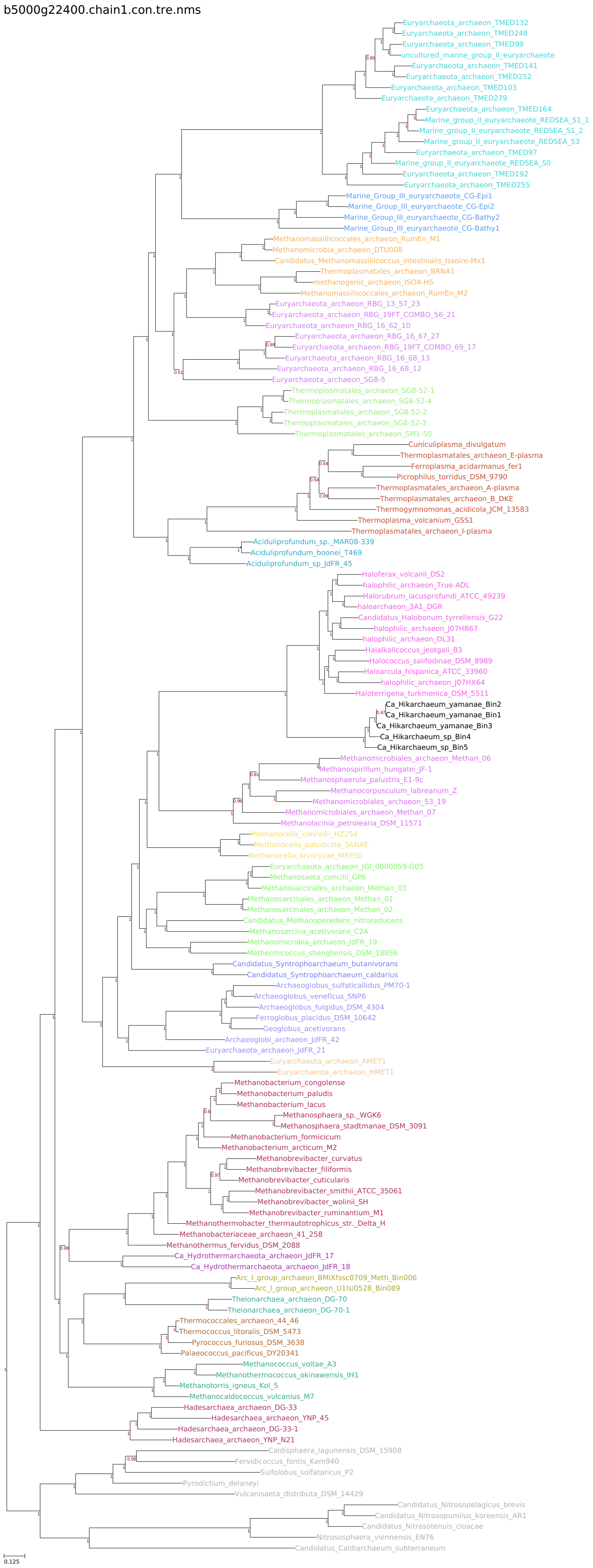

(Supplementary Figure 6a)

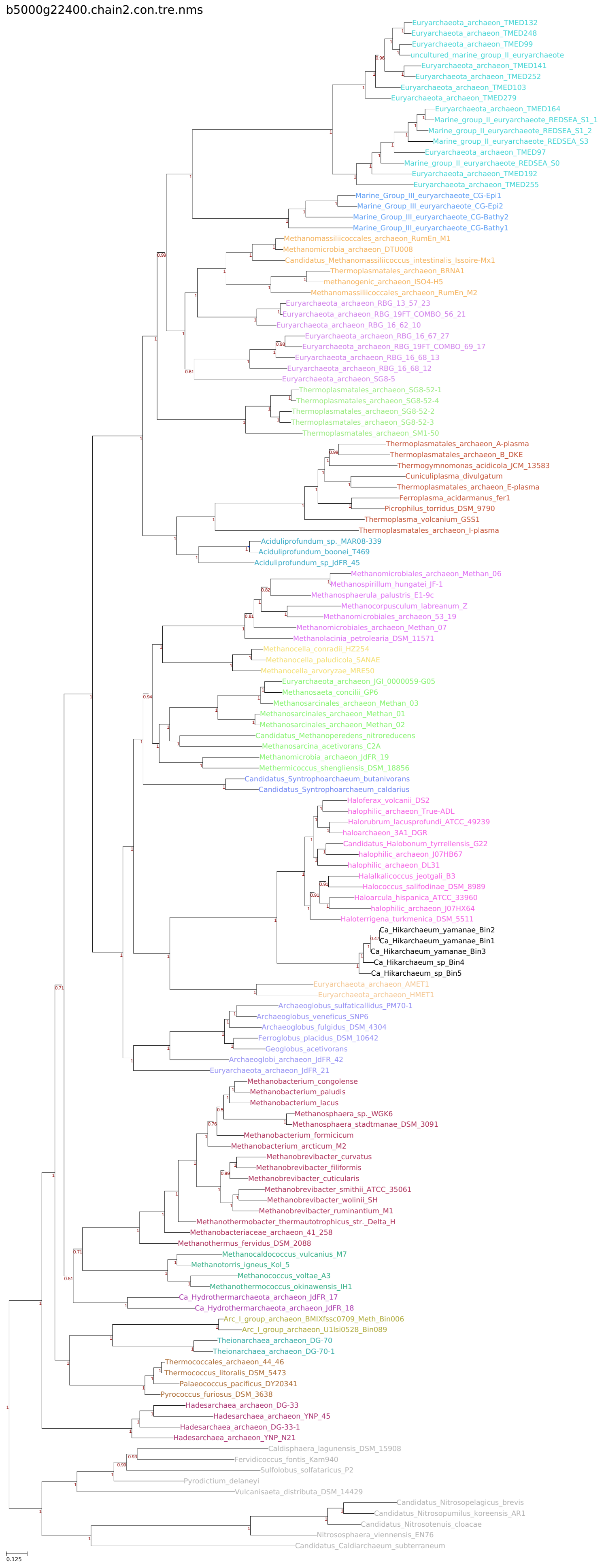

(Supplementary Figure 6b)

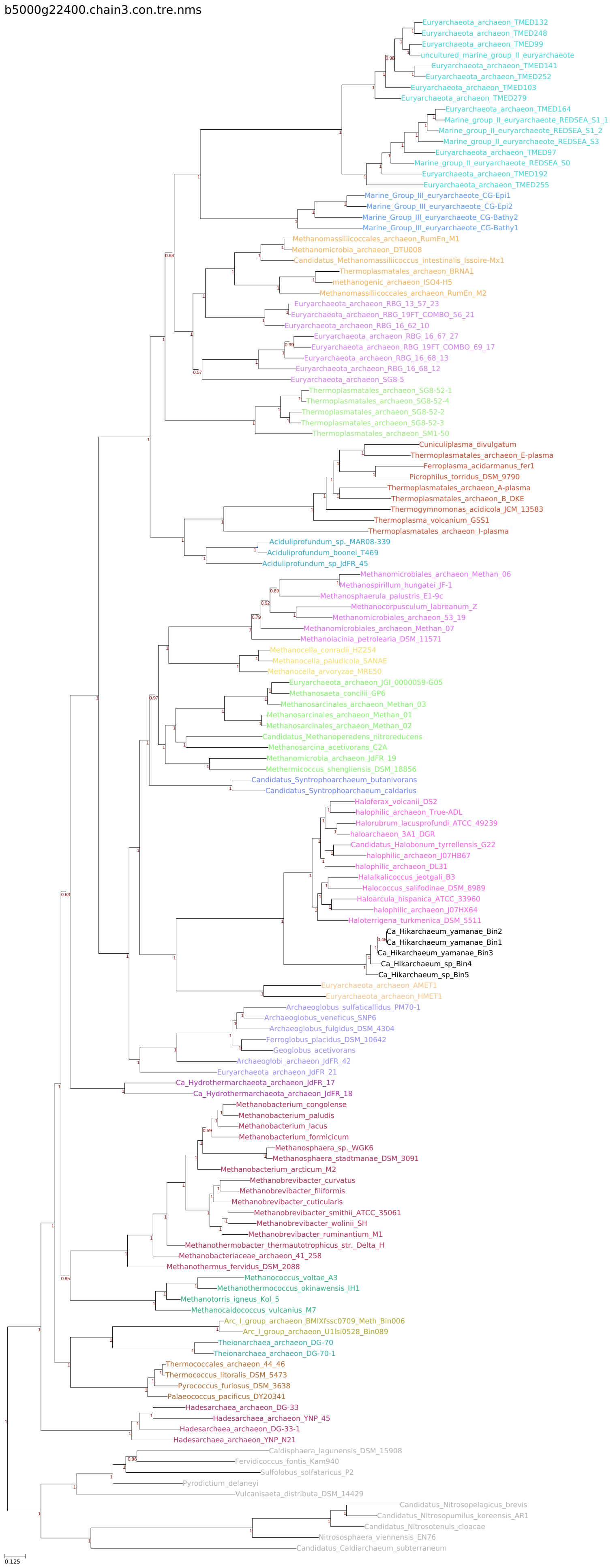

(Supplementary Figure 6c)

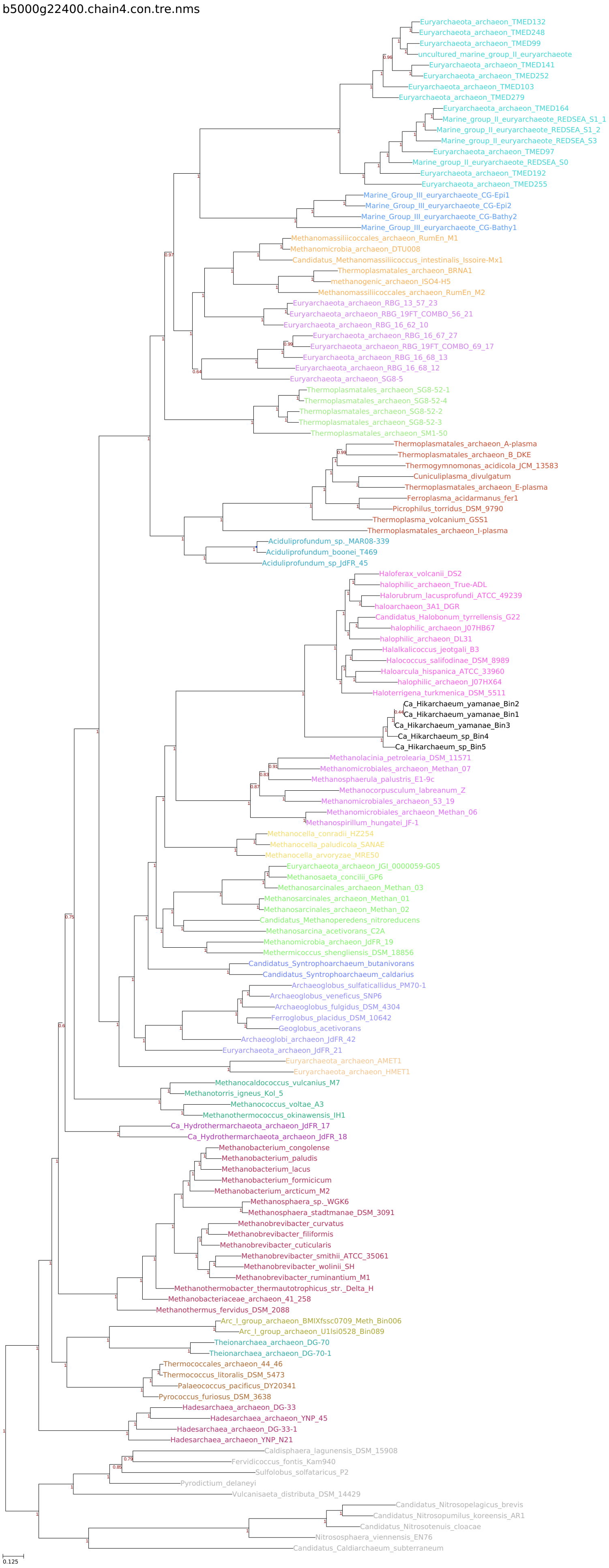

(Supplementary Figure 6d)

Supplementary Figure 6: Bayesian inference of Euryarchaeota phylogeny based on the untreated concatenated alignment of 56 ribosomal proteins. Consensus trees of each of the 4 MCMC chains (a-d) that were inferred under CAT+GTR+$Gamma$4 on the untreated alignment derived from the concatenation of 56 ribosomal proteins across Euryarchaeota (Zaremba-Niedźwiedzka et al. 2017).

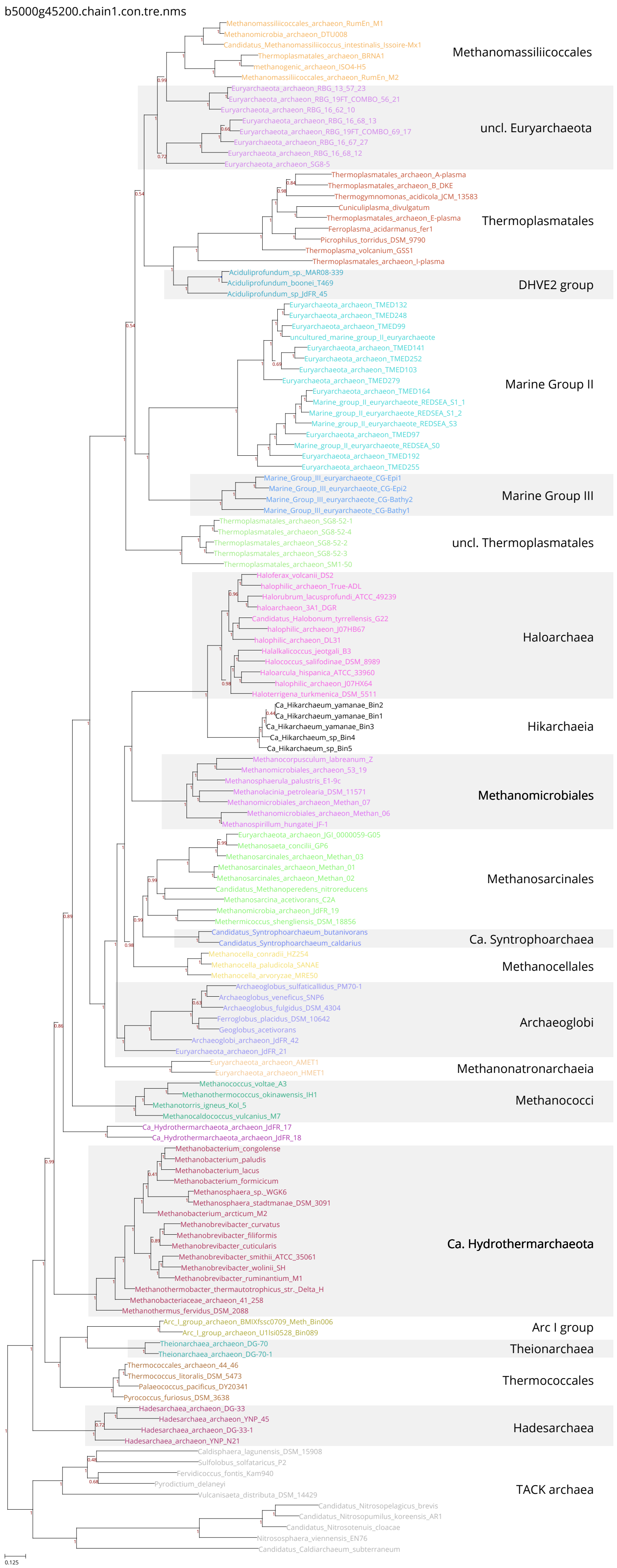

(Supplementary Figure 7a)

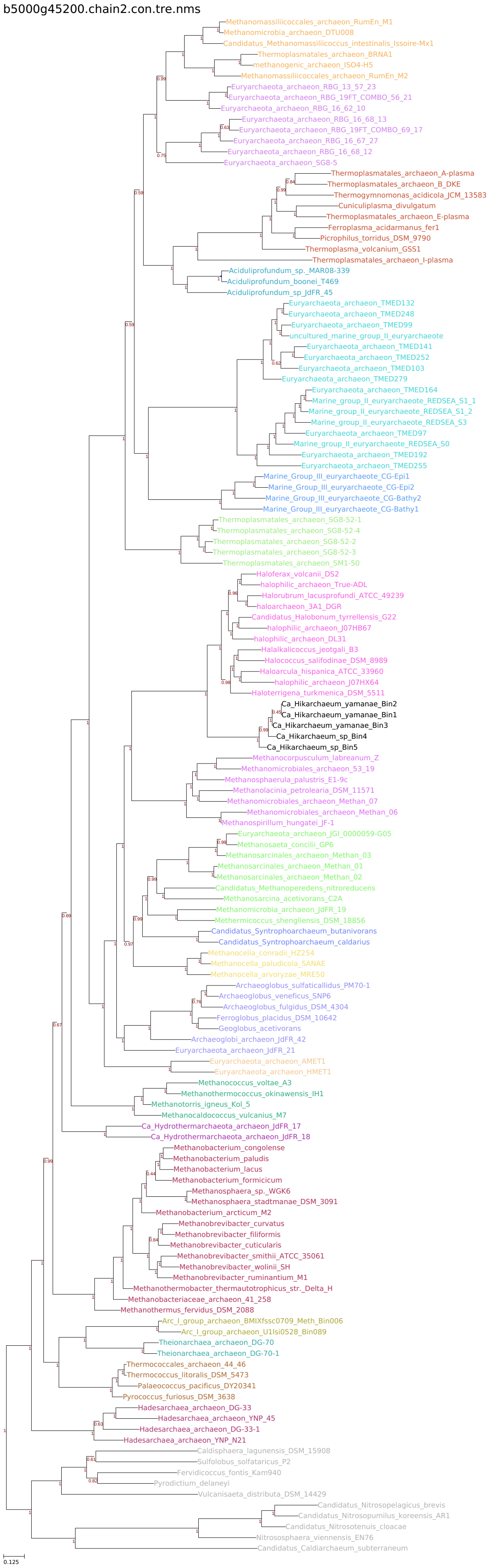

(Supplementary Figure 7b)

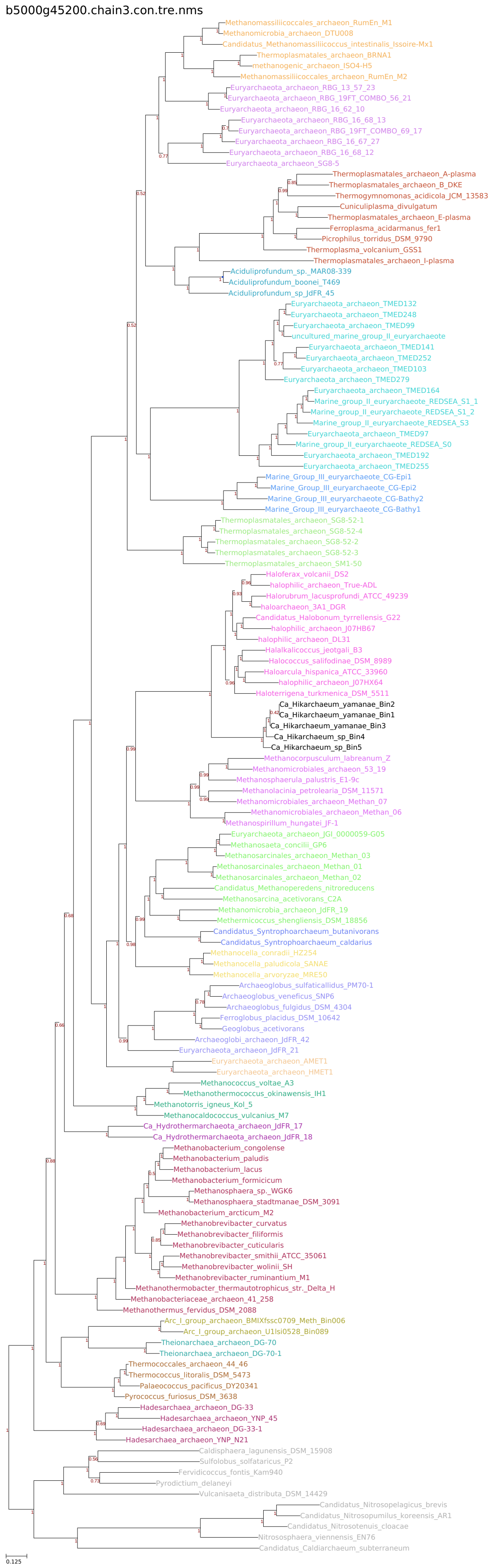

(Supplementary Figure 7c)

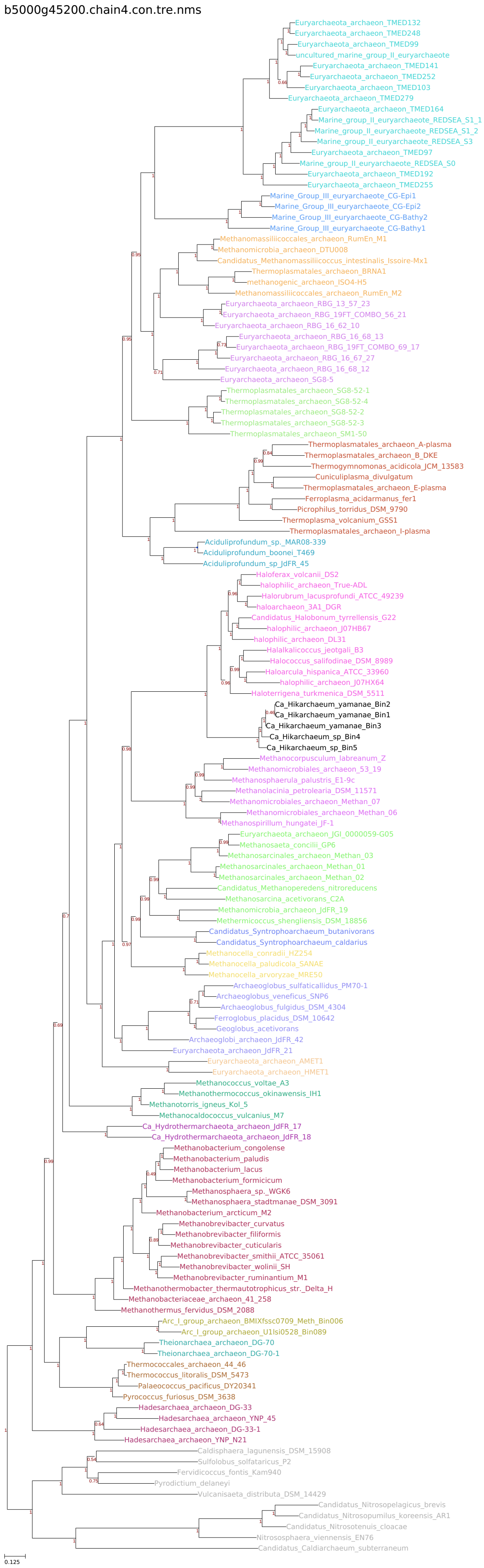

(Supplementary Figure 7d)

Supplementary Figure 7: Bayesian inference of Euryarchaeota phylogeny based on the χ^2-trimmed (50% most heterogeneous sites removed) concatenated alignment of 56 ribosomal proteins. Consensus trees of each of the 4 MCMC chains (a-d) that were inferred under CAT+GTR+Γ4 on the untreated alignment derived from the concatenation of 56 ribosomal proteins across Euryarchaeota (Zaremba-Niedźwiedzka et al. 2017).

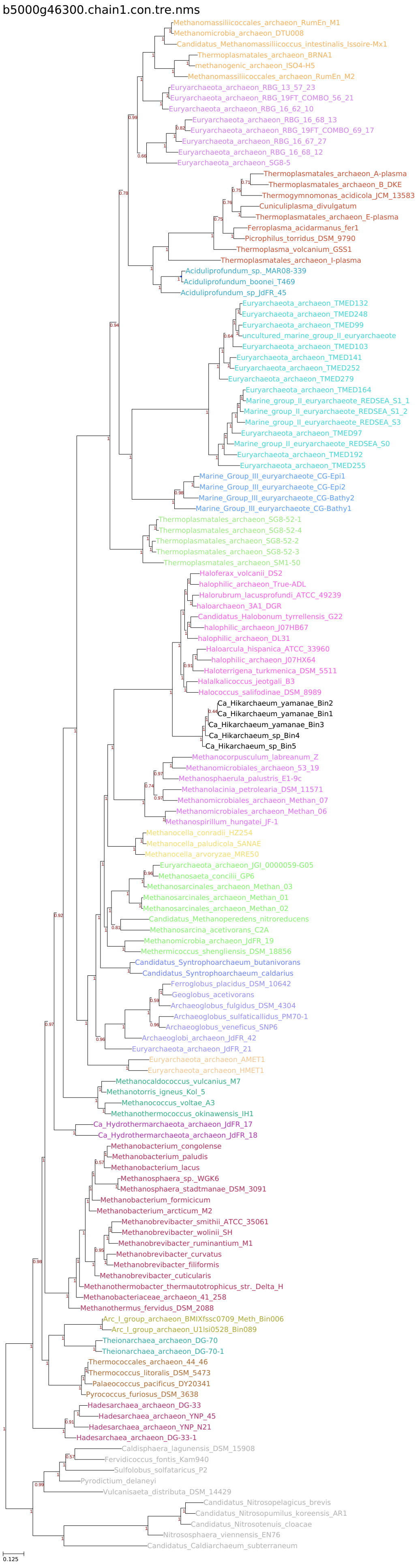

(Supplementary Figure 8a)

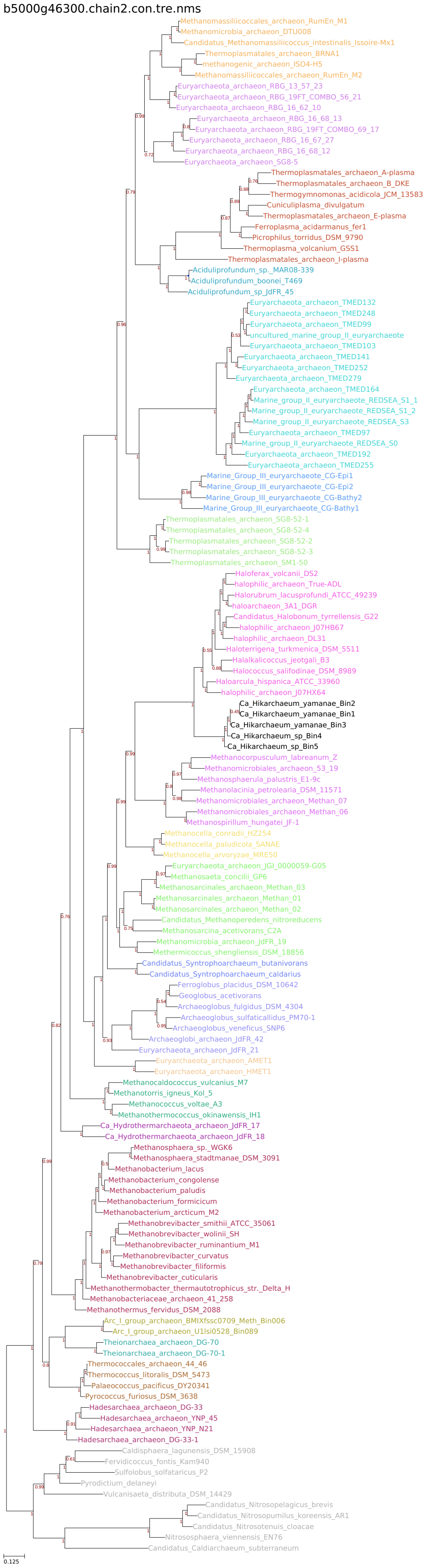

(Supplementary Figure 8b)

b5000g46300.chain3.con.tre.nms

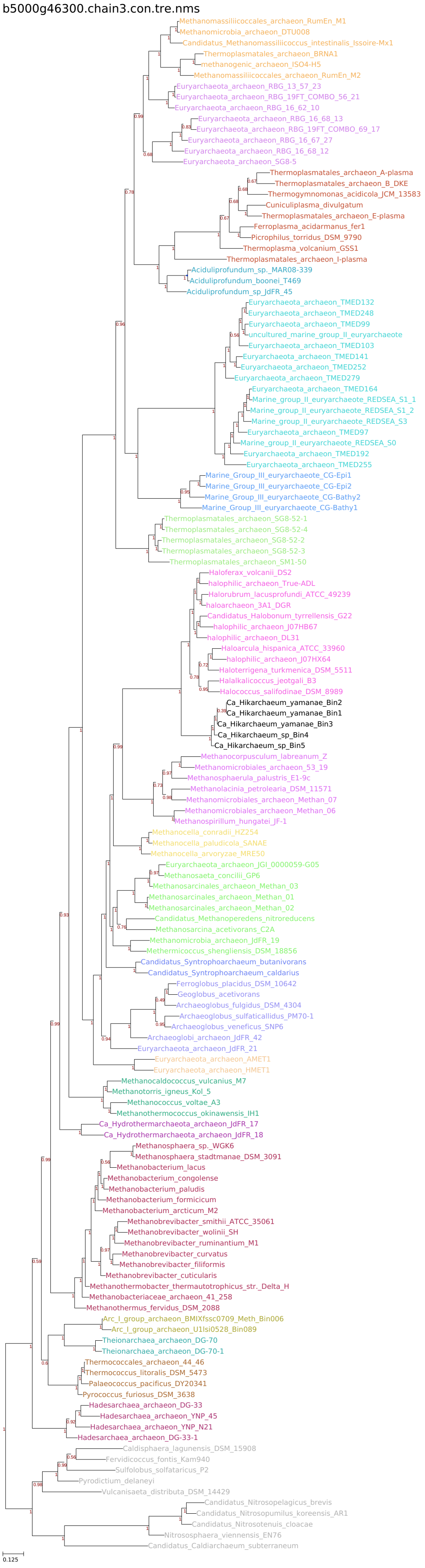

(Supplementary Figure 8c)

b5000g46300.chain4.con.tre.nms

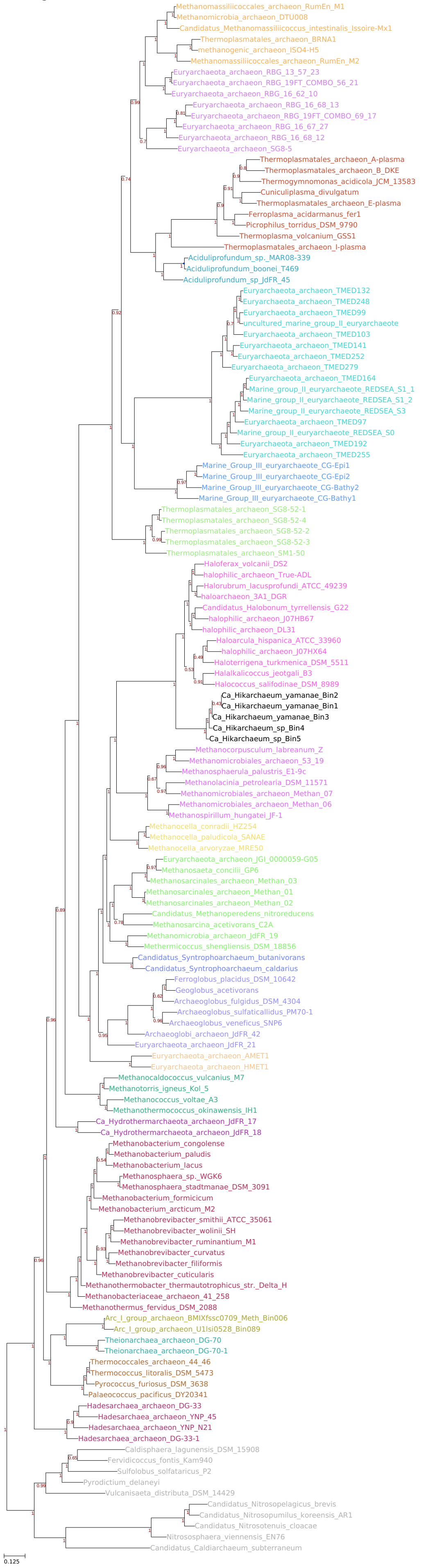

(Supplementary Figure 8d)

Supplementary Figure 8: Bayesian inference of Euryarchaeota phylogeny based on the SR4-recoded concatenated alignment of 56 ribosomal proteins. Consensus trees of each of the 4 MCMC chains (a-d) that were inferred under CAT+GTR+Γ4 on the SR4-recoded alignment derived from the concatenation of 56 ribosomal proteins across Euryarchaeota (Zaremba-Niedźwiedzka et al. 2017).

rp56.eury.noHalanaero.cct.f500.aln.treefile.nms

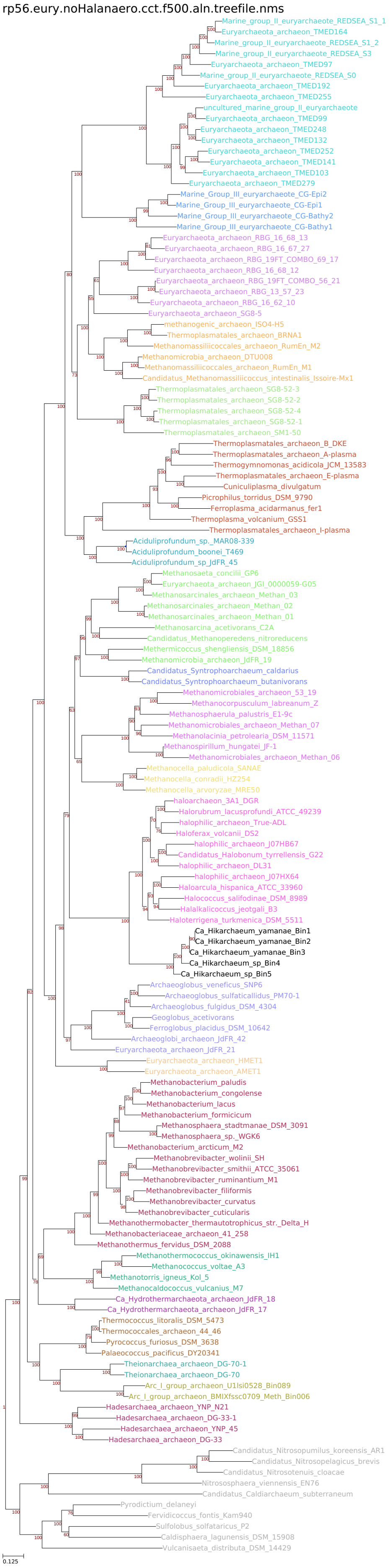

(Supplementary Figure 9a)

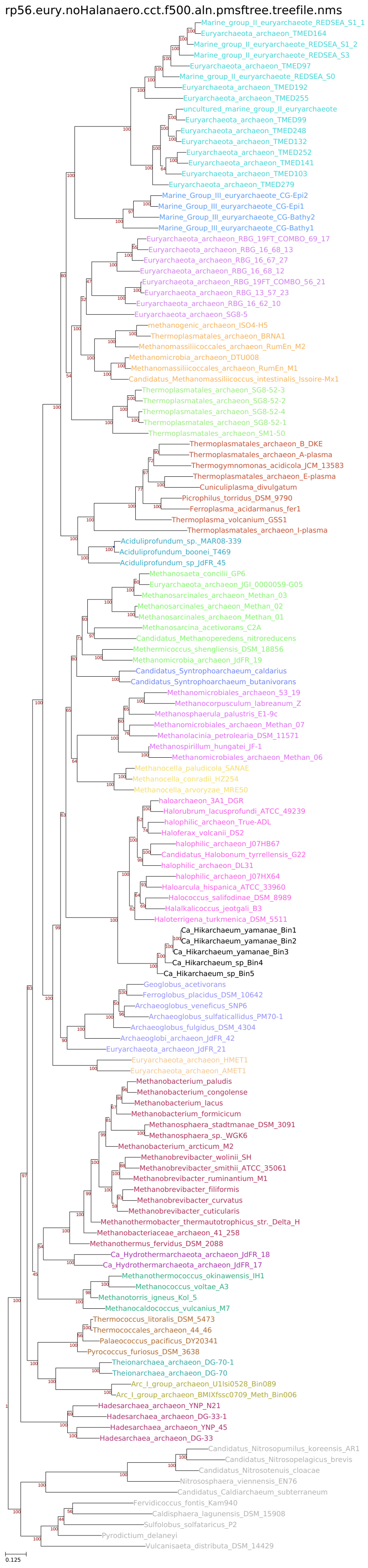

(Supplementary Figure 9b)

Supplementary Figure 9: Maximum likelihood phylogeny of Euryarchaeota based on the χ^2-trimmed (50% most heterogeneous sites removed) concatenated alignment of 56 ribosomal proteins. ML trees inferred under LG+C60+F+Γ4 with ultra-fast-bootstraps (a) and its PMSF approximation with non-parametric bootstraps (b) as implemented by IQTREE on the chi^2-trimmed (50% most heterogeneous sites removed) alignment derived from the concatenation of 56 ribosomal proteins across Euryarchaeota (Zaremba-Niedźwiedzka et al. 2017).

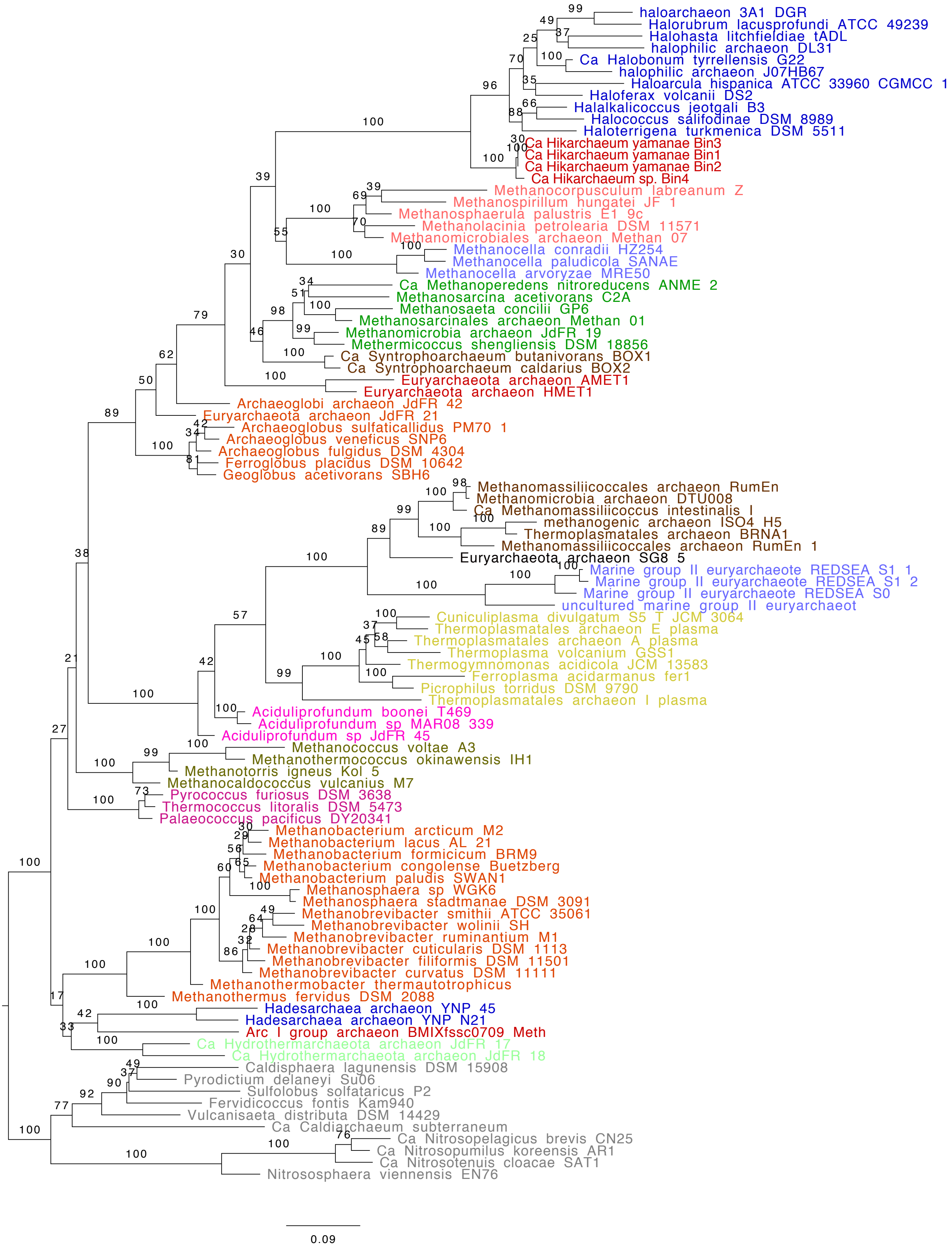

Supplementary Figure 10: 16S rRNA gene maximum likelihood phylogeny. ML trees inferred under GTR+F+R6 with 100 nonparametric bootstraps as implemented by IQTREE on the 16S rRNA gene alignment of Methanotecta taxa included in the species tree.

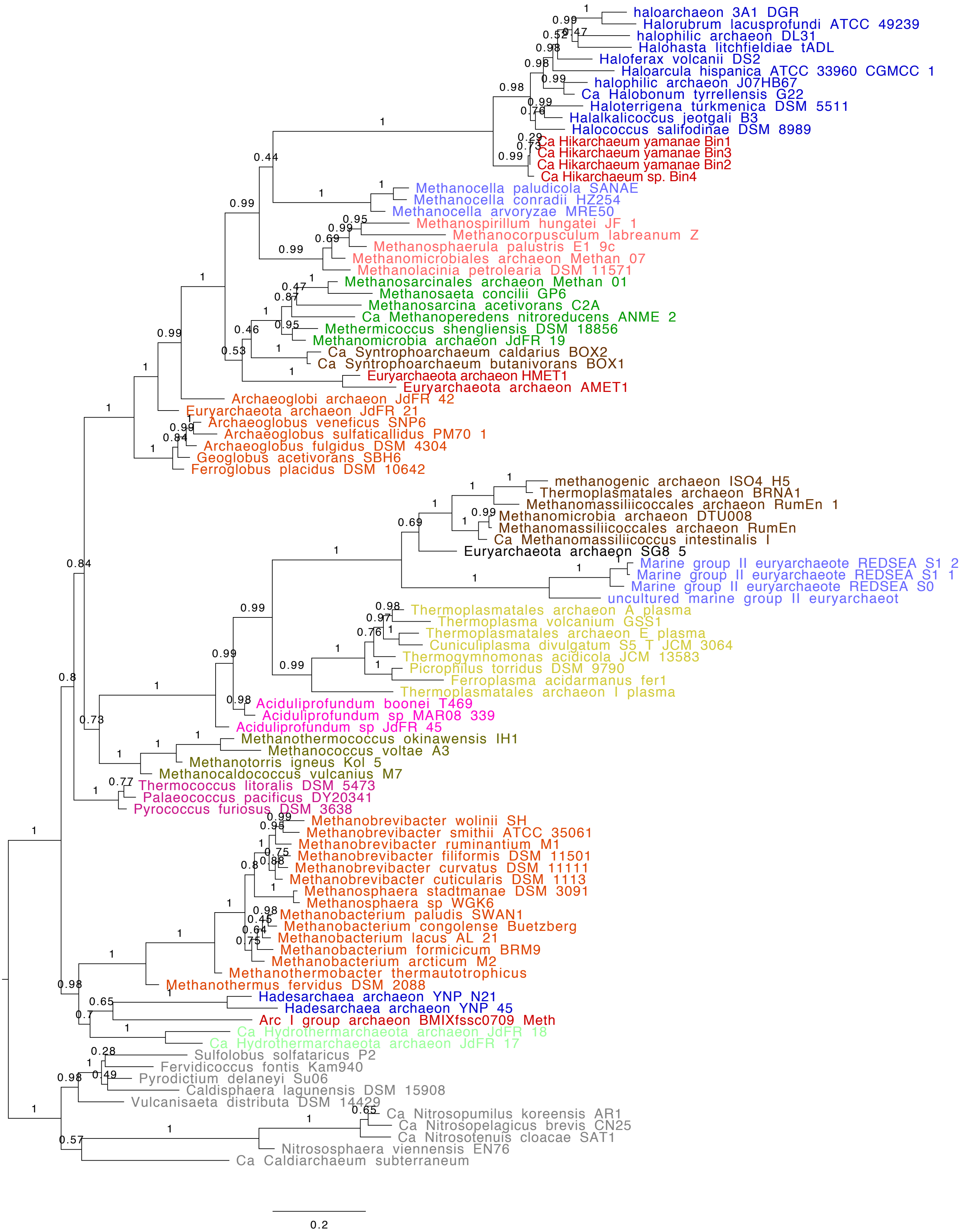

Supplementary Figure 11: 16S rRNA gene Bayesian phylogeny. Consensus tree of 4 MCMC chains that were inferred under CAT+GTR+Γ4 by Phylobayes on the 16S rRNA gene alignment of Methanotecta taxa included in the species tree.

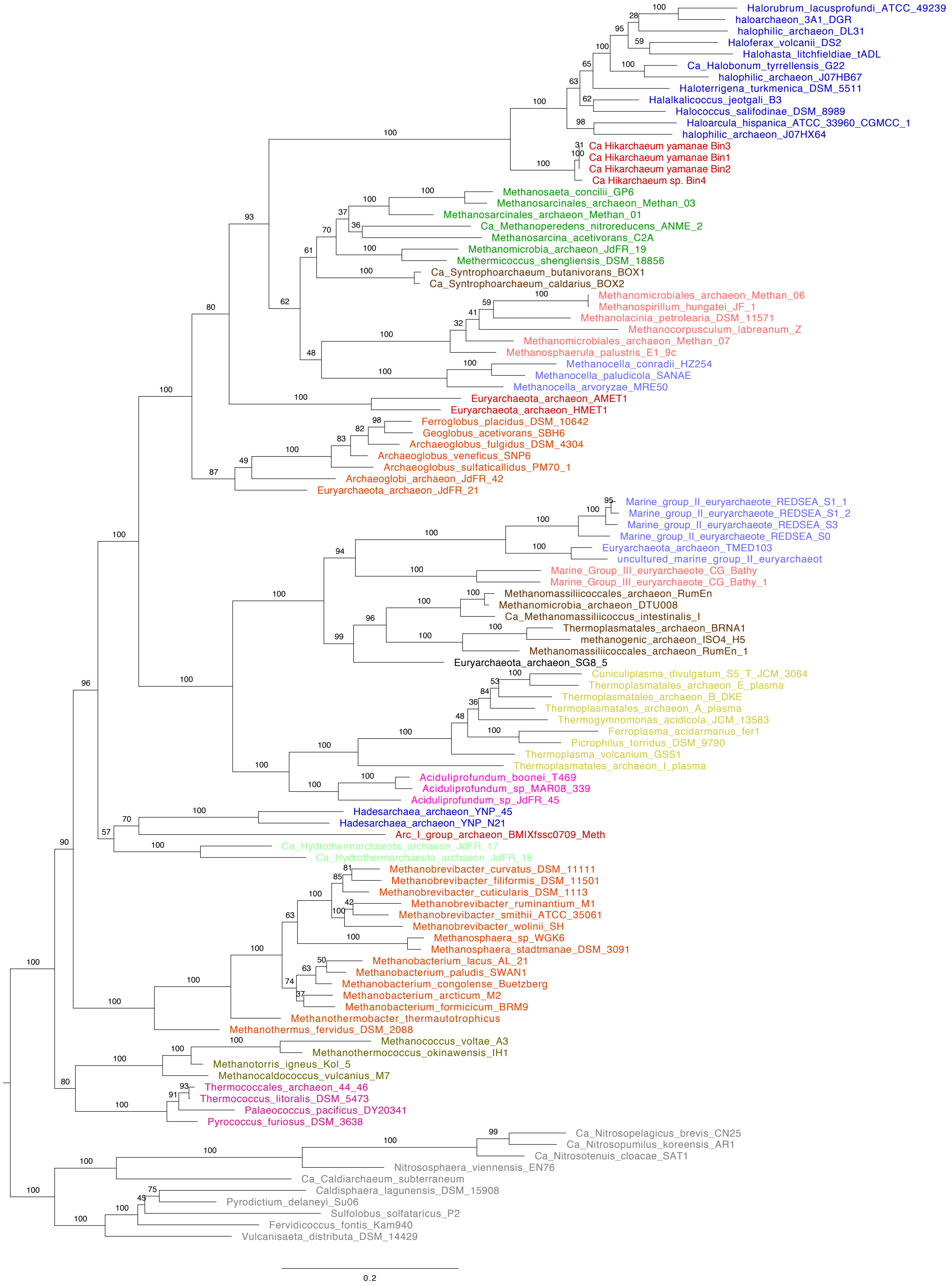

Supplementary Figure 12: 16S+23S rRNA gene maximum likelihood phylogeny. ML trees inferred under GTR+F+R7 with 100 nonparametric bootstraps as implemented by IQTREE on the concatenated 16S and 23S rRNA gene alignment of Methanotecta taxa included in the species tree.

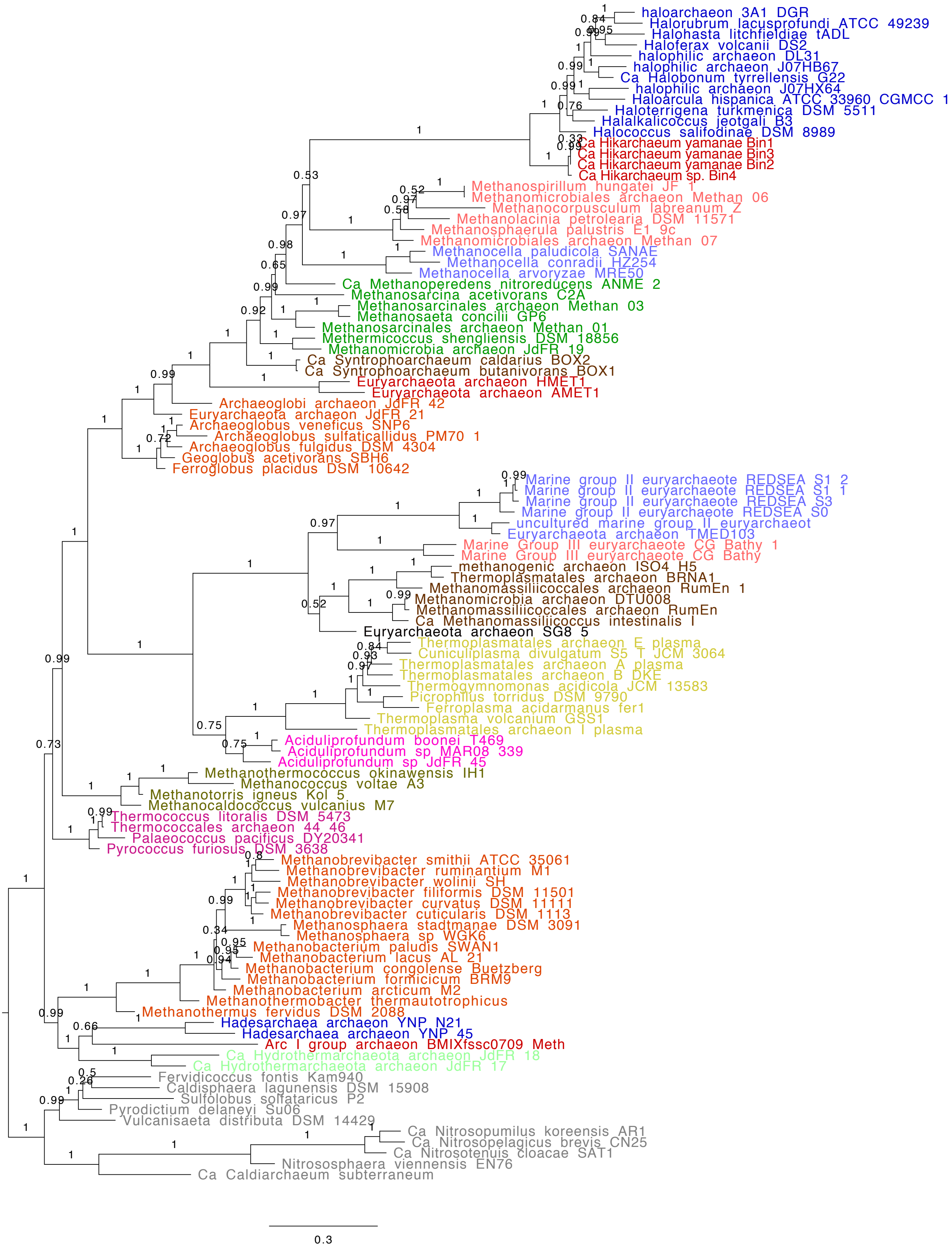

Supplementary Figure 13: 16S+23S rRNA gene Bayesian phylogeny. Consensus tree of 4 MCMC chains that were inferred under CAT+GTR+Γ4 by Phylobayes on the concatenated 16S and 23S rRNA gene alignment of Methanotecta taxa included in the species tree.

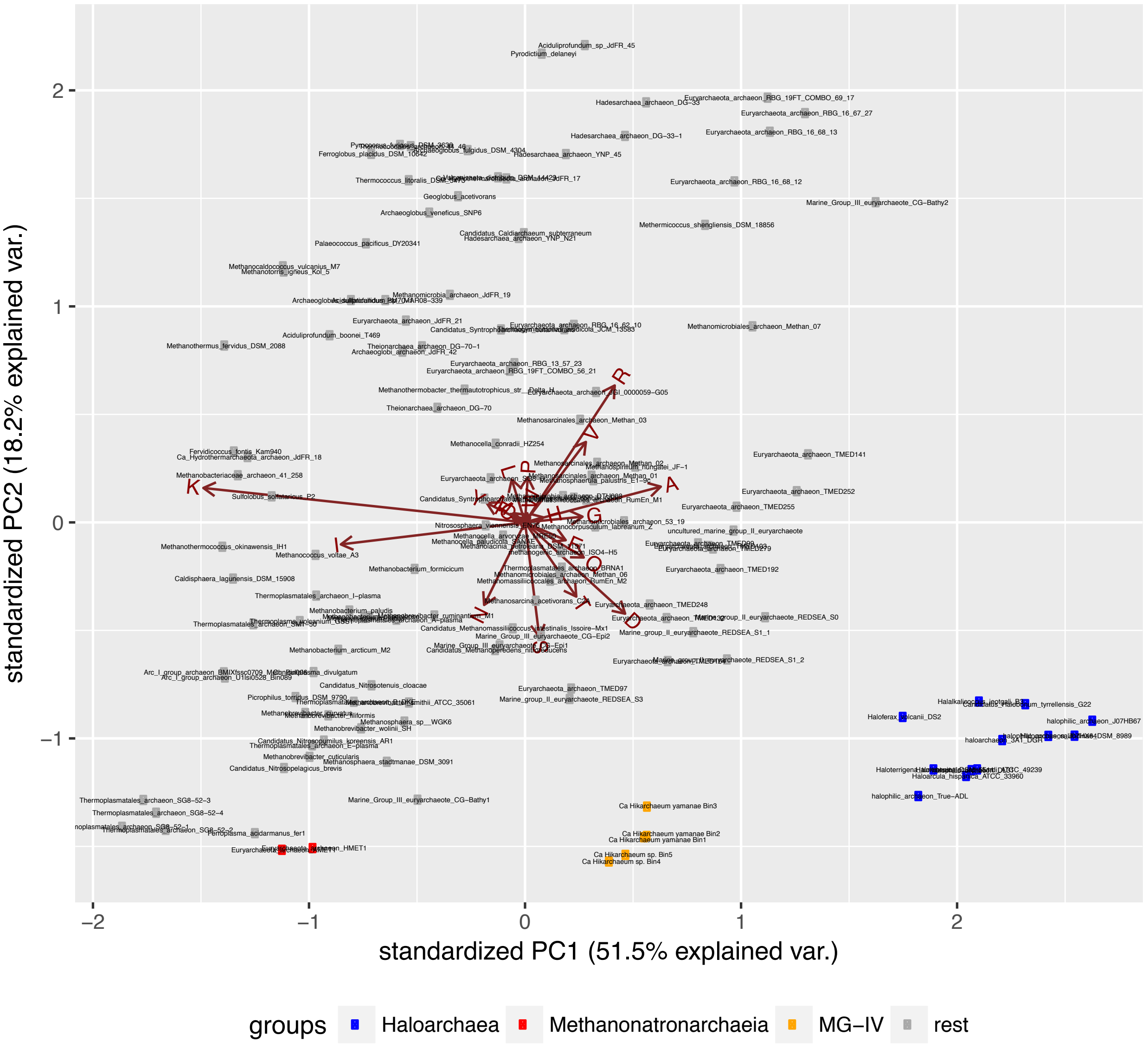

(Supplementary Figure 14a)

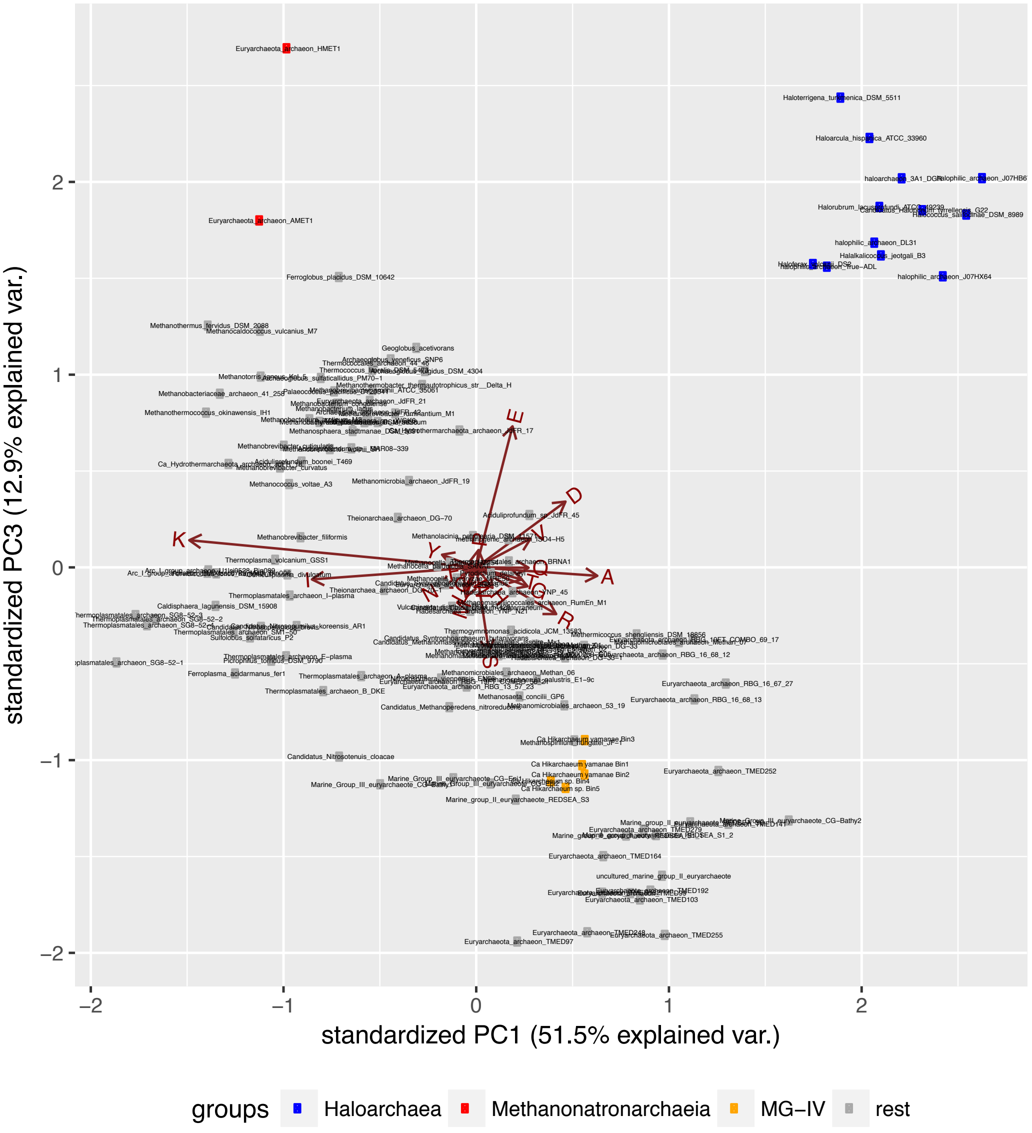

(Supplementary Figure 14b)

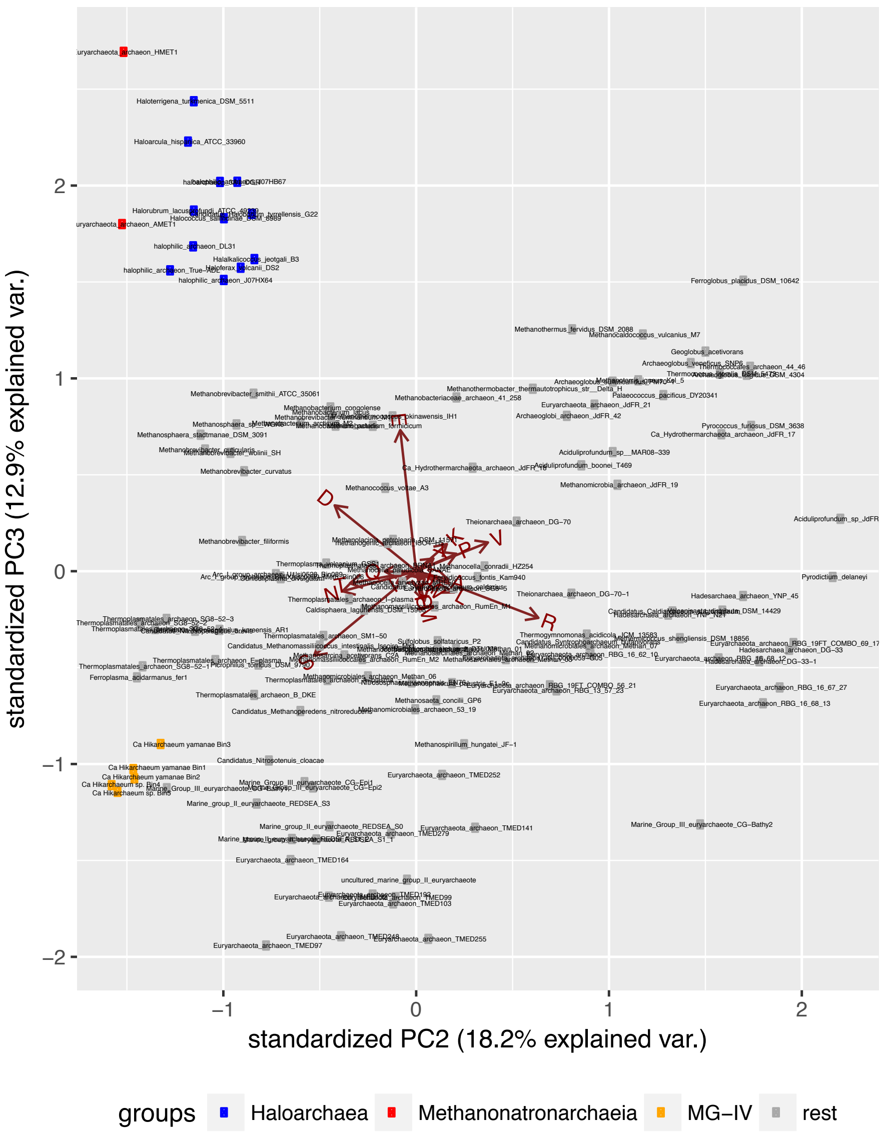

(Supplementary Figure 14c)

Supplementary Figure 14: Principal component analysis of amino acid compositions of the untreated 56 ribosomal protein supermatrix alignment. Axes correspond to a: PC1 and PC2, b: PC1 and PC3, c: PC2 and PC3. Per-taxon amino acid frequencies were calculated with AMAS and used as variables in a Principle component analysis. Biplots for the combinations of the first three principal components were created in R. The representative taxa from Haloarchaea (blue), Hikarchaea (MG-IV; yellow) and Methanonatronarchaea (red) are highlighted.

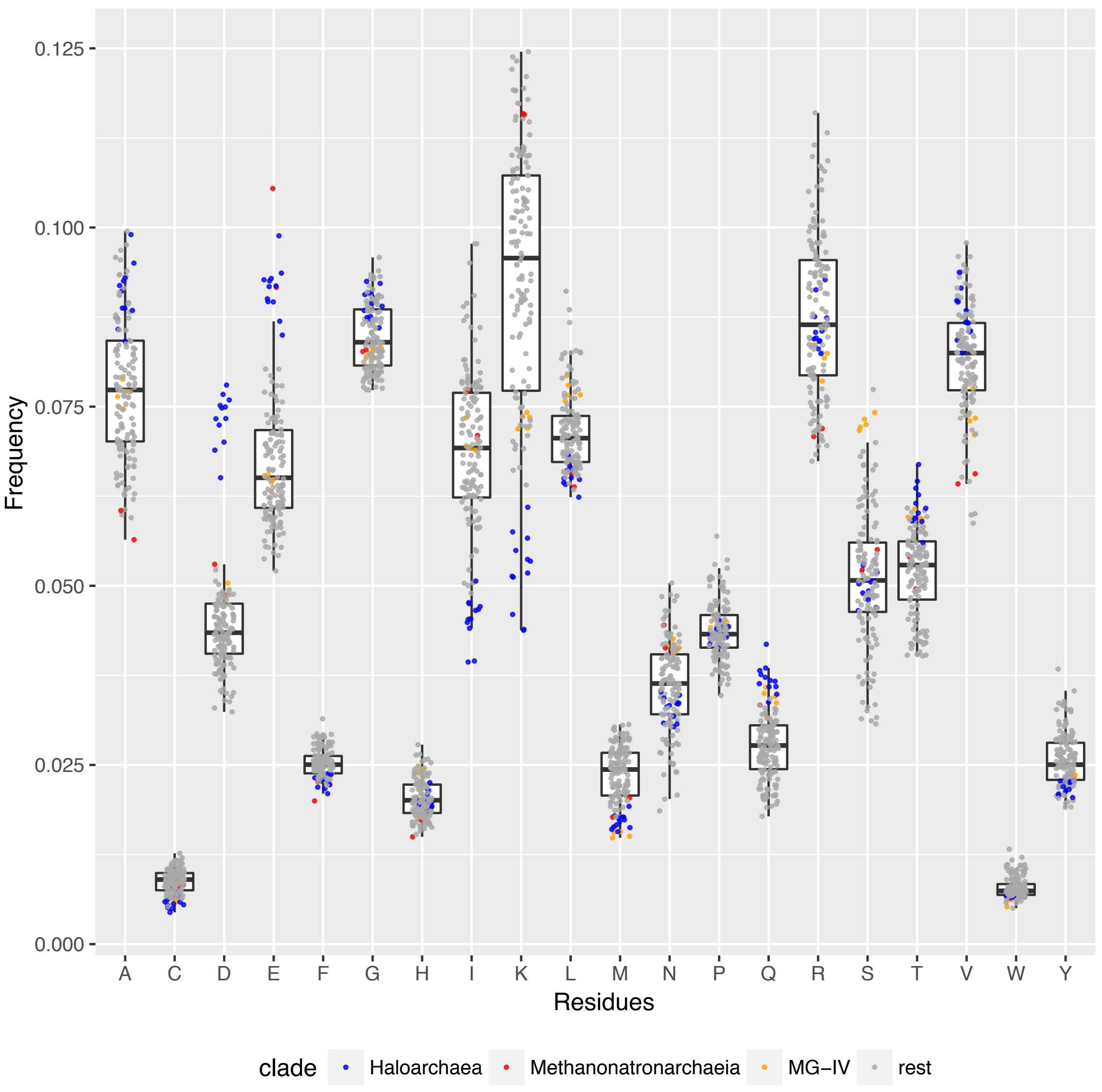

Supplementary Figure 15: Amino acid compositions of the untreated 56 ribosomal protein supermatrix alignment. Per-taxon amino acid frequencies were calculated with AMAS and visualized in in a box plot per amino acid. The representative taxa from Haloarchaea (blue), Hikarchaea (MG-IV; yellow) and Methanonatronarchaea (red) are highlighted.

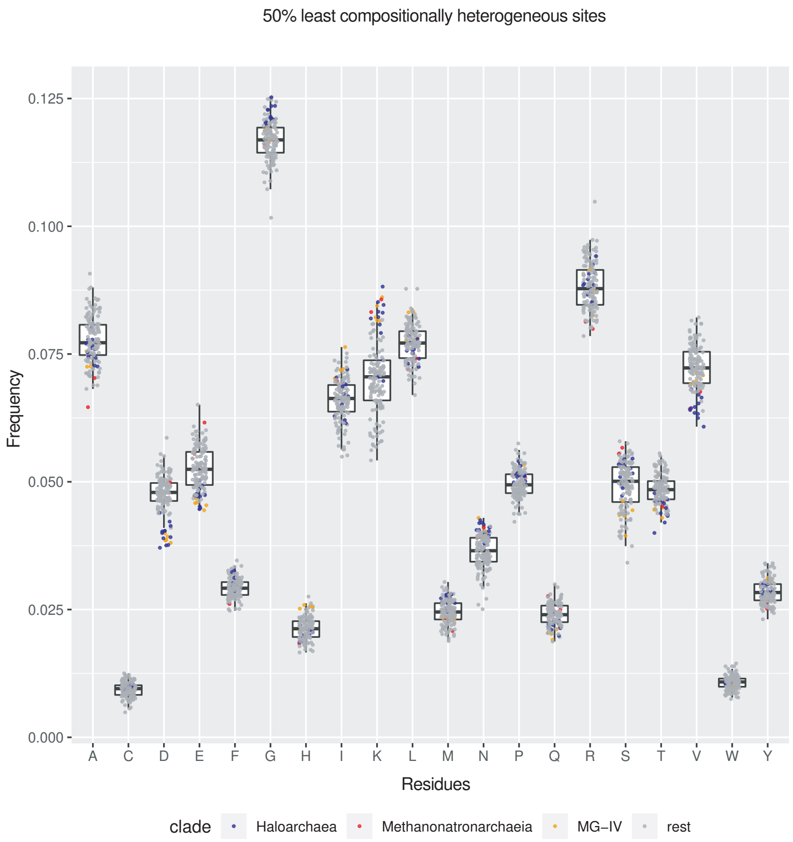

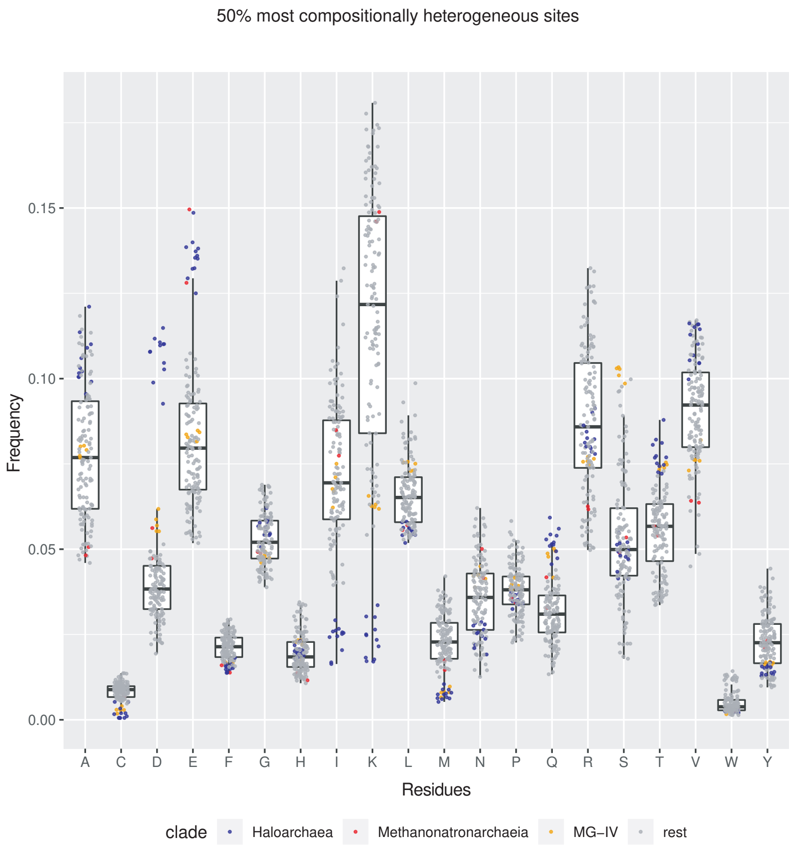

Supplementary Figure 16: Comparison of amino acid compositions of the 50% most heterogeneous sites versus the 50% least heterogeneous sites (χ^2-trimmed) of the 56 ribosomal protein supermatrix alignment. Per-taxon amino acid frequencies were calculated with AMAS and visualized in a box plot per amino acid. The 50% least heterogeneous sites correspond to the χ^2-trimmed alignment used for species tree reconstruction. The representative taxa from Haloarchaea (blue), Hikarchaeia (MG-IV; yellow) and Methanonatronarchaea (red) are highlighted. Acidic residues are aspartic acid (D) and glutamic acid (E)

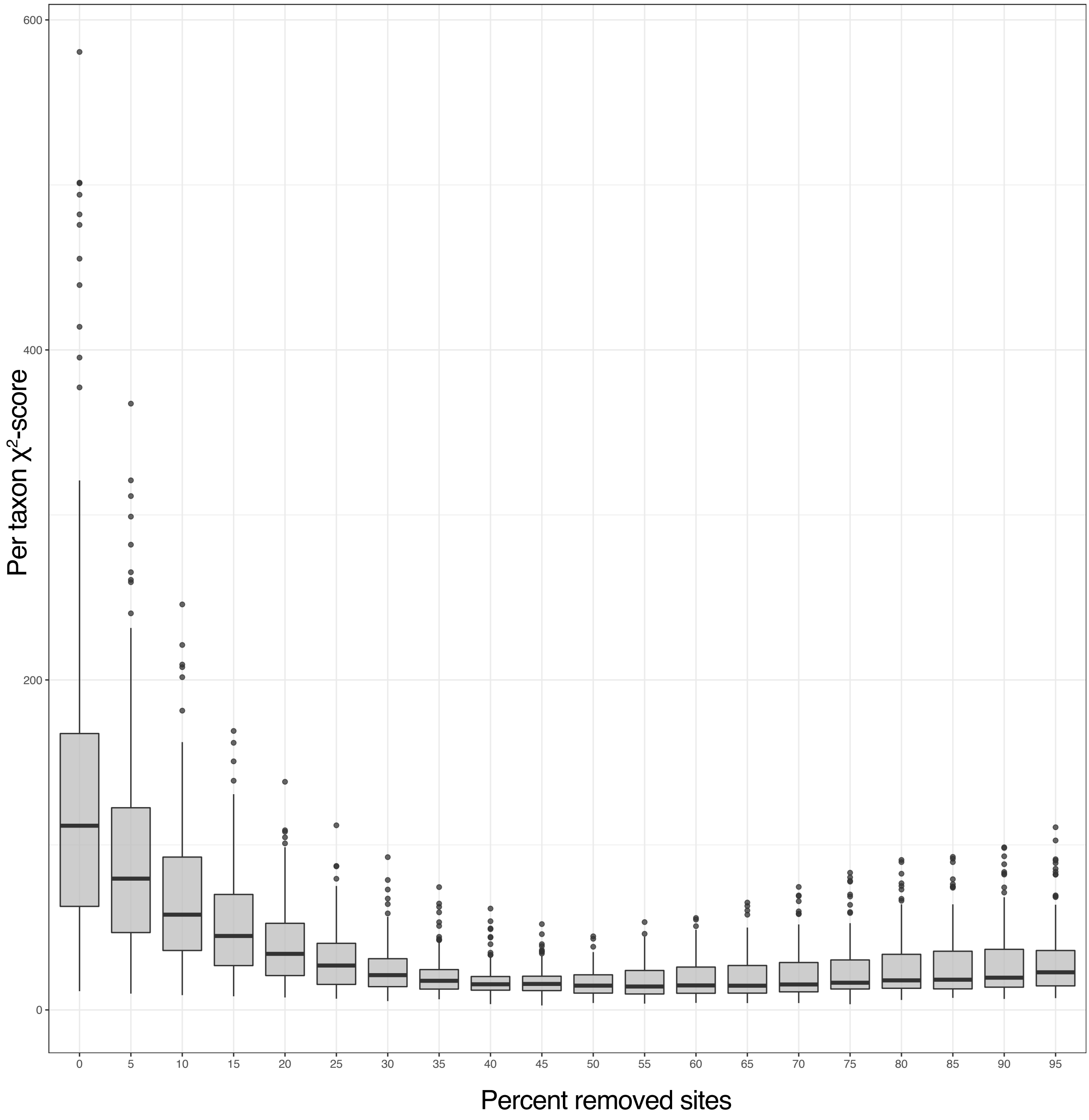

Supplementary Figure 17: Removal of most compositionally heterogeneous sites. Boxplot portraying the distribution of per-taxon χ^2-scores in the untreated supermatrix alignment based on 56 ribosomal proteins as the most heterogeneous sites are removed in steps of 5%. Each datapoint is one taxon.

euryarchaeota_TACK_56_arCOGs.noHaloMGIV.fasta.treefile.nms

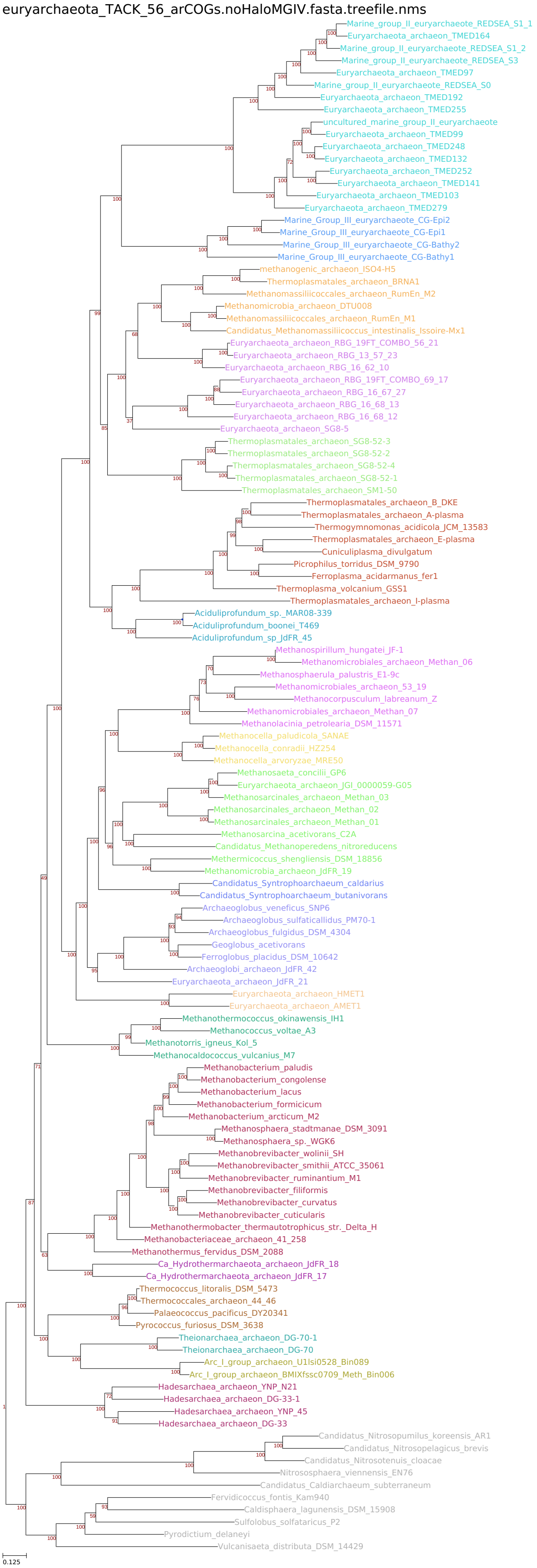

(Supplementary Figure 18a)

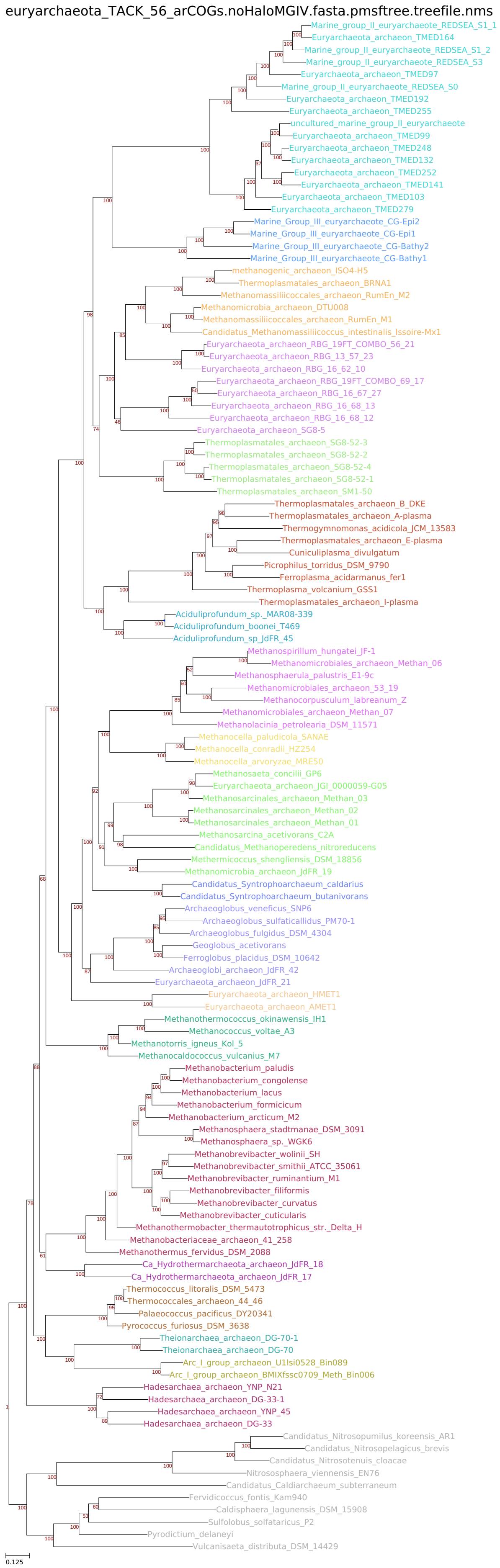

(Supplementary Figure 18b)

Supplementary Figure 18: Maximum likelihood phylogeny of Euryarchaeota based on the concatenated alignment of 56 ribosomal proteins excluding Haloarchaea and Hikarchaea. ML trees inferred under LG+C60+F+Γ4 with ultra-fast-bootstraps (a) and its PMSF approximation with non-parametric bootstraps (b) as implemented by IQTREE on the alignment derived from the concatenation of 56 ribosomal proteins across Euryarchaeota (Zaremba-Niedźwiedzka et al. 2017) excluding Haloarchaea and Hikarchaea.

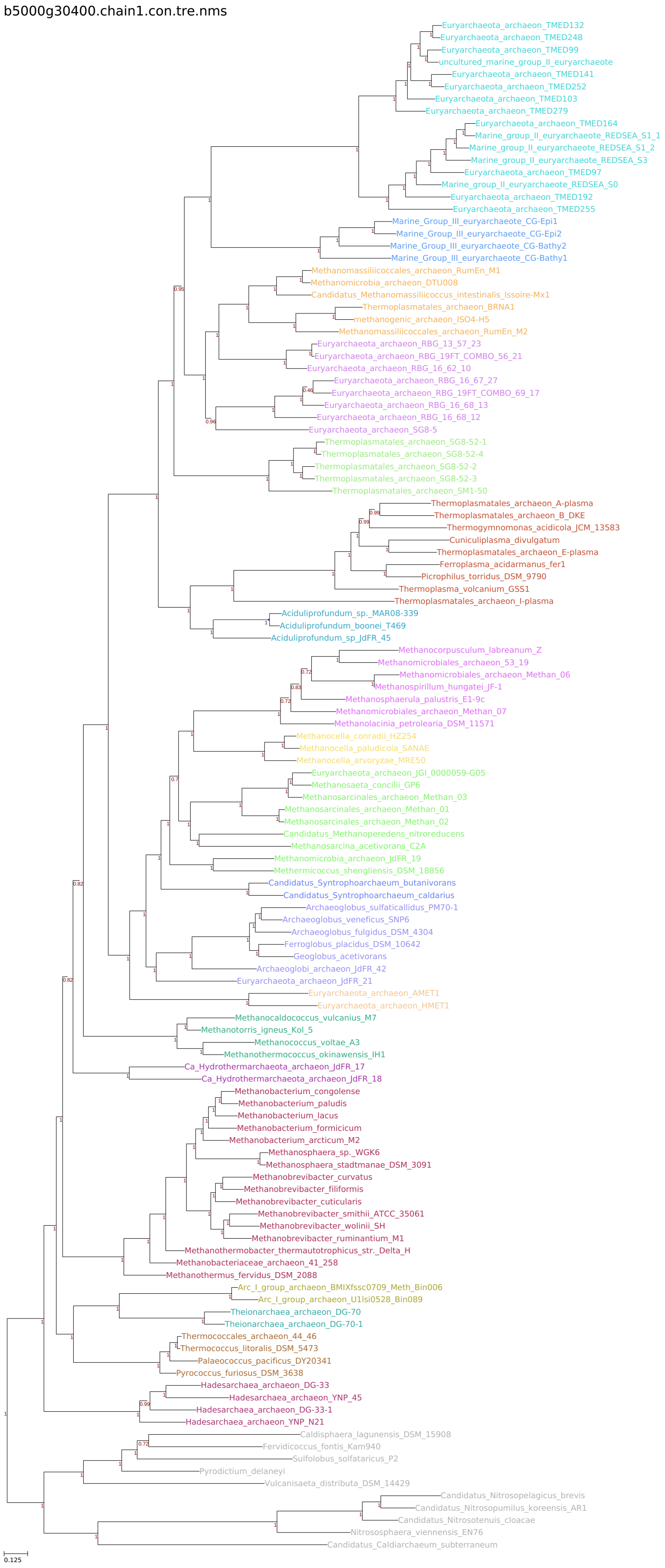

(Supplementary Figure 19a)

b5000g30400.chain2.con.tre.nms

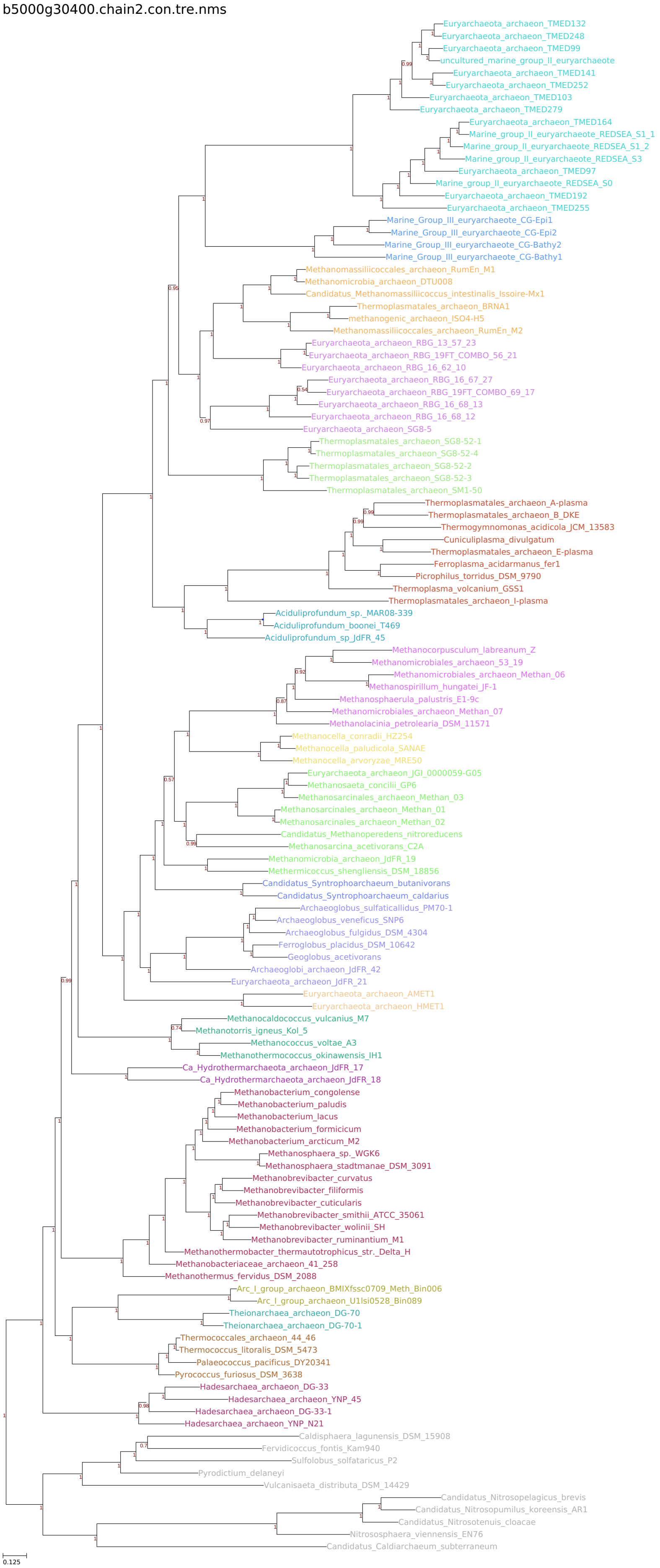

(Supplementary Figure 19b)

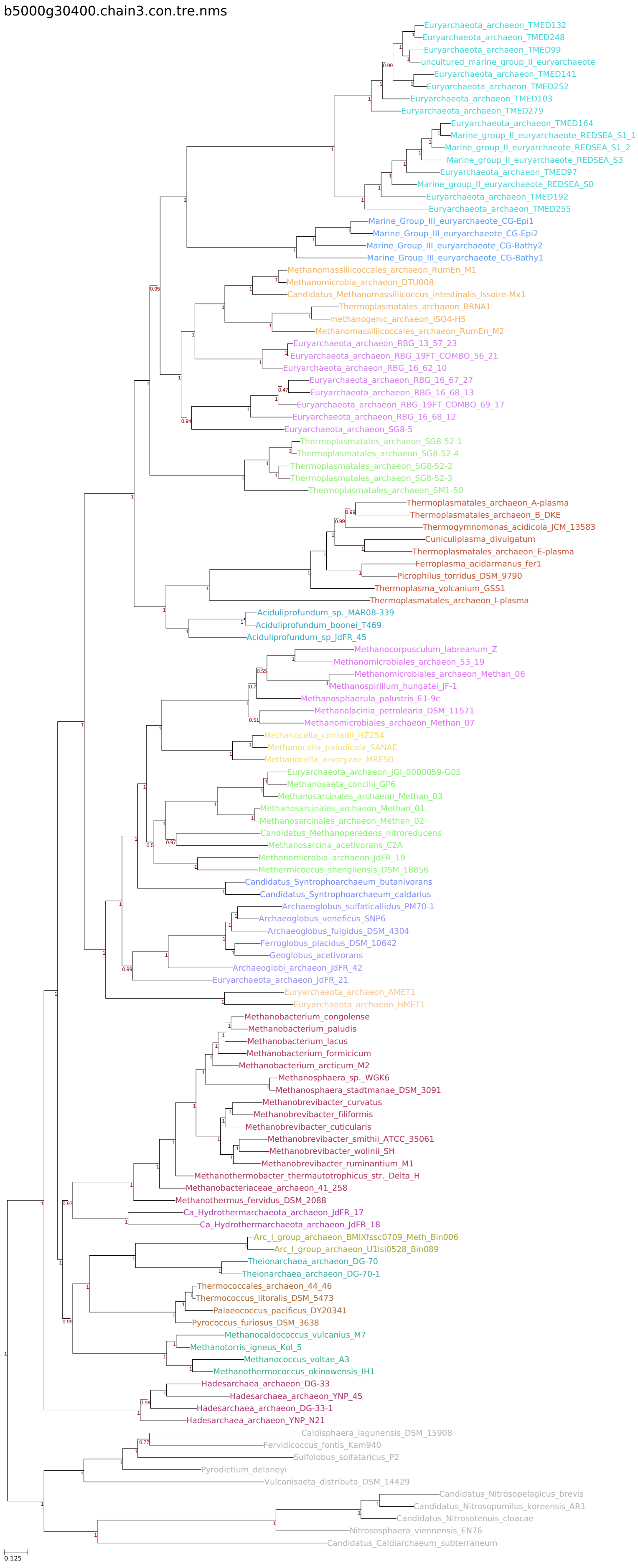

(Supplementary Figure 19c)

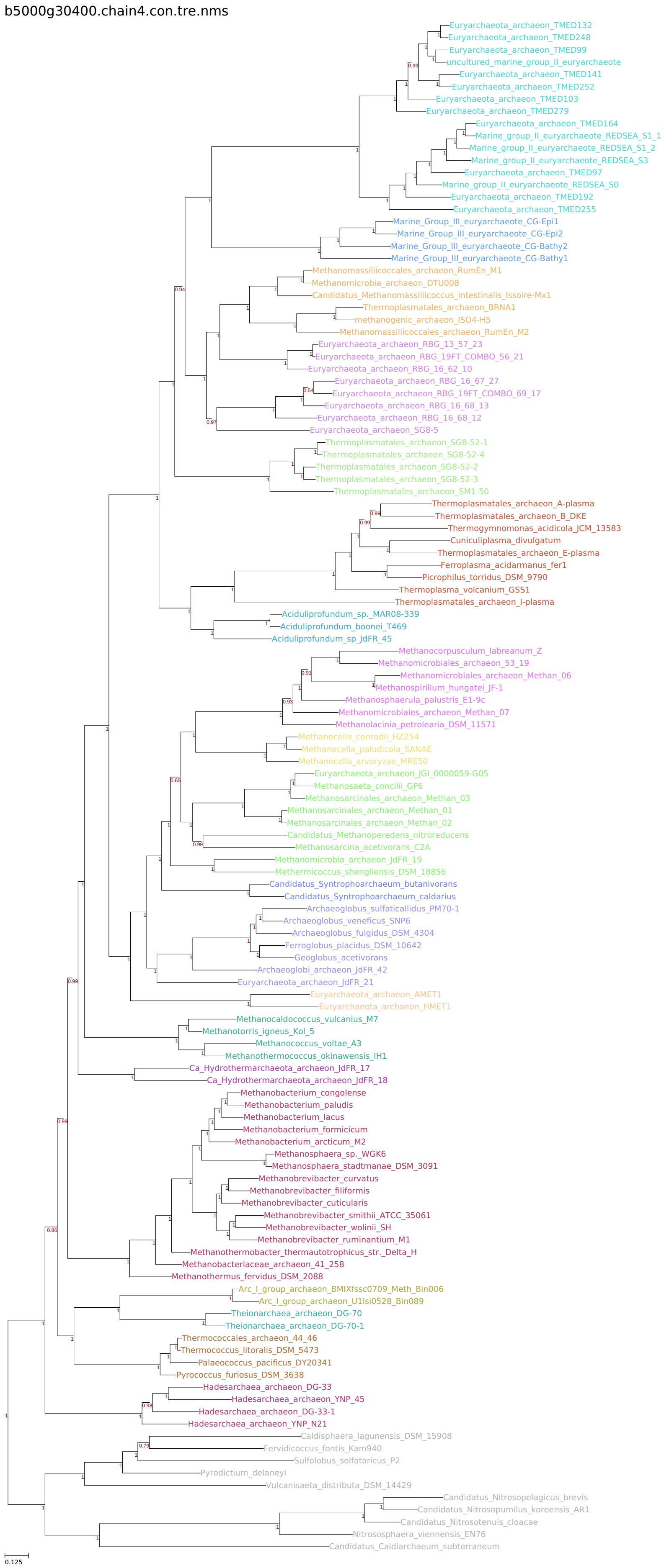

(Supplementary Figure 19d)

Supplementary Figure 19: Bayesian inference of Euryarchaeota phylogeny based on the concatenated alignment of 56 ribosomal proteins excluding Haloarchaea and Hikarcheae. Consensus trees of each of the 4 MCMC chains (a-d) that were inferred under CAT+GTR+Γ4 on the alignment derived from the concatenation of 56 ribosomal proteins across Euryarchaeota (Zaremba-Niedźwiedzka et al. 2017) from which all Haloarchaea and Hikarchaea were removed. Chains 1, 2 and 4 converged (maxdiff = 0.25).

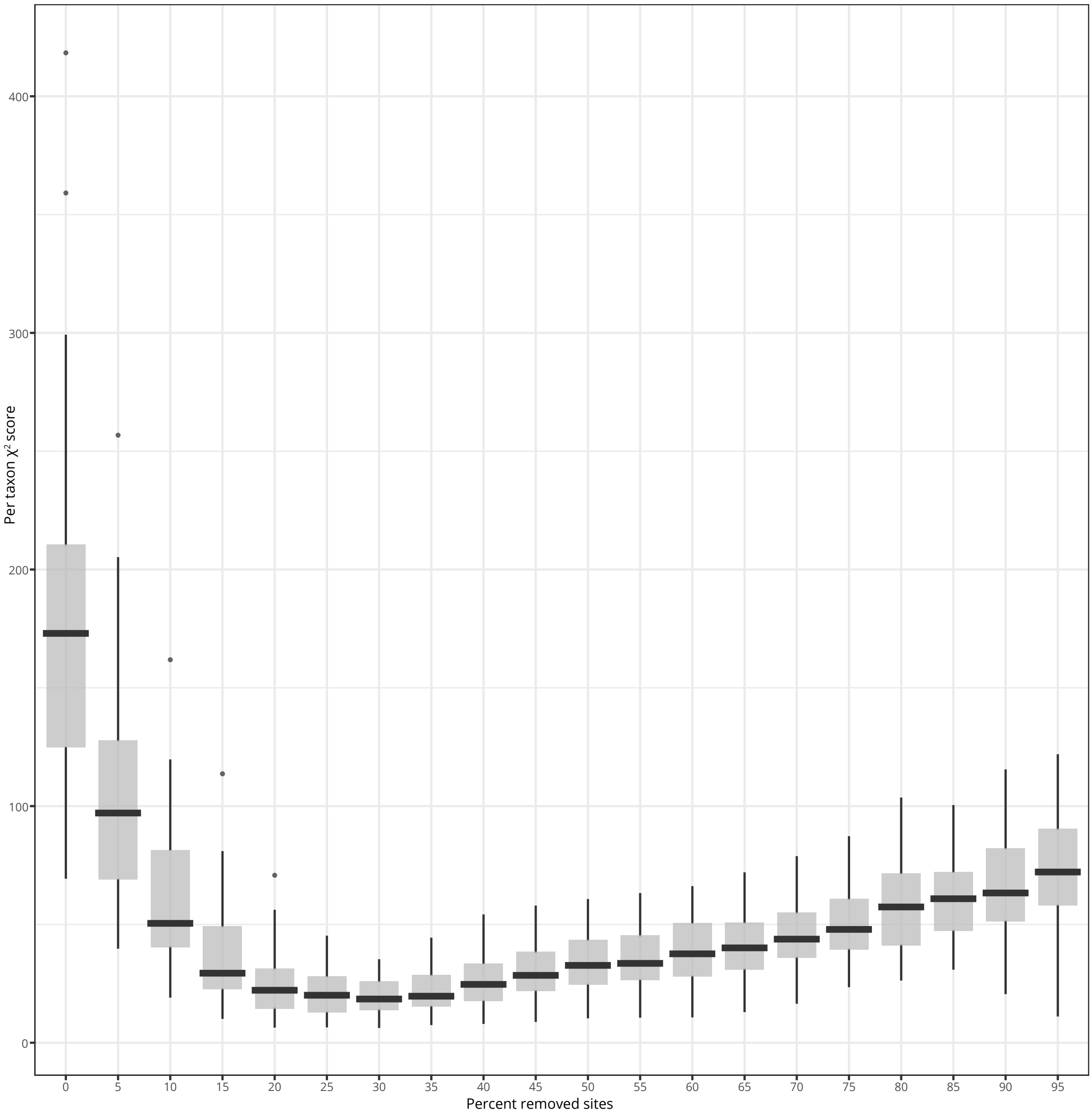

Supplementary Figure 20: Removal of most compositionally heterogeneous sites for the Methanotecta dataset with extended Haloarchaea sampling. Boxplot portraying the distribution of per-taxon χ^2-scores in the untreated supermatrix alignment based on 56 ribosomal proteins as the most heterogeneous sites are removed in steps of 5%. Each datapoint is one taxon.

MethHikHalo.noHalonotius.cct.f300.aln.treefile.reroot

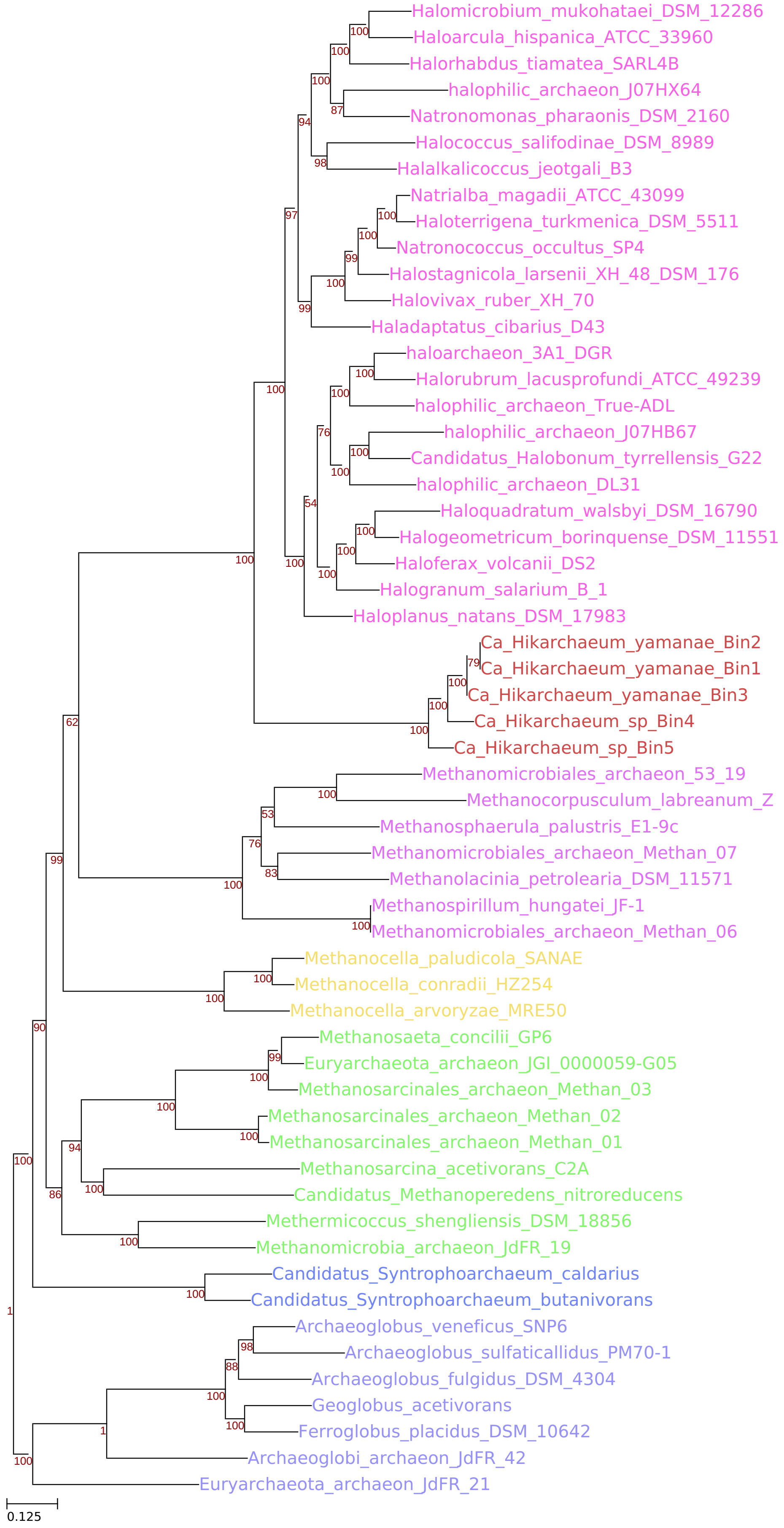

(Supplementary Figure 21a)

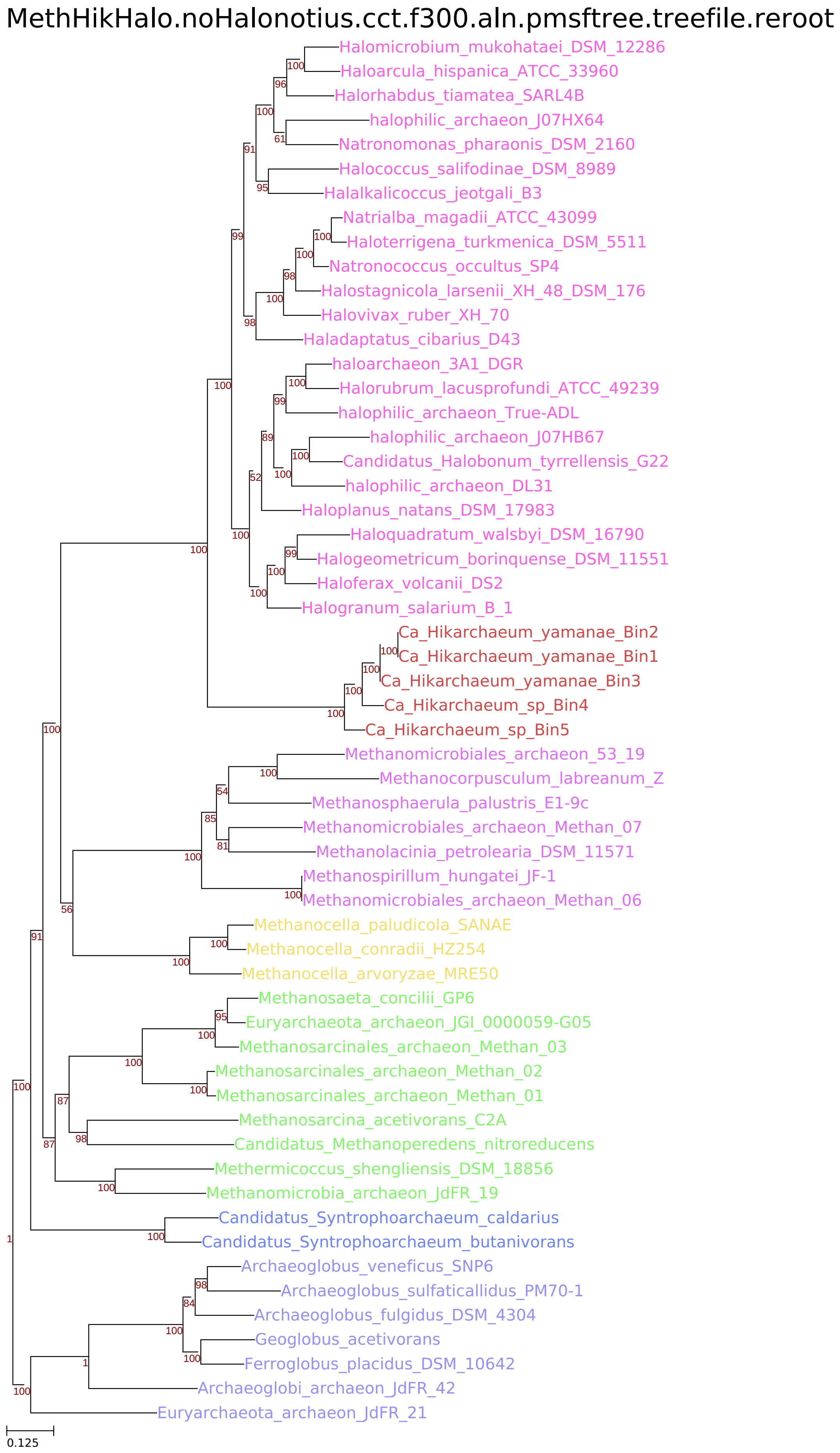

(Supplementary Figure 21b)

Supplementary Figure 21: Maximum likelihood phylogeny of Methanotecta based on the χ^2-trimmed (30% most heterogeneous sites removed) concatenated alignment of 56 ribosomal proteins with extended Haloarchaea sampling. ML trees inferred under LG+C60+F+Γ4 with ultra-fast-bootstraps (a) and its PMSF approximation with non-parametric bootstraps (b) as implemented by IQTREE on the alignment derived from the concatenation of 56 ribosomal proteins across Methanotecta (Zaremba-Niedźwiedzka et al. 2017).

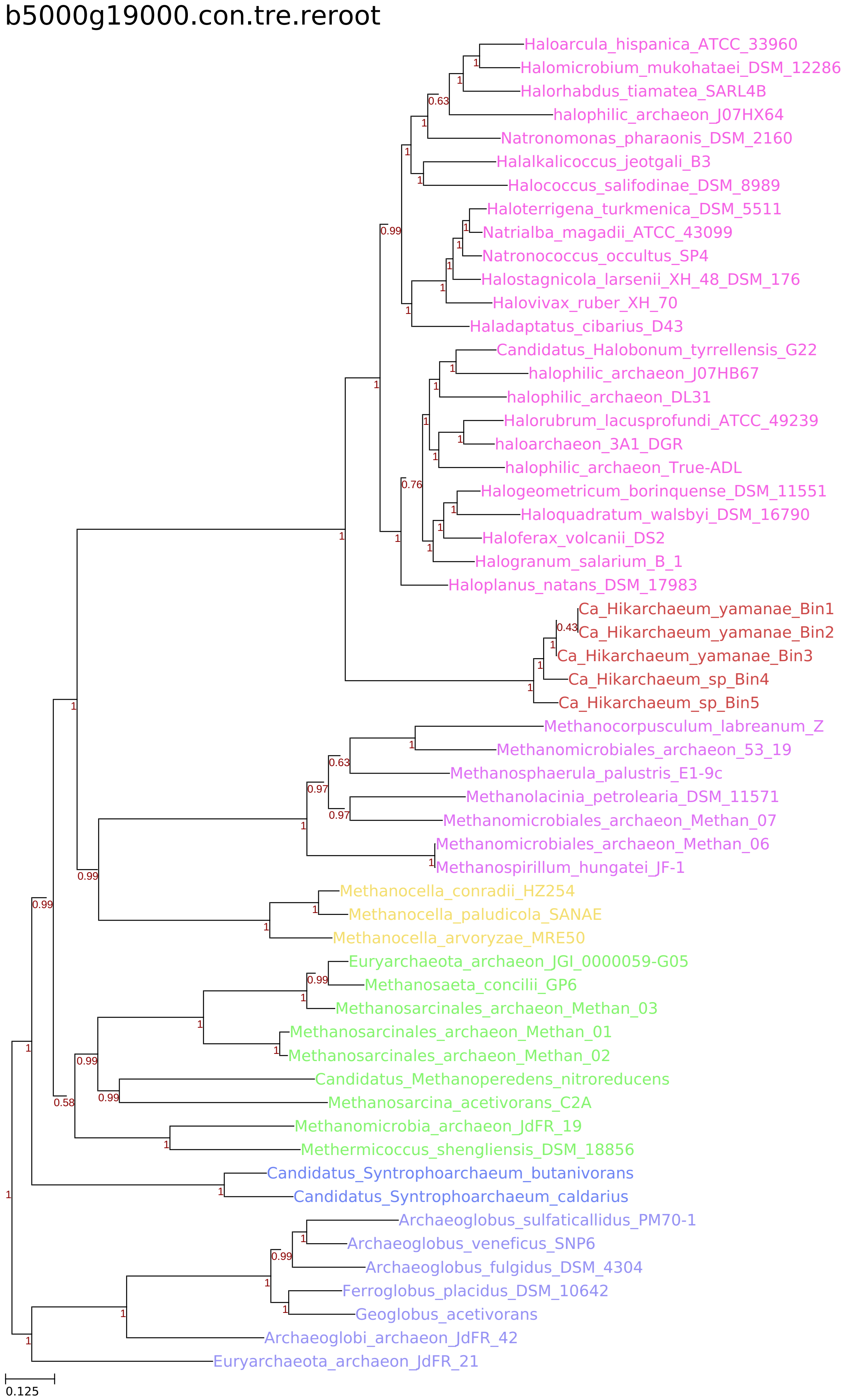

Supplementary Figure 22: Bayesian inference of Methanotecta phylogeny based on the χ^2-trimmed (30% most heterogeneous sites removed) concatenated alignment of 56 ribosomal proteins with extended Haloarchaea sampling. Consensus tree of 4 MCMC chains that were inferred under CAT+LG+Γ4 on the alignment derived from the concatenation of 56 ribosomal proteins across Methanotecta (Zaremba-Niedźwiedzka et al. 2017).

MethHikHalo.noHalonotius.cct.untr.aln.treefile.reroot

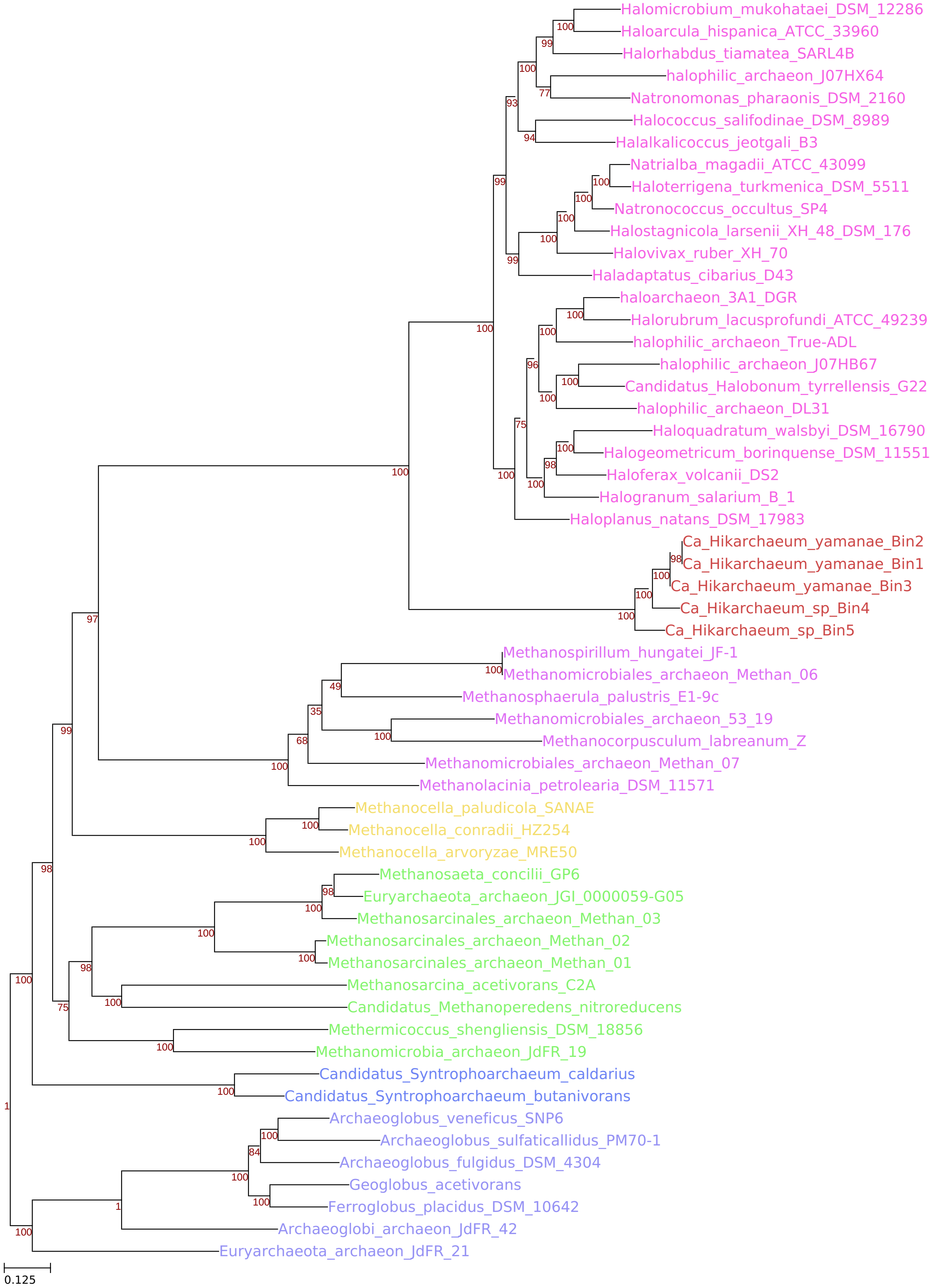

(Supplementary Figure 23a)

MethHikHalo.noHalonotius.cct.untr.aln.pmsftree.treefile.reroot

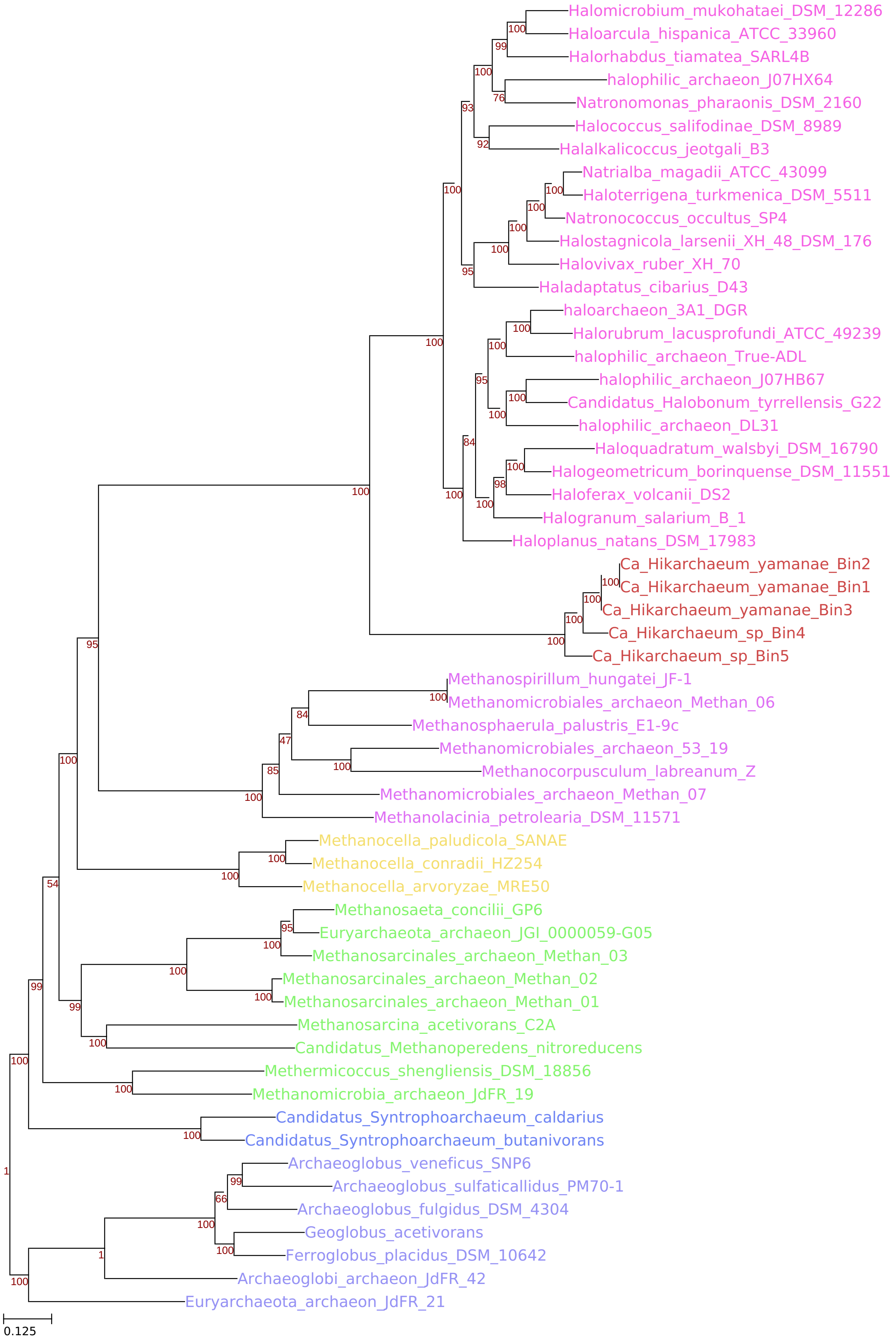

(Supplementary Figure 23b)

Supplementary Figure 23: Maximum likelihood phylogeny of Methanotecta based on the untreated concatenated alignment of 56 ribosomal proteins with extended Haloarchaea sampling. ML trees inferred under LG+C60+F+Γ4 with ultra-fast-bootstraps (a) and its PMSF approximation with non-parametric bootstraps (b) as implemented by IQTREE on the alignment derived from the concatenation of 56 ribosomal proteins across Methanotecta (Zaremba-Niedźwiedzka et al. 2017).

b5000g20800.con.tre.reroot

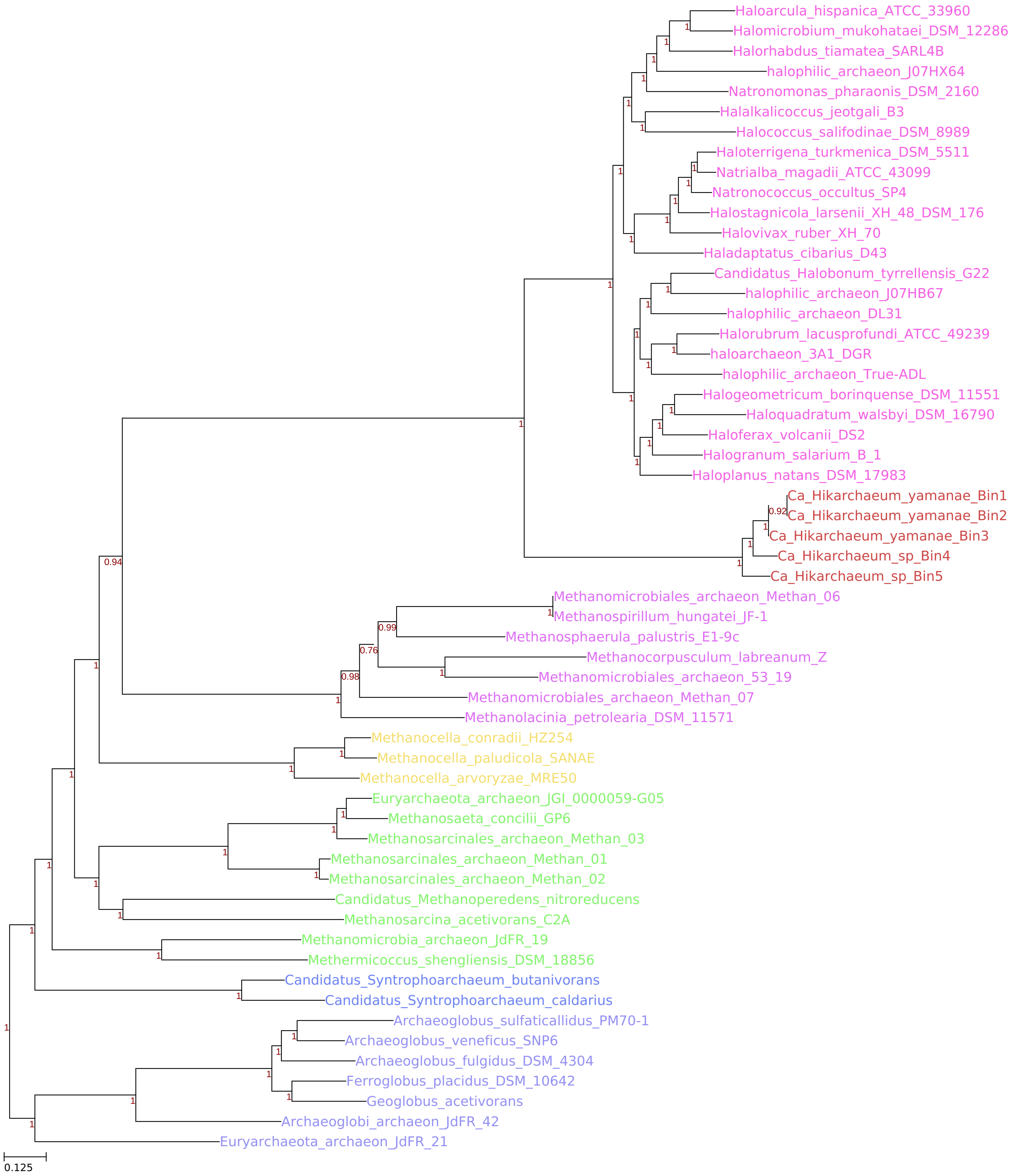

Supplementary Figure 24: Bayesian inference of Methanotecta phylogeny based on the untreated concatenated alignment of 56 ribosomal proteins with extended Haloarchaea sampling. Consensus tree of 4 MCMC chains that were inferred under CAT+LG+Γ4 on the alignment derived from the concatenation of 56 ribosomal proteins across Methanotecta (Zaremba-Niedźwiedzka et al. 2017).

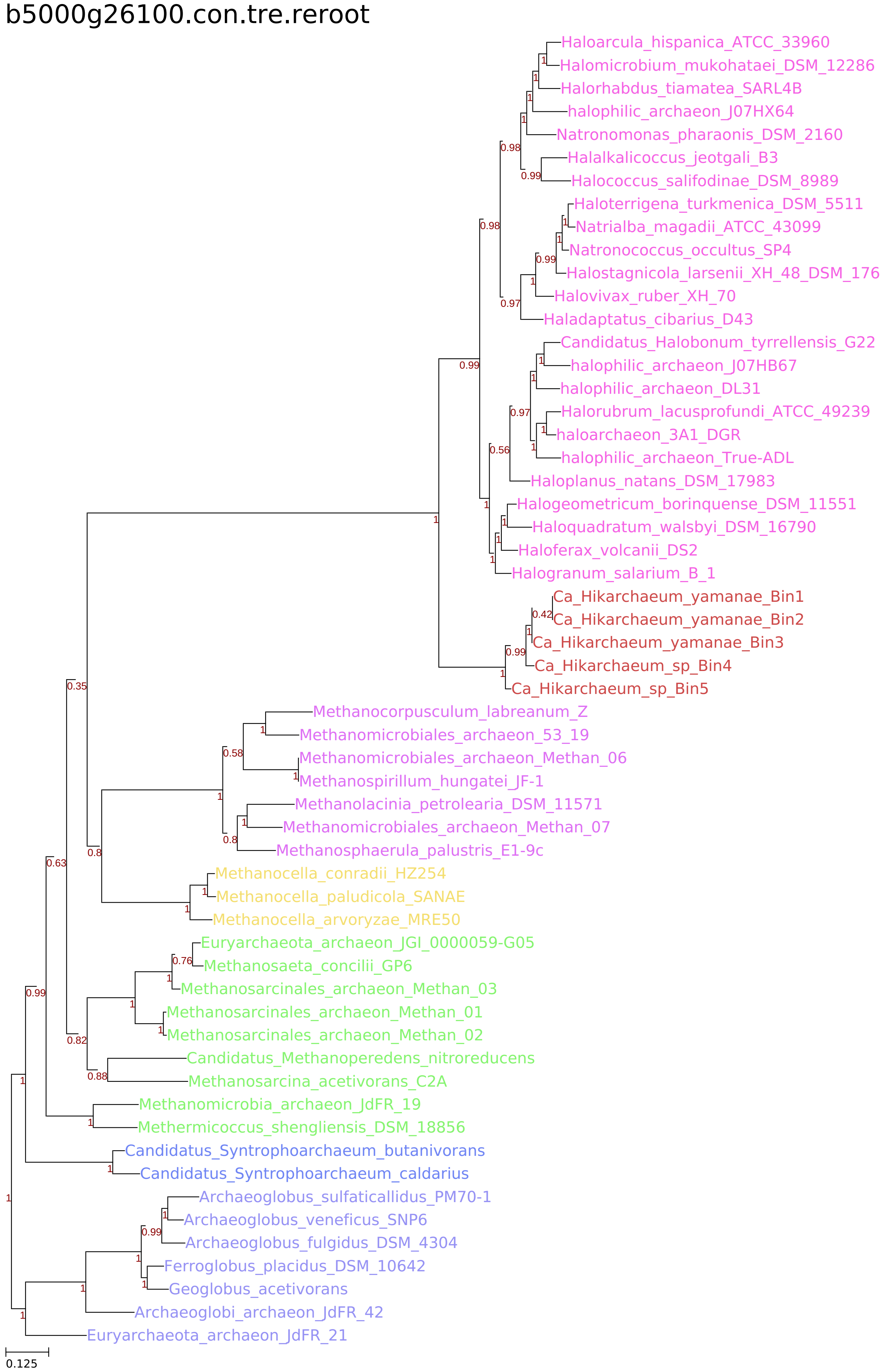

Supplementary Figure 25: Bayesian inference of Methanotecta phylogeny based on the SR4-recoded concatenated alignment of 56 ribosomal proteins with extended Haloarchaea sampling. Consensus tree of 4 MCMC chains that were inferred under CAT+GTR+Γ4 on the alignment derived from the concatenation of 56 ribosomal proteins across Methanotecta (Zaremba-Niedźwiedzka et al. 2017).

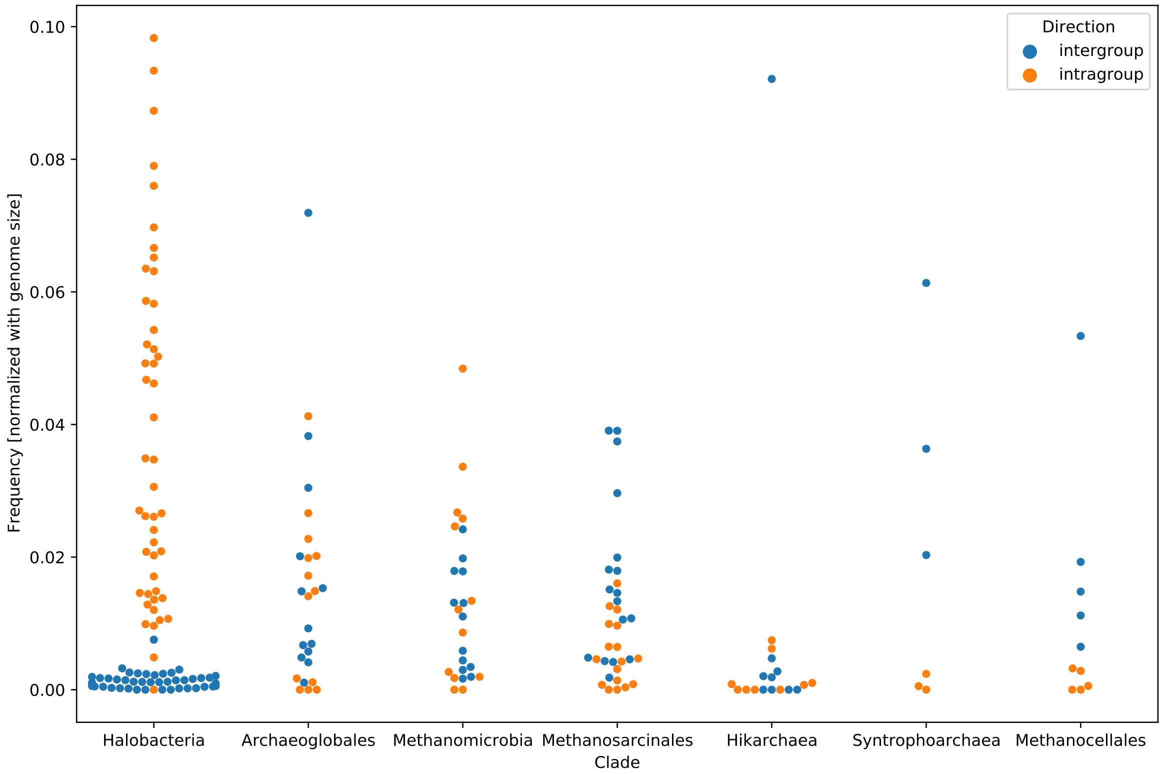

Supplementary Figure 26: Frequency of incoming transfers for main groups of the species tree. For each leaf and ancestral node (excluding the last common ancestor of the group) the number of transfers, as inferred with ALE, from other members of the same group (orange) and from other nodes in the species tree (blue) were normalized by the number of genes in the (inferred) genome. Haloarchaea show significantly more intra- than intergroup transfers (p=6.7e-13), whereas other groups show no significant difference or even a reverse pattern (Methanosarcinales, p=0.002).

Wood Ljungdahl Pathway, Methanogenesis and associated hydrogenases

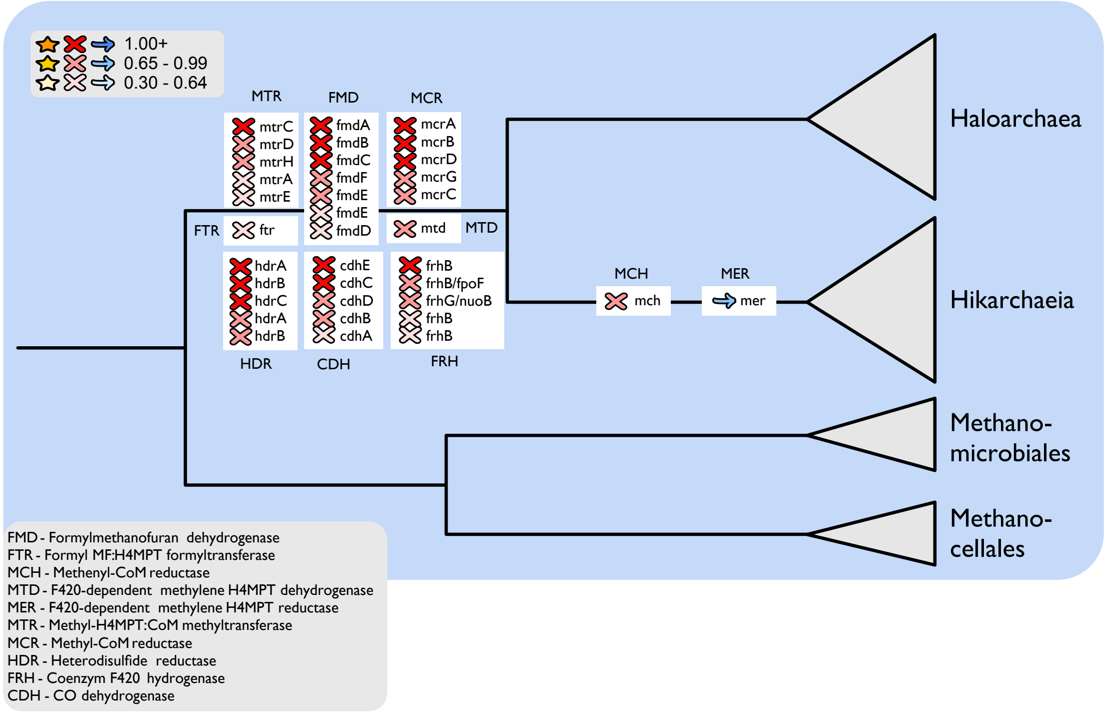

Aerobic respiration

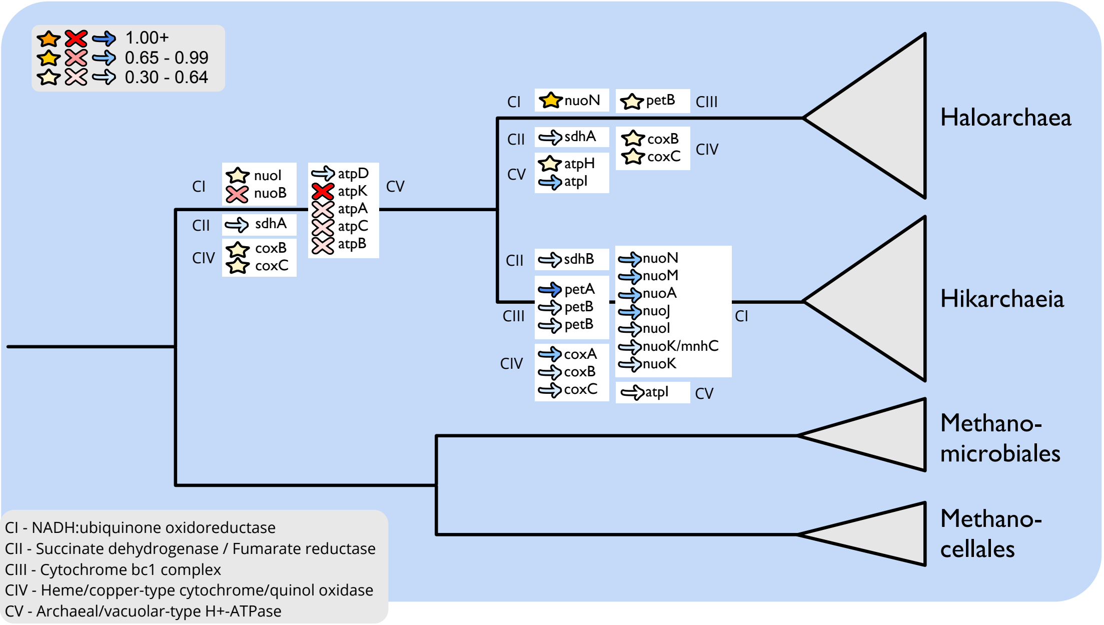

Supplementary Figure 27: Detailed gene flow diagrams for gene families related to methanogenesis and respiration. Stars, crosses and arrows denote originations, losses and transfers (OLT), respectively. Intensities of these symbols reflect the observed OLT frequencies in the ALE reconciliations. Gene families that are distinct but map to the same overarching COG (e.g. fmdD, fmdE, coxB, coxC) are flagged with unique numbers.

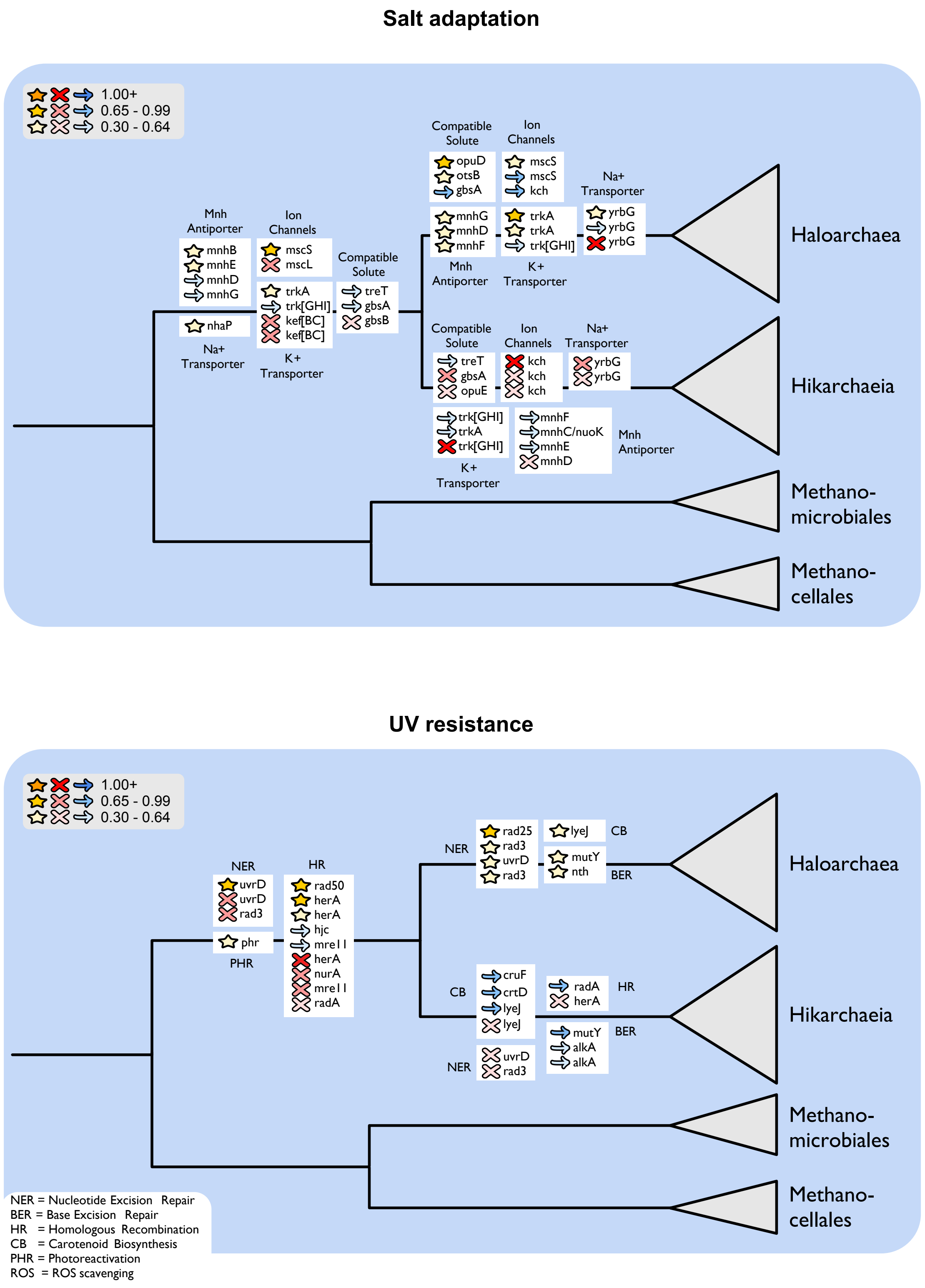

Supplementary Figure 28: Detailed gene flow diagrams for gene families related to salt adaptation and UV resistance. Stars, crosses and arrows denote originations, losses and transfers (OLT), respectively. Intensities of these symbols reflect the observed OLT frequencies in the ALE reconciliations. Gene families that are distinct but map to the same overarching COG (e.g. mnhB, mnhC, uvrD, crtD) are flagged with unique numbers.

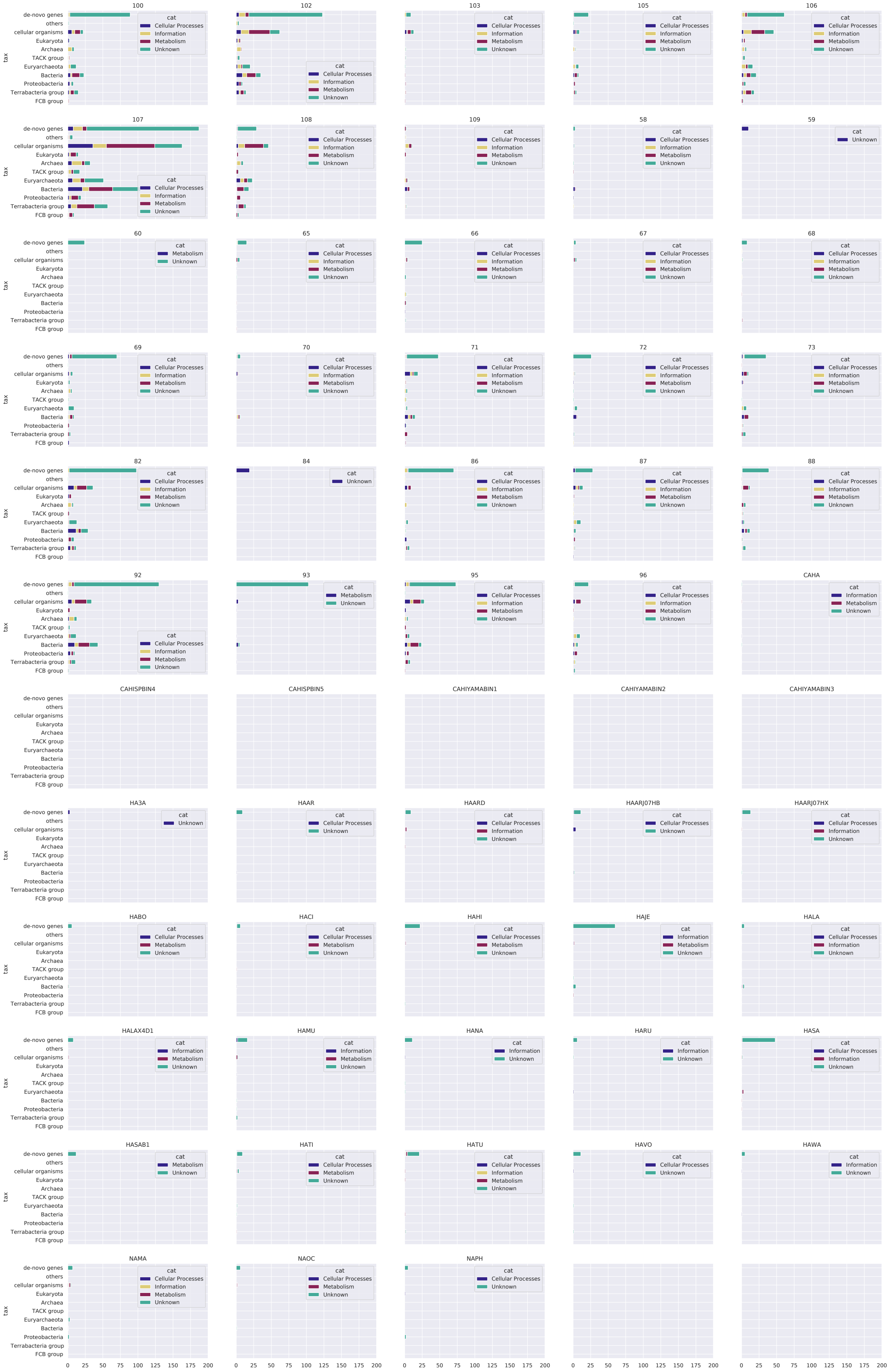

Supplementary Figure 29: Tracing the origins of gene acquisitions. Stacked bar charts showing the inferred taxonomic affiliation of originations at internal and terminal leaves in the Haloarchaea and Hikarachaea. Each bar corresponds to an inferred taxonomic group, where the length of the bar indicates the number of clusters with that taxonomic annotation and the colors indicate the broad functional annotation of the clusters. For visualization purposes, we also summarized more specific placements into broader taxonomic groups. See Supplementary Table S12 for more detailed placement results.

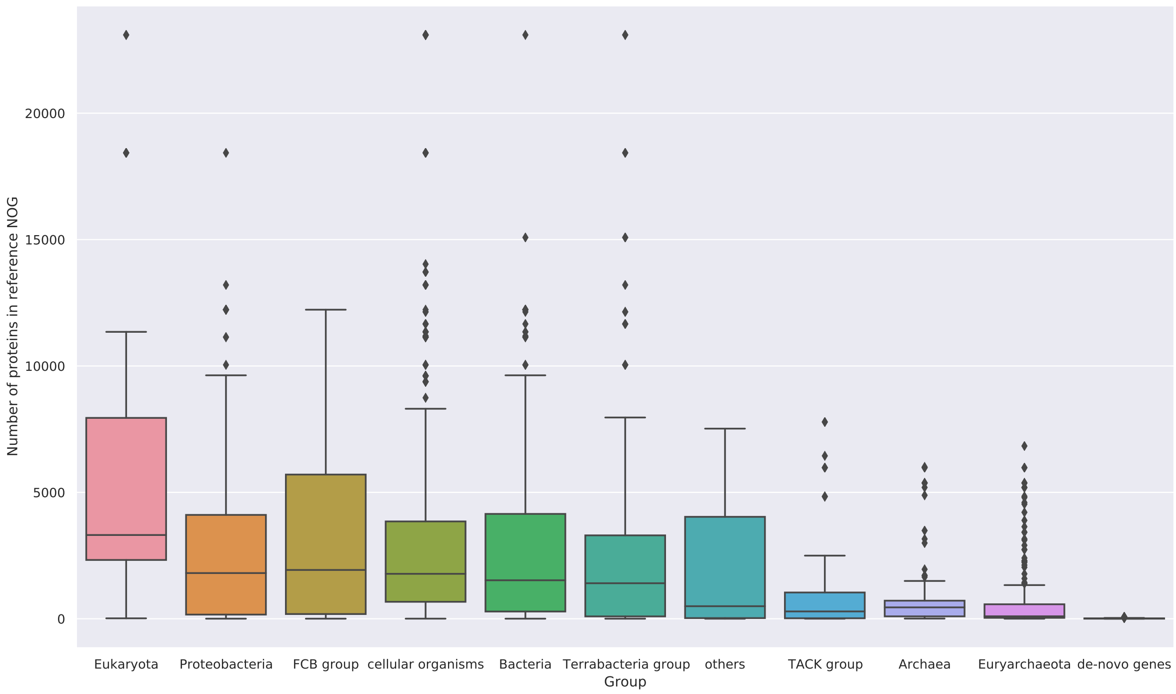

Supplementary Figure 30: Reference family size distribution for different taxonomic affiliations. For each origination with an inferred taxonomic affiliation, the number of genes in the reference NOG gene family was compiled and grouped by taxonomic label. Boxplots for each group are shown. Gene family size was significantly larger in those cases where 'Eukaryota' were inferred as the taxonomic label compared to when any other label was inferred (p=2.4e-7).
