# Supplementary material for: Hikarchaeia demonstrate an intermediate stage in the methanogen-to-halophile transition
Source: Nat Commun. 2020 Oct 30;11:5490. doi: 10.1038/s41467-020-19200-2 (PMC7599335; doi:10.1038/s41467-020-19200-2)
Supplement: Supplementary file 2 — Reporting Summary [file 41467_2020_19200_MOESM2_ESM.pdf]

nature research

Corresponding author(s): Joran Martijn

Last updated by author(s): Jul 10, 2020

Reporting Summary

Nature Research wishes to improve the reproducibility of the work that we publish. This form provides structure for consistency and transparency in reporting. For further information on Nature Research policies, see our Editorial Policies and the Editorial Policy Checklist.

Statistics

For all statistical analyses, confirm that the following items are present in the figure legend, table legend, main text, or Methods section.

n/a	Confirmed	
☒	☐	The exact sample size (*n*) for each experimental group/condition, given as a discrete number and unit of measurement
☒	☐	A statement on whether measurements were taken from distinct samples or whether the same sample was measured repeatedly
☐	☒	The statistical test(s) used AND whether they are one- or two-sided<br>*Only common tests should be described solely by name; describe more complex techniques in the Methods section.*
☒	☐	A description of all covariates tested
☒	☐	A description of any assumptions or corrections, such as tests of normality and adjustment for multiple comparisons
☒	☐	A full description of the statistical parameters including central tendency (e.g. means) or other basic estimates (e.g. regression coefficient) AND variation (e.g. standard deviation) or associated estimates of uncertainty (e.g. confidence intervals)
☒	☐	For null hypothesis testing, the test statistic (e.g. *F*, *t*, *r*) with confidence intervals, effect sizes, degrees of freedom and *P* value noted<br>*Give P values as exact values whenever suitable.*
☒	☐	For Bayesian analysis, information on the choice of priors and Markov chain Monte Carlo settings
☒	☐	For hierarchical and complex designs, identification of the appropriate level for tests and full reporting of outcomes
☒	☐	Estimates of effect sizes (e.g. Cohen's *d*, Pearson's *r*), indicating how they were calculated

Our web collection on statistics for biologists contains articles on many of the points above.

Software and code

Policy information about availability of computer code

Data collection	No software was used to collect data
Data analysis	All the software used for data analysis were open source and are described in detail in the Methods section of the manuscript

For manuscripts utilizing custom algorithms or software that are central to the research but not yet described in published literature, software must be made available to editors and reviewers. We strongly encourage code deposition in a community repository (e.g. GitHub). See the Nature Research guidelines for submitting code & software for further information.

Data

Policy information about availability of data

All manuscripts must include a data availability statement. This statement should provide the following information, where applicable:

- Accession codes, unique identifiers, or web links for publicly available datasets
- A list of figures that have associated raw data
- A description of any restrictions on data availability

The genome bins described in this study have been deposited at DDBJ/EMBL/GenBank under the BioProject ID PRJNA588249. The metagenome assemblies (https://doi.org/10.6084/m9.figshare.11117894.v1), phylogenomics dataset and supermatrix alignments (https://doi.org/10.6084/m9.figshare.11118164.v1), and unaligned sequences for gene families with less than four members, ultra-fast bootstrap trees for gene families with at least four members and ALE inferred gene family histories (https://doi.org/10.6084/m9.figshare.11352140) are available on FigShare.

Field-specific reporting

Please select the one below that is the best fit for your research. If you are not sure, read the appropriate sections before making your selection.

☐ Life sciences ☐ Behavioural & social sciences ☒ Ecological, evolutionary & environmental sciences

For a reference copy of the document with all sections, see nature.com/documents/nr-reporting-summary-flat.pdf

Ecological, evolutionary & environmental sciences study design

All studies must disclose on these points even when the disclosure is negative.

Study description	The study reconstructs draft genomes of Marine Group IV archaea and integrates them in an ancestral reconstruction analysis to study the evolutionary history of the Haloarchaea
Research sample	Does not apply
Sampling strategy	Does not apply
Data collection	The study collected publicly available sequence data from the Tara Oceans initiative to reconstruct the genomes of the Marine Group IV archaea, and publicly available genomes of Euryarchaeota representatives from Genbank
Timing and spatial scale	Does not apply
Data exclusions	Does not apply
Reproducibility	All methods to reproduce the manuscript's results are described in detail in the manuscript
Randomization	Does not apply
Blinding	Does not apply

Did the study involve field work? ☐ Yes ☒ No

Reporting for specific materials, systems and methods

We require information from authors about some types of materials, experimental systems and methods used in many studies. Here, indicate whether each material, system or method listed is relevant to your study. If you are not sure if a list item applies to your research, read the appropriate section before selecting a response.

Materials & experimental systems

n/a	Involved in the study
☒	☐ Antibodies
☒	☐ Eukaryotic cell lines
☒	☐ Palaeontology and archaeology
☒	☐ Animals and other organisms
☒	☐ Human research participants
☒	☐ Clinical data
☒	☐ Dual use research of concern

Methods

n/a	Involved in the study
☒	☐ ChIP-seq
☒	☐ Flow cytometry
☒	☐ MRI-based neuroimaging
