# Supplementary material for: Hikarchaeia demonstrate an intermediate stage in the methanogen-to-halophile transition
Source: Nat Commun. 2020 Oct 30;11:5490. doi: 10.1038/s41467-020-19200-2 (PMC7599335; doi:10.1038/s41467-020-19200-2)
Supplement: Supplementary file 3 — Description of Additional Supplementary Files [file 41467_2020_19200_MOESM3_ESM.pdf]

Description of Additional Supplementary Files

Supplementary Data 1: Proteomes used for phylogenomics datasets and gene family clustering. Taxa included in the species tree including taxonomy, assembly characteristics and source database are listed. Taxa of the Methanotecta that were used for gene family clustering and ancestral reconstruction are marked with 'ingroup'. The additional 12 genomes of haloarchaeal taxa are marked with ´added´.

Supplementary Data 2: Pervasive phylogenetic artefact MNA+HA+HIK in single gene trees. Percent bootstrap support for the grouping of Haloarchaea, Hikarchaea and Methanonatronarchaeia in the maximum likelihood tree of the single gene phylogenies of the 56 ribosomal proteins.

Supplementary Data 3: Transfers inferred by gene tree-species tree reconciliation. Number of transfers between all pairs of nodes in the Methanotecta with a frequency of >= 0.3 in the reconciliations. See Supplementary Figure S19 for a visualization of the results. Transfers labelled as coming from ancestral nodes generally represent transfers from unsampled or extinct child lineages of that node.

Supplementary Data 4: Inferred copy numbers and annotations of gene families putatively associated with methanogenesis, aerobic respiration, salt adaptation, UV resistance, rhodopsin and other metabolic pathways in the LMHHCA, LHHCA, LHiCA and LHaCA. For some gene families it is unclear whether they encode subunits of NADH dehydrogenase, Mnh Na+/H+ antiporter, coenzyme F420 reducing hydrogenase or [Ni-Fe]-hydrogenases. These families are listed in grey.

Supplementary Data 5: Gene flow of gene families of interest along the methanogen-to-halophile transition. Weakly (0.30-0.65), moderately (0.65-1.00) and strongly (1.00+) supported events or copy numbers are highlighted with increasing color intensity

Supplementary Data 6: Annotations of selected genes from Hikarachaeia genomes and pathways of interest. Annotations include (if available) PROKKA, arCOG, KEGG, EurNOG, Pfam, Cazy, TCDB, TIGRFAM, IPR domains and blast hits against ncbi's NR.

Supplementary Data 7: Annotations of all genes from Hikarachaeia genomes. Annotations include (if available) PROKKA, arCOG, KEGG, EurNOG, Pfam, Cazy, TCDB, TIGRFAM, IPR domains and blast hits against ncbi's NR.

Supplementary Data 8:Phylogenetic placements and taxonomic annotations for gene families gained by Haloarchaea and Hikarchaea. Gene clusters inferred as originations on any branch leading to and after the LHHCA. All sequences of all clusters were placed onto the corresponding reference gene family tree from EggNOG 4.5.1. The branch with the highest score per cluster was assumed as the taxonomic annotation and putative source of a transfer. If no homologs outside the sampled species were present, genes were labelled as de-novo. Specific placements were summarized into larger taxonomic groups. In total 88 gene clusters were inferred originations for more than one node, and were treated independently for visualization and are listed separately.
